# Association of imputed prostate cancer transcriptome with disease risk reveals novel mechanisms

**DOI:** 10.1038/s41467-019-10808-7

**Published:** 2019-07-15

**Authors:** Nima C. Emami, Linda Kachuri, Travis J. Meyers, Rajdeep Das, Joshua D. Hoffman, Thomas J. Hoffmann, Donglei Hu, Jun Shan, Felix Y. Feng, Elad Ziv, Stephen K. Van Den Eeden, John S. Witte

**Affiliations:** 1 Program in Biological and Medical Informatics, University of California San Francisco, San Francisco, CA 94158 USA; 2 Department of Epidemiology and Biostatistics, University of California San Francisco, San Francisco, CA 94158 USA; 3 Department of Urology, University of California San Francisco, San Francisco, CA 94158 USA; 4 Department of Radiation Oncology, Helen Diller Family Comprehensive Cancer Center, University of California San Francisco, San Francisco, CA 94115 USA; 5 Institute for Human Genetics, University of California San Francisco, San Francisco, CA 94143 USA; 6 Department of Medicine, University of California San Francisco, San Francisco, CA 94158 USA; 7 Helen Diller Family Comprehensive Cancer Center, University of California San Francisco, San Francisco, CA 94158 USA; 8 Division of Research, Kaiser Permanente, Northern California, Oakland, CA 94612 USA

**Keywords:** Cancer genomics, Gene expression, Prostate cancer, Prostate

## Abstract

Here we train *cis*-regulatory models of prostate tissue gene expression and impute expression transcriptome-wide for 233,955 European ancestry men (14,616 prostate cancer (PrCa) cases, 219,339 controls) from two large cohorts. Among 12,014 genes evaluated in the UK Biobank, we identify 38 associated with PrCa, many replicating in the Kaiser Permanente RPGEH. We report the association of elevated *TMPRSS2* expression with increased PrCa risk (independent of a previously-reported risk variant) and with increased tumoral expression of the *TMPRSS2*:*ERG* fusion-oncogene in The Cancer Genome Atlas, suggesting a novel germline-somatic interaction mechanism. Three novel genes, *HOXA4*, *KLK1*, and *TIMM23*, additionally replicate in the RPGEH cohort. Furthermore, 4 genes, *MSMB*, *NCOA4*, *PCAT1*, and *PPP1R14A*, are associated with PrCa in a trans-ethnic meta-analysis (*N* = 9117). Many genes exhibit evidence for allele-specific transcriptional activation by PrCa master-regulators (including androgen receptor) in Position Weight Matrix, Chip-Seq, and Hi-C experimental data, suggesting common regulatory mechanisms for the associated genes.

## Abstract (web summary)

In prostate cancer, investigating aberrant gene expression may shed light on disease etiology. Here, the authors imputed expression transcriptome-wide for 233,955 European ancestry men, discovering and replicating the associations between prostatic expression for select genes and prostate cancer risk, including the highly prevalent gene fusion partner *TMPRSS2*. The authors furthermore integrate diverse functional genomic datasets to interpret the epigenetic mechanisms by which the implicated risk variants and genes modulate disease risk.

## Introduction

Prostate cancer remains a leading cause of cancer incidence and mortality worldwide, with 1.6 million new cases and 366,000 deaths annually^1^. Although prostate-specific antigen (PSA) screening was associated with a 51% reduction in PrCa mortality in the United States between 1993 and 2014^2^, the 5-year survival for patients with metastatic PrCa is 29%^3^. Identifying novel genetic predictors of PrCa may facilitate improvements to early detection and elucidate the mechanisms influencing carcinogenesis. While previous studies have used enhancer assays^4^ and expression quantitative trait locus (eQTL) associations^5^ to propose gene targets for PrCa risk loci, these approaches neither consider the complex genetic architecture of gene expression^6^ nor validate findings in large external cohorts. In pursuit of a comprehensive, systematic characterization of the genes regulated by germline PrCa risk variants, we performed a transcriptome-wide association study (TWAS) of PrCa risk. Our study sought to model prostatic gene expression in the large number of normal prostate tissue samples, in contrast to a recently published PrCa TWAS that modeled prostatic expression using other tissues and fewer normal prostate samples^7^. Here we present our analyses, leveraging data from hundreds of thousands of subjects from the UK Biobank and Kaiser Permanente (Supplementary Tables 1–2), as well as ChIP-Seq, DNAse-Seq, Hi-C, Transcription Factor Binding Matrices, and tumoral expression to identify and interpret the transcriptional and disease risk mechanisms for putative PrCa risk genes.

## Results

### Training and validation of novel prostatic expression models

**Fig. 1:**
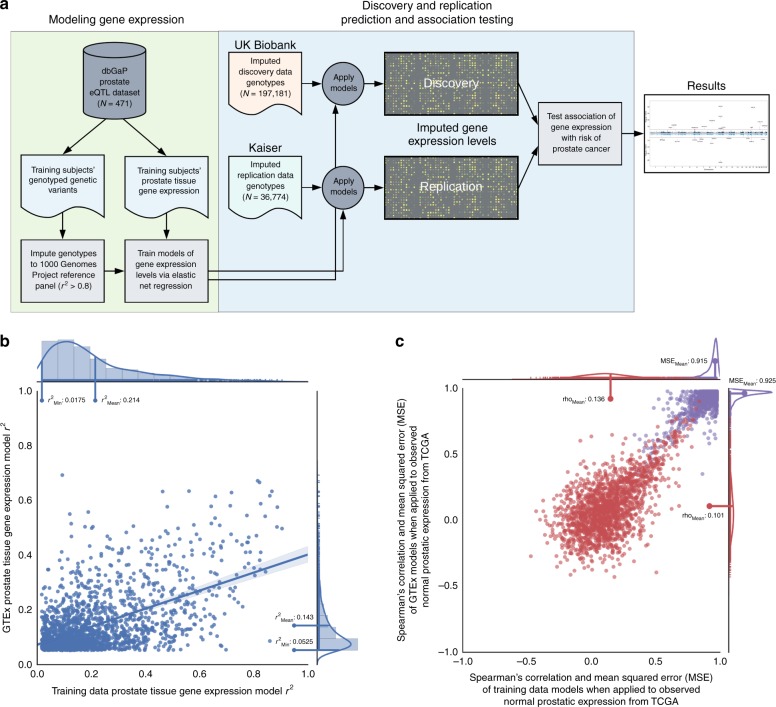
TWAS experimental design and comparison of reference panel model performance. **a** Experimental design for TWAS study of prostate cancer risk. **b** Scatter plot comparison of the cross-validated performance *r*^2^ for 1884 gene expression models derived from GTEx prostate data (*N* = 87 subjects) vs. the training dataset for the present study (*N* = 471). In addition to a linear regression line and 95% confidence interval, marginal histograms and density curves are included for both the *x*-axis (training data model performance) and *y*-axis (GTEx model performance), with the minimum and mean *r*^2^ values also labeled. Performance *r*^2^ was computed based on in-sample cross-validation in each respective dataset. **c** Scatter plot comparison of the out-of-sample model performance for models derived from GTEx vs. the training dataset. Both sets of models were applied to a TCGA normal prostate tissue dataset (*N* = 45) to measure the relationship between observed and imputed expression for 1753 genes. The correlation (Spearman’s rho) between imputed and observed expression is illustrated in red, while the mean squared error of the predictions is illustrated in violet, both with marginal density curves

To estimate genetically regulated expression among the study subjects, we developed novel models using a large number of samples (*N* = 471 subjects; Fig. 1a) with paired prostate tissue gene expression measurements and germline genotypes^5^. These models improve upon the commonly used Genotype-Tissue Expression (GTEx, v6p) dataset^6^, which includes many fewer prostate samples (*N* = 87). Specifically, in comparison to GTEx (Supplementary Fig. 1, Supplementary Table 3), our expression models successfully fit substantially more genes (13,258 vs. 2491 genes), and had a significant increase in the average cross-validated prediction *r*^2^ (mean 0.214 vs. 0.143, *p* = 6.59 × 10^–89^; Fig. 1b, for 1884 overlapping genes) while maintaining a similar number of eQTL predictors (mean 31.1 vs. 32.7, *t*-test *p* = 0.05; Supplementary Fig. 2). We also compared our models to GTEx in a third independent dataset of normal prostatic expression and germline genotypes from The Cancer Genome Atlas (TCGA; *N* = 45; Fig. 1c). Here, our models exhibited a significant decrease in the out-of-sample mean squared error (mean 0.915 vs. 0.925, *t*-test *p* = 1.19 × 10^–12^; Spearman’s rho [Bootstrap 95% CI]: 0.452 [0.409, 0.492], *p* = 3.51 × 10^–89^) and increase in the Spearman’s correlation between predicted and observed expression (mean 0.136 vs. 0.101, *t*-test *p* = 2.36 × 10^–15^; Spearman’s rho [Bootstrap 95% CI]: 0.518 [0.479, 0.556], *p* = 1.86 × 10^–121^). Finally, our restriction of modeled genotypes to variants within 500 kb of gene boundaries rather than 1 Mb, as implemented by PredictDB^6^, gave a similar out-of-sample predictive accuracy of TCGA normal expression (mean Spearman’s rho = 0.077 vs. 0.074, *t*-test *p* = 0.22; Supplementary Fig. 3).

### TWAS testing and validation reveals novel associations

**Fig. 2:**
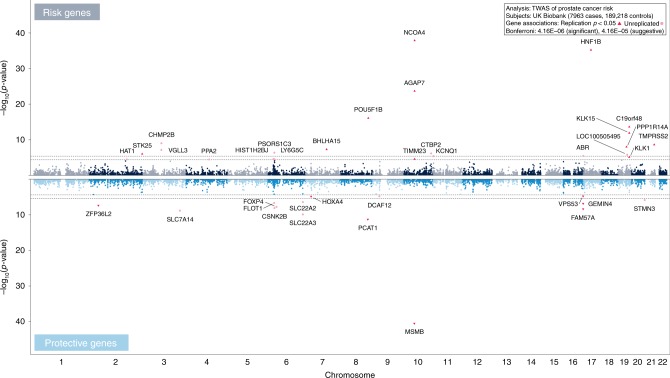
TWAS Discovery Associations. Two Manhattan plots depicting the transcriptome-wide associations with prostate cancer risk for genes with a positive direction of effect (“Risk Genes”, top) and genes with a negative direction of effect (“Protective Genes”, bottom) in the UK Biobank discovery cohort (*N* = 7963 prostate cancer cases, 189,218 male controls). For both Manhattan plots, the associations (Logistic Regression -log_10_(*p*-value), *y*-axis) are plotted against the chromosome and position (*x*-axis) of the transcription start site of a given gene, with non-significant genes on odd and even chromosomes colored in alternating shades. Thresholds for significant (*p* < 4.16 × 10^−6^) and suggestive (4.16 × 10^−6^ < *p* < 4.16 × 10^−5^) associations are illustrated by dashed gray lines, and genes nominally significant (*p* < 0.05) or unreplicated in the Kaiser Permanente RPGEH replication cohort are illustrated as red triangles and pink circles, respectively

We applied our expression models to male subjects from the UK Biobank cohort (7963 PrCa cases, 189,218 controls; Supplementary Table 1) and undertook a TWAS, which found a total of 29 genes with Bonferroni-significant associations (Logistic Regression *p* < 4.16 × 10^–6^), 9 genes with suggestive associations (*p* < 4.16 × 10^–5^), and *λ*_GC_ = 1.146^8^ (*λ*_1000_ = 1.01^9^) (Fig. 2 and Supplementary Fig. 4; Table 1 and Supplementary Table 4). These associations were insensitive to the exclusion of rare variants imputed into the UK Biobank data using the UK10K and 1000 Genomes reference panels (Spearman’s rho = 1.0 for the 38 genes upon exclusion of 160/867 (18.5%) variants modeled, *p* = 4.27 × 10^–78^) in the July 2017 UK Biobank release. Among these 38 genes, 13 replicated at a Bonferroni significance level (Logistic Regression *p* < 0.0013) with directions of effect consistent with the discovery findings in a cohort of unrelated, non-Hispanic white Kaiser Permanente health plan members (6653 PrCa cases, 30,121 controls), and an additional six were nominally significant (*p* < 0.05; Table 1). No difference in model *r*^2^ (*t*-test *p* = 0.91) or the number of modeled variants (*t*-test *p* = 0.24) was observed for these 19 genes, which include previously known and novel findings.

**Table 1:** Discovery and replication analysis summary statistics for significant and suggestive genes

Gene	Discovery (UK Biobank) Beta (SE); *p*-value	Replication (KP) Beta (SE); *p*-value	Model *r*^2^	Locus	Meta *p*-value
MSMB	−1.63 (0.12); 2.97 × 10^−41^	−1.48 (0.14); 1.68 × 10^−25^	0.124	10q11.22	7.00 × 10^−65^
NCOA4	0.75 (0.06); 1.34 × 10^−38^	0.66 (0.06); 6.50 × 10^−25^	0.402	10q11.22	1.53 × 10^−61^
HNF1B	2.03 (0.16); 5.89 × 10^−36^	1.76 (0.19); 1.50 × 10^−20^	0.145	17q12	1.50 × 10^−54^
AGAP7	1.21 (0.12); 2.05 × 10^−24^	0.60 (0.10); 7.88 × 10^−9^	0.204	10q11.22	1.90 × 10^−28^
POU5F1B	3.64 (0.44); 8.40 × 10^−17^	3.42 (0.53); 1.11 × 10^−10^	0.033	8q24.21	6.44 × 10^−26^
C19orf48	2.95 (0.39); 2.46 × 10^−14^	2.04 (0.40); 2.50 × 10^−7^	0.150	19q13.33	1.34 × 10^−19^
KLK15	1.65 (0.23); 1.26 × 10^−12^	1.22 (0.27); 4.57 × 10^−6^	0.056	19q13.33	6.05 × 10^−17^
PCAT1	−1.28 (0.18); 5.01 × 10^−12^	−1.41 (0.21); 1.85 × 10^−11^	0.072	8q24.21	6.47 × 10^−22^
TMPRSS2	0.50 (0.08); 2.42 × 10^−9^	0.24 (0.08); 3.33 × 10^−3^	0.154	21q22.3	3.84 × 10^−10^
FAM57A	−0.50 (0.08); 4.23 × 10^−9^	−0.26 (0.10); 7.49 × 10^−3^	0.376	17p13.3	5.69 × 10^−10^
PPP1R14A	1.80 (0.31); 9.99 × 10^−9^	1.48 (0.37); 6.07 × 10^−5^	0.206	19q13.2	3.31 × 10^−12^
ZFP36L2	−4.06 (0.74); 4.26 × 10^−8^	−3.39 (0.87); 9.71 × 10^−5^	0.035	2p21	2.10 × 10^−11^
BHLHA15	1.80 (0.33); 5.18 × 10^−8^	0.79 (0.28); 4.24 × 10^−3^	0.067	7q21.3	1.34 × 10^−8^
GEMIN4	−2.16 (0.41); 1.39 × 10^−7^	−1.45 (0.48); 2.65 × 10^−3^	0.080	17p13.3	2.52 × 10^−9^
STK25	4.97 (1.02); 9.85 × 10^−7^	3.80 (1.01); 1.76 × 10^−4^	0.100	2q37.3	9.82 × 10^−10^
KLK1	0.36 (0.08); 7.71 × 10^−6^	0.31 (0.07); 6.24 × 10^−6^	0.143	19q13.33	2.27 × 10^−10^
HOXA4	−5.71 (1.31); 1.43 × 10^−5^	−1.89 (0.94); 0.04	0.067	7p15.2	3.13 × 10^−5^
VPS53	−2.30 (0.53); 1.68 × 10^−5^	−1.40 (0.51); 5.79 × 10^−3^	0.259	17p13.3	6.90 × 10^−7^
TIMM23	3.31 (0.79); 2.77 × 10^−5^	3.46 (0.93); 1.89 × 10^−4^	0.080	10q11.22	2.01 × 10^−8^

Three of the most strongly associated genes—*MSMB* (*β*_Discovery_ = −1.63), which encodes the PSP94 tumor suppressor and PrCa biomarker^10^, *NCOA4* (*β*_Discovery_ = 0.75), an androgen receptor co-activator, and *AGAP7* (*β*_Discovery_ = 1.21)—are known targets for the 10q11.22 GWAS variant rs10993994^11,12^ (Table 1). Other previously known PrCa genes that replicated here are: *C19orf48* (*β*_Discovery_ = 2.95) and *KLK15* (*β*_Discovery_ = 1.65), which are upregulated in PrCa in response to androgen levels^4,13,14^, and *POU5F1B* (*β*_Discovery_ = 3.64) and *PCAT1* (*β*_Discovery_ = −1.28), which are known targets of an enhancer at 8q24 in PrCa cell lines^15^ (Table 1).

Furthermore, the following genes exhibited significant associations with PrCa in the discovery and have been reported as targets of PrCa risk loci or microRNAs: *HNF1B* (*β*_Discovery_ = 2.03), *FAM57A* (*β*_Discovery_ = −0.50), *PPP1R14A* (*β*_Discovery_ = 1.80), *GEMIN4* (*β*_Discovery_ = −2.16), *BHLHA15* (*β*_Discovery_ = 1.80), *ZFP36L2* (*β*_Discovery_ = −4.06)^4,5,16–18^. Moreover, *STK25* (*β*_Discovery_ = 4.97), which is differentially expressed in PrCa in comparison to benign prostatic hyperplasia (BPH)^19^, was significantly associated and replicated, while *VPS53* (*β*_Discovery_ = −2.30), known to be regulated by the 17p13 PrCa risk locus^5^, had a suggestive *p*-value in the discovery and was nominally associated in the replication cohort.

The most noteworthy of those associations for which expression in normal prostate tissue has not previously been implicated in prostate tumorigenesis was *TMPRSS2* (*β*_Discovery_ = 0.50; *p*_Meta_ = 3.84 × 10^–10^). Somatically, *TMPRSS2* is part of the most recurrent aberration known in prostate tumors, the *TMPRSS2*:*ERG* (T2E) gene fusion^20^; however, the association of its heritable *cis*-regulatory elements with prostate cancer development is novel. The T2E chromosome 21 structural fusion variant places the *ERG* oncogene under the transcriptional control of the *TMPRSS2* promoter, which is primarily active in prostate tissue.

Several additional genes not previously linked to PrCa susceptibility were identified, including *KLK1* (*β*_Discovery_ = 0.36), *TIMM23* (*β*_Discovery_ = 3.31), and *HOXA4* (*β*_Discovery_ = −5.71). *KLK1* (*p*_Meta_ = 2.27 × 10^–10^), located at 19q13.33 close to the PSA encoding gene *KLK3*, was significantly associated, while *TIMM23* (*p*_Meta_ = 2.01 × 10^–8^), located at 10q11.22, and *HOXA4* (*p*_Meta_ = 3.13 × 10^–5^) had suggestive *p*-values in the discovery cohort and were nominally associated in the replication analysis. *TIMM23* was not previously shown to have significant differential PrCa expression or eQTL activity^11,12^, and *HOXA4* has been implicated in ovarian cancer^21^ and leukemia^22^.

### Conditional and trans-ethnic meta analyses of associations

**Fig. 3:**
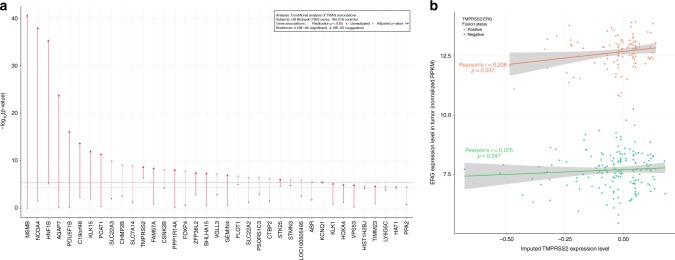
TWAS analysis conditional upon prostate cancer risk GWAS variants and correlation between imputed *TMPRSS2* expression and observed *ERG* expression in TCGA prostate tumors. **a** Comparison of the associations in the UK Biobank discovery cohort before (red or pink) and after (blue) adjusting a gene’s association (*y*-axis, −log_10_(*p*-value)) for the genotypes at the previously reported lead variant for an adjacent prostate cancer risk GWAS locus. When the lead variant was not present in the imputed UK Biobank genotype dataset, a suitable proxy (*r*^2^ > 0.8 in 1000 Genomes Phase III EUR) was used if available. The *p*-value threshold for Bonferroni-corrected significance (Logistic Regression *p* < 4.16 × 10^−6^) is illustrated by a dashed black line, and the suggestive *p*-value threshold by a dashed grey line. Genes nominally significant (*p* < 0.05) or unreplicated in the Kaiser Permanente RPGEH replication cohort are illustrated as red triangles and pink circles, respectively. **b** Scatter plot illustrating the relationship between imputed expression of *TMPRSS2* in normal prostate tissue as predicted by germline *cis*-eQTL genotypes (*x*-axis) and observed tumoral expression of *ERG* (*y*-axis) in prostate cancer cases from The Cancer Genome Atlas (TCGA). Data are colored by *TMPRSS2:ERG* (T2E) fusion status for T2E-positive (orange, *N* = 101) and T2E-negative (green, *N* = 161) subjects, as inferred from paired-end RNA-Seq data. Linear regression lines and 95% confidence intervals illustrate the respective means and trends for T2E-positive and T2E-negative subjects

To account for the influence of proximally located PrCa susceptibility loci, conditional analyses were carried out in the UK Biobank cohort with adjustment for independent (linkage disequilibrium (LD) *r*^2^ < 0.2 in 1000 Genomes Phase III EUR) PrCa risk variants within 5 Mb of the genes tested. Models for *KLK1* and *KLK15* were also adjusted for rs17632542, a missense variant in *KLK3* representing the lead PSA signal in this region^23^. Conditional associations were substantially attenuated for most genes; however, *TMPRSS2* remained Bonferroni-significant (Fig. 3a and Supplementary Table 5). Furthermore, as expression of neighboring genes may be correlated, we fit mutually adjusted models that included all genes within the same cytogenetic locus (Supplementary Table 6). For most regions, adjustment for nearby genes attenuated the associations with PrCa risk. For *KLK1* in particular, a substantial proportion (52.5%, 95% CI: [31.7, 91.0]) of the observed susceptibility signal was mediated by *KLK15*.

We further applied our models to impute expression and evaluate associations for the 19 genes of interest among African American, East Asian, and Latino subjects from Kaiser Permanente (1485 cases, 7632 controls; Supplementary Table 2). In a trans-ethnic meta-analysis of the results, *MSMB* and *NCOA4* were Bonferroni significant (*p* < 0.0013), while *PPP1R14A* (*p* = 0.0046) and *PCAT1* (*p* = 0.0057) were both suggestive (Supplementary Table 7). These genes comprised 4 of the 5 with a direction of effect consistent across each ethnic group and concordant with the discovery and replication cohorts. Additionally, for 16 of the 19 genes, the meta-direction of effect was concordant with the discovery and replication analyses.

### Association of *TMPRSS2* expression suggests novel mechanism

In order to better interpret the biological mechanisms by which these genes and others interact to modulate prostate cancer risk, we sought to analyze the relationships between their imputed expression and previously characterized tumor molecular phenotypes using a published catalog of somatic gene fusion events^24^ in subjects with prostate cancer from The Cancer Genome Atlas (TCGA)^25^. Although imputed expression levels of the 19 genes of interest were not significantly associated with previously reported TCGA molecular subtypes of prostate cancer (Supplementary Table 8), one gene in particular, given its involvement with roughly 50% of prostate cancer tumors^20^, merited further investigation in this regard: *TMPRSS2*.

Although the established 21q22.3 PrCa risk variant rs1041449 is only 20 kb away from *TMPRSS2*, previous work found that this variant was not correlated with *TMPRSS2* expression in prostate tumors or normal prostate tissue^26^. More recent work found that rs1041449 was weakly correlated with an eQTL for *TMPRSS2* (LD *r*^2^ < 0.2)^5^. Similarly, adjusting for rs1041449 in our conditional analysis did not materially weaken the *TMPRSS2* association. Hence, our findings indicate the presence of a novel independent susceptibility mechanism in the 21q22.3 PrCa risk locus mediated by regulation of *TMPRSS2* expression.

To investigate the relationship between the germline variants involved in regulating *TMPRSS2* expression levels and the *TMPRSS2*:*ERG* fusion oncogene, we applied our model of *TMPRSS2* expression to the germline genotypes of TCGA prostate cancer cases to impute *TMPRSS2* gene expression. We found that, when considering 101 T2E-positive specimens carrying the gene fusion, predicted levels of *TMPRSS2* expression in normal prostate tissue were positively correlated with observed *ERG* expression levels as measured by RNA-Seq, a proxy for the expression levels of the T2E fusion (Pearson’s *r* [95% CI] = 0.208 [0.013, 0.387], Linear Regression *p* = 0.037; residual Shapiro-Wilks *p* = 0.138, Fig. 3b). In contrast, among 161 T2E-negative TCGA specimens, predicted *TMPRSS2* expression levels were not significantly correlated with observed levels of *ERG* expression (Pearson’s *r* [95% CI] = 0.075 [−0.081, 0.227], *p* = 0.347; residual Shapiro-Wilks *p* = 0.771). Moreover, imputed *TMPRSS2* expression was uncorrelated with observed *ERG* expression in tumor-adjacent normal prostate tissue in both the training dataset (*N* = 471; Pearson’s *r* [95% CI]: 0.031 [−0.060, 0.121], Linear Regression *p* = 0.508; residual Shapiro-Wilks *p* = 0.112) as well as normal prostatic expression data from T2E-positive (*N* = 17; Spearman’s rho [Bootstrap 95% CI]: 0.047 [−0.484, 0.481], *p* = 0.859) and T2E-negative subjects (*N* = 28; Spearman’s rho [Bootstrap 95% CI]: −0.183 [−0.373, 0.382], *p* = 0.351) from TCGA. Further testing of the association of predicted *TMPRSS2* levels with T2E fusion status (positive vs. negative) across all 262 samples did not reveal an association (Logistic Regression *p* = 0.448), and tumoral *AR* expression was uncorrelated with T2E fusion status (Logistic Regression *p* = 0.882). These findings suggest a germline-somatic interaction mechanism whereby germline variation may mediate cancer risk through its effect on the burden of a somatic driver: the *TMPRSS2*:*ERG* fusion oncogene (Supplementary Fig. 5).

### Common androgen-driven mechanisms regulate TWAS associations

To clarify the transcriptional mechanisms of PrCa risk eQTLs, we examined the transcription factor (TF) occupancy of our modeled eQTL variants. Among the 19 genes with nominal replication, 13 showed evidence for transcriptional regulation by master regulators of PrCa gene expression in ChIP-Seq data for the prostate cell line VCaP (Table 2)^27^. Seven genes had at least one eQTL in a transcription factor binding site (TFBS) for androgen receptor (AR), a sentinel of prostatic expression, while one gene (*PCAT1*) had an eQTL in a TFBS for SPDEF, a prognostic marker for PrCa survival involved in AR regulation^28^, and the remaining five had eQTLs highly correlated with variants in an AR TFBS (LD *r*^2^ ≥ 0.8). In contrast, only 30 of 100 genes selected at random showed any evidence of a VCaP ChIP-Seq TFBS for AR, SPDEF, or ERG (Supplementary Table 9), despite the 100 genes being significantly larger on average (81.7 kb) than the 19 genes of interest (25.1 kb; *t*-test *p* = 0.0065). Hence, we observed a significant enrichment of prostate-specific regulation at, or in proximity to, eQTL variants for these 19 associated genes (Fisher’s Exact *p* = 0.0031; Bootstrap OR_Enrichment_ [95% CI] = 5.16 [1.82, 20.17]).

**Table 2:** Replicated genes with eQTLs in or tagging VCaP ChIP-Seq transcription factor binding sites

Gene	VCaP ChIP-Seq TFBS	Variant(s) (hg19 position)
AGAP7	AR	rs58186870 (chr10:51812898), rs58677292 (chr10:51812896), rs56106241 (chr10:51812825)
BHLHA15	AR	rs6975156 (chr7:97925533), rs7789380 (chr7:97956179), rs10953245 (chr7:97855461)
C19orf48	AR	rs11665748^a^ (chr19:51354396), rs78177998^a^ (chr19:51345263), rs2659051^a^ (chr19:51345567), rs11665698 (chr19:51354410)
FAM57A	AR	rs461251^a^ (chr17:619161), rs684232^a^ (chr17:618964)
GEMIN4	AR	rs461251^a^ (chr17:619161), rs684232^a^ (chr17:618964)
KLK1	AR	rs11084033^a^ (chr19:51353954)
KLK15	AR	rs78177998^a^ (chr19:51345263)
NCOA4	AR	rs12571566 (chr10:51813068), rs61848292 (chr10:51813024), rs12569965 (chr10:51813070)
PCAT1	SPDEF	rs1516942 (chr8:128019902), rs28615829 (chr8:128018204), rs7844107^a^ (chr8:128023385), rs73351621 (chr8:128014414), rs9693379 (chr8:128022940), rs78316206^a^ (chr8:128019308), rs2035637^a^ (chr8:128023058), rs17830059 (chr8:128016372), rs73351629 (chr8:128018465), rs16901898 (chr8:128015091)
PPP1R14A	AR	rs73034946 (chr19:38460492)
STK25	AR	rs56390510^a^ (chr2:242274488)
TMPRSS2	AR	rs56095453^a^ (chr21:42893807), rs8134378 (chr21:42893757), rs8134657 (chr21:42893907)
VPS53	AR	rs461251^a^ (chr17:619161), rs684232^a^ (chr17:618964)

^a^Directly modeled eQTL variants in VCaP ChIP-Seq TFBS. Remaining variants in LD (*r*^2^ ≥ 0.8 in 1000 Genomes Phase III EUR) with a modeled eQTL variant

**Fig. 4:**
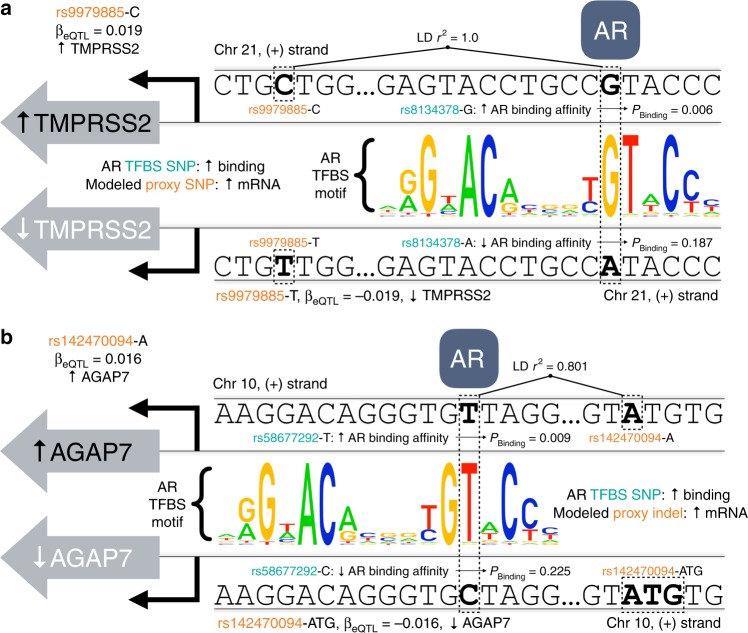
Comparison of variant effect on androgen receptor (AR) TFBS affinity and modeled eQTL effect on gene expression levels. **a** Illustration of the relationship between the effect of variant rs9979885 (orange) on prostatic *TMPRSS2* expression levels (*β*_eQTL_), estimated from elastic net regression, and the effect of rs8134378 (teal) on AR binding (*p*_Binding_). In determining predictors of *TMPRSS2* levels in normal prostate tissue, the penalized regression model selects rs9979885, a perfect LD proxy for rs8134378. As depicted by binding motif allele frequencies in the AR TFBS motif sequence logo and previously validated experimentally, the rs8134378-G allele significantly improves the affinity of AR binding in comparison to the rs8134378-A allele, substantially improving the probability of AR occupancy (*p*_Binding_ = 0.006 vs. 0.187, using TRANSFAC vertebrate matrix V$AR_01, in comparison to human promoter background) according to sTRAP transcription factor affinity prediction modeling. Likewise, the rs9979885-C allele, in total linkage disequilibrium (LD *r*^2^ = 1.0 in 1000 Genomes Phase III EUR) with rs8134378-G, is predicted to increase expression of TMPRSS2 (located on the reverse-strand of chromosome 21), in comparison to the rs9979885-T allele. The correlation between the alleles estimated to increase transcription factor binding and gene expression reflects the model’s biologically relevant and mechanistic ascertainment of the effect of AR binding on *TMPRSS2* expression. **b** Illustration of the relationship between the effect of variant rs142470094 (orange) on prostatic *AGAP7* expression (*β*_eQTL_) and the effect of rs58677292 (teal) on AR binding (*p*_Binding_). As depicted by the AR TFBS motif sequence logo, the rs58677292-T allele significantly improves the affinity of AR binding in comparison to the rs58677292-C allele, increasing the probability of AR occupancy (*p*_Binding_ = 0.009, vs. 0.225, using TRANSFAC Vertebrate Matrix V$AR_01) according to sTRAP Modeling. Likewise, the rs142470094-A allele, in high linkage disequilibrium (LD *r*^2^ = 0.801 in 1000 Genomes Phase III EUR) with rs58677292-T, is predicted to increase expression of *AGAP7* (located on the reverse-strand of chromosome 10) in comparison to the rs142470094-ATG indel, suggesting that *AGAP7* may be regulated in part by genetic effects on androgen receptor binding

Similar to a previous report of disrupted AR binding at LD proxies for PrCa GWAS peaks^29^, inclusion of variants in high LD with the modeled eQTLs revealed additional AR and SPDEF binding sites, including at a known androgen-responsive enhancer variant for *TMPRSS2* rs8134378^30^. Among the 31 variants in AR and SPDEF TFBS, 3 variants (rs8134378, rs11084033, and rs2659051) were annotated in the NCBI LitVar database^31^ with published reports corroborating their AR occupancy^30,32,33^. When cross-referenced with H3K27ac active-enhancer marks from 19 primary prostate tumors^29^, these 31 TFBS variants were significantly enriched at H3K27ac ChIP-Seq peaks (mean [SD]: 8.35 peaks [7.86]) in comparison to variants selected at random (*N* = 10,000) from the Haplotype Reference Consortium r1.1 site list (mean [SD]: 0.59 peaks [2.71]; *t*-test *p* = 9.35 × 10^–45^). Additionally, for 17 of the 21 variants in VCaP AR ChIP-Seq peaks, the allele predicted to increase AR binding affinity^34^ was the same allele, or in high LD (*r*^2^ ≥ 0.8) with the eQTL allele, predicted to increase target gene expression (Binomial *p* = 0.0072; Supplementary Table 10), including the rs8134378 *TMPRSS2* enhancer variant and rs9979885, an AR TFBS variant in high LD with an *AGAP7* eQTL (Fig. 4). Collectively, this evidence illustrates an androgen-responsive mechanism of allele-specific enhancer activity for the variants and genes implicated.

### Multi-omics pathway-based TWAS enrichment and interpretation

Furthermore, DNAse-seq footprinting from the PrCa cell line LNCaP^35^ revealed recurrent motifs for E2F, INSM1, MEF-2, VDR, and ZFX (Supplementary Table 11), several of which have known involvement in PrCa development or progression^36–38^, at the eQTL variants for the 19 genes of interest. In addition, ChIP-Seq annotations from non-prostate cell lines included motifs for 150 TF’s, including recurrent CTCF, HNF4A, MYC, POLR2A, and SIN3A motifs. A Reactome pathway enrichment analysis^39,40^ of all 150 TF’s yielded numerous significant associations (FDR-adjusted *p*-value < 5.00 × 10^–7^) in several pathway hierarchies relevant to transcription, epigenetics, and oncogenesis (Supplementary Table 12). Furthermore, TFBS inferred from DNAse-seq footprinting in non-prostate cell lines or Position Weight Matrices (PWM) included recurrent motifs for SRF, ZFP105, ELF3, FOXP1, and TCFAP2E, some with known roles in PrCa regulation or prognosis^41–43^.

Chromatin conformation capture data (Hi-C) from LNCaP^44^ supported promoter-enhancer interactions between our modeled eQTLs and their respective target genes. In particular, virtual 4C interactions covered the positions of the modeled *cis*-eQTLs furthest upstream and downstream of 17 of the 19 genes of interest (Supplementary Fig. 6). The two exceptions, *AGAP7* and *NCOA4*, had tighter distributions of Hi-C read values in proximity to the GWAS variant rs10993994, which attenuated both associations substantially in our conditional analysis, further supporting previous evidence for the regulation of *AGAP7* and *NCOA4* by the 10q11.22 GWAS locus^11,12^.

## Discussion

The TWAS framework^6^ leveraged here offers a simple yet elegant method to explore the effects of gene expression on disease risk. Although it has been suggested that TWAS are prone to inflation and bias of test statistics^45^, our sample size-adjusted inflation factor did not indicate inflation (*λ*_1000_ = 1.01). Furthermore, while field effects may modulate the molecular characteristics of tumor-adjacent normal prostate tissue^46^, our integration of paired genotype and expression data in a large number of training samples supports the robustness of our models against such molecular perturbations, in particular for a heterogeneous disease like prostate cancer^47^. Moreover, in order to guard against bias or inflation and support the validity of our findings, we performed a formal replication analysis in a large cohort. While the penalized regression models used here may improve the model interpretability and parsimony through regularization, these models still face the challenge of selecting causal predictors among many highly correlated or collinear variables. Our analyses of experimental and patient data illustrate how surveying the epigenomic landscape in proximity to TWAS model predictors may elucidate causal regulatory mechanisms that evade feature selection.

It is noteworthy that the consideration of tissue that appears histopathologically normal and yet harbors somatic aberrations due to field effects, although a more conservative control in the context of germline-somatic comparisons, may impinge upon the detection of significant germline-somatic mechanisms. Stringent quality control that restricts normal samples to those with limited tumor cellularity may increase statistical power in this context. Yet, innovative biological systems modeling to experimentally validate the interactions between germline risk polymorphisms and the earliest somatic drivers of carcinogenesis (such as the *TMPRSS2*:*ERG* fusion oncogene) are necessary to further the lines of inquiry advanced by this study and others^26,48^. In particular, reports have suggested that the presence of the *TMPRSS2*:*ERG* gene fusion in high-grade prostatic intraepithelial neoplasia (HG-PIN) may be a harbinger of T2E fusion-positive prostate cancer^49^; hence, HG-PIN may represent a suitable model system for this mode of discovery. Finally, our results demonstrate the utility of generating larger TWAS reference panels to produce better performing models of gene expression and facilitate the discovery of disease associated genes.

In summary, we present results from a large-scale TWAS of PrCa that detected multiple novel mechanisms of gene expression and disease risk modulation. In addition to in silico experimental support for our findings, certain genes implicated in our study replicate prior TWAS findings (*BHLHA15*, *AGAP7*, *NCOA4*, *VPS53*, *FAM57A*, *GEMIN4*, *PPP1R14A*)^7^ or prostate cancer literature, and the directions of effect in our study for previously reported cancer genes are largely concordant with the prior literature. The protective genes reported here have generally been measured or predicted to be downregulated in PrCa (*FAM57A*, *GEMIN4*, *VPS53*)^5^ or are suspected tumor-suppressors (*MSMB*, *HOXA4*)^10,33^. Notably, both tumor-promoting and tumor-suppressive effects have been observed for *HNF1B*^50,51^ and the protein product TIS11D of *ZFP36L2*^17,52^. However, the estimated protective direction of effect observed for *PCAT1* contradicts previous characterization^53^ of this RNA oncogene. Although discordance between eQTL risk effects and disease-specific differential expression has been previously reported^54^, the mechanisms underlying these inconsistencies remain to be elucidated. Collectively, our findings integrate data from diverse multi-omic assays to elucidate a network of genes, many androgen-regulated including *TMPRSS2*, and transcription factors active in PrCa. Joint consideration of the respective nodes and edge-relationships that comprise this network may provide a more comprehensive interpretation of the genetic and molecular etiology of PrCa and clarify directions for future modeling and investigation.

## Methods

### Statistical tests

All statistical tests conducted were two-sided.

### Study populations

Subject data used for discovery and replication analyses are summarized in Supplementary Table 1.

### Prediction of gene expression

Samples used to develop our regularized models of prostate tissue gene expression were drawn from the National Center for Biotechnology Information (NCBI) publicly available database of Genotypes and Phenotypes (dbGaP phs000985.v1.p1). These data derive from a previous study that extracted DNA and RNA from histologically normal prostate tissue from consenting subjects (471 men; mean age [SD]: 60.1 [7.15] for the 249 men with age available) having undergone radical prostatectomy treatment for prostate cancer (*N* = 453; 63.6% Gleason 6, 36.4% Gleason 7) or cystoprostatectomy treatment for bladder cancer (*N* = 18)^5^. Inclusion criteria were based on a rigorous histopathological evaluation^5^, which included the requirement of Gleason grade less than or equal to 7 in the presenting tumor and the absence of HG-PIN and benign prostatic hyperplasia in the examined fresh frozen normal prostate tissue, among other criteria. Furthermore, the dataset was limited to unrelated subjects of European genomic ancestry. Expression quality control was previously described^5^ and included evaluation of the effect of flowcell, lane, sample groups/plates, gene size, and GC content on sample mRNA abundance and expression level. Furthermore, data were previously^5^ evaluated for quality and normalization bias using graphical methods and residual MA plots, mRNA transcripts with low median gene count (less than 14) were filtered, and the remaining gene counts were quantile normalized^7^.

The first step in our experimental design process (Fig. 1a) was to impute unobserved genotypes for these training data, which included over 1.5 million genotyped variants, limited to common variants (minor allele frequency >1%) in Hardy-Weinberg equilibrium (*p* > 1.00 × 10^–5^) and with a call rate >95%^5^. Prior to imputing these data to the 1000 Genomes Project Phase III reference panel, which performs comparably to larger reference panels for common variants^55^, we used a pre-phasing QC workflow to match the strand and reference allele recorded in the data with those observed in the reference panel, while excluding ambiguous variants and indel mutations. Next, these samples were phased and imputed using Eagle v2.3^56^ (cohort-based) and Beagle v4.1^57^, respectively.

Gene boundaries (hg38) for the 17,233 transcripts measured in the training dataset were downloaded from the NCBI Gene database using the Biopython Entrez eutils REST API^58^. Genomic coordinates were converted from hg38 to hg19 (GRCh37) via UCSC liftOver^59^. For each of these transcripts, well-imputed (*r*^2^_INFO_ > 0.8) training data genetic variants located (a) in the 500 kb region upstream of the start position, (b) between the start and end positions, inclusive, or (c) in the 500 kb region downstream of the end position, were extracted. Next, following the approach of PrediXcan^6^, a regularized regression model was fit using the R (v3.2.2) package GLMNet^60^ with the genetic *cis*-variants as the design matrix and the RNA-Seq transcript levels as the response variable. Additional individual-level covariates such as age were unavailable from dbGaP, but unlikely to bias model-training in light of their independence from germline genotype. Models with at least one non-intercept explanatory variable retained were successfully fit for 13,258 genes, and leave-one-out cross validation (LOOCV) was used (loss function: R *cv.glmnet type.measure*=“mse”) to select model coefficients that minimize mean cross-validated error (regularization parameter: R *predict s*=“lambda.min”) and evaluate model performance *r*^2^ (R *predict s*=“lambda.min”).

LOOCV models performed similarly to those generated by 10-fold cross-validation in application to a third, independent dataset of paired genotypes and normal prostatic expression data (RNA-seq; *N* = 45 total subjects available) from TCGA (Supplementary Fig. 7), while providing a reproducible estimate of *r*^2^ insensitive to fold sampling variation. As previously reported, TCGA normal prostate samples were subjected to pathology review to confirm their prostatic origin and limit the presence of tumor and HG-PIN^25^. Furthermore, a comparison of cross-validated *r*^2^ for elastic net (*α* = 0.5) and LASSO (*α* = 1.0) models showed that the elastic net models were moderately more predictive on average (mean *r*^2^ = 0.138 vs. 0.135; *t*-test *p* = 0.08). Hence, we used the elastic net models for transcriptome imputation.

For each gene, the number of modeled variants and model *r*^2^ in our database were compared to the corresponding entry in the “TW_Prostate_0.5.db” database of GTEx models made available on the PredictDB website’s “GTEx-V6p-HapMap-2016–09–08” repository. To compare the out-of-sample performance of our models against analogous models from GTEx, we again imputed expression in the TCGA normal prostate tissue dataset (*N* = 45) for the 1753 genes present in both our models and GTEx that had expression quantitative trait locus (eQTL) SNPs observed/imputed in TCGA genotypes with *r*^2^_INFO_ > 0.5. We then standardized the distribution of observed expression FPKM’s for each gene, and also standardized the distributions of expression that were imputed using our models and GTEx. Finally, we measured both the mean squared error (MSE) of the standardized imputed distributions of expression in comparison to the true standardized FPKM’s, and additionally measured the correlation (Spearman’s rho) of the standardized, imputed expression values with the true standardized normal prostate expression FPKM’s. We performed the same comparison between our models and a set of models developed from the same input dataset that modeled variants within 1 Mb of gene boundaries. In particular, the correlation/MSE with TCGA expression was compared for 9717 genes present in both sets of models and with eQTL SNPs imputed with *r*^2^_INFO_ > 0.5 in TCGA. Based on the positive performance metrics of the overlapping models in relation to GTEx and TCGA, we carried the full set of our models forward into the TWAS in order to evaluate the significance of any case-control differences and the extent to which such differences were replicated across datasets. Model composition was compared between our models and GTEx, for a set of 10 genes associated in our discovery analysis and present in both databases, by computing and visualizing the proportion of pairwise coverage (LD *r*^2^ > 0.3 in 1000 Genomes Phase III EUR) of the variants in one model by any of the variants in its corresponding model. Heatmaps were generated using the R *superheat* library^61^.

### Transcriptome wide association testing

We undertook our discovery TWAS using data from the publicly available UK Biobank, a cohort of nearly 500,000 adult subjects recruited across the United Kingdom between 2006 and 2010 and receiving healthcare from the UK National Health Service (NHS). Consenting participants contributed blood and urine samples to provide material for high-throughput genotyping and additional bioanalytical assays. Furthermore, the collected information and specimens were linked to lifetime NHS electronic health records, including ICD codes for diagnoses and procedures.

The UK Biobank data includes autosomal genotype data for 488,377 subjects, 223,513 male and 264,864 female. We limited these subjects to individuals with both a self-reported and genetically inferred gender of male. Using KING v2.0^62^, we excluded first-degree relatives while prioritizing the inclusion of cases. To control for the potential confounding effects of ancestry and population structure in this largely ethnically white cohort^63^, subjects were also excluded if they were beyond 5 standard deviations of the means for the first two genetic principal components (Supplementary Fig. 8), leaving 197,181 total subjects for the discovery analysis (mean [SD] age: 57.4 [8.1], BMI: 27.9 [4.2]). Prostate cancer case control status was determined using ICD codes (ICD-9: “185”, ICD-10: “C61”, or “D07.5”) in the UK Biobank cancer registry data, yielding 7963 cases and 189,218 controls.

Imputed genotypes were included with our download of the UK Biobank data. These data were imputed at 33,619,058 variants using the Haplotype Reference Consortium (HRC) reference panel of 64,976 haplotypes^64^, covering the majority of known common variation, using SHAPEIT3 and IMPUTE4 for phasing and imputation, respectively^61^. Additional rare variants not present on the HRC panel (mean (SD) minor allele frequency: 0.008 (0.05), versus 0.04 (0.10) for HRC imputed variants) were imputed using UK10K and 1000 Genomes Project reference panels, bringing the total to 92,693,895 variants imputed. We found that the exclusion of these variants from our discovery analysis had a negligible impact on our results.

Transcript levels were imputed using individual-level data using a modified version of the PrediXcan program^6^. The modifications implemented included allele matching (flipping and/or reverse complement) with direction-of-effect flipping for non-ambiguous variants, as well as parallelized segregation of genes by chromosome. Although modeled variants absent from the imputed discovery genotypes were treated as missing data and omitted from transcriptome imputation, we noted a 92.9% overlap between variants imputed in the training data with *r*^2^_INFO_ > 0.8 and those imputed with *r*^2^_INFO_ > 0.8 in the discovery and replication datasets. Of the 13,258 gene prediction models developed in the training data, 1244 were excluded from further analysis due to the absence of sex chromosome data in the discovery cohort (415 genes) or due to missing genotype data (829 genes). Prediction models for the remaining 12,014 genes were applied to 197,181 discovery subjects, and resulting predictions of gene expression levels were tested for association with prostate cancer risk.

Logistic regression models were used to assess the association between imputed transcript levels and prostate cancer status. To control for confounding, the models were adjusted for several covariates associated with prostate cancer risk, including age, body mass index, and 15 principal components of ancestry and population structure. For prostate cancer cases, age at diagnosis was used, whereas age at assessment was used for controls. Bonferroni correction for the number of genes tested (12,014) was applied to control for multiple hypothesis testing. Hence, genes with a *p*-value less than 4.16 × 10^−6^ were considered to be significantly associated in the discovery analysis, while the threshold for suggestive associations was set at one order of magnitude higher (*p* < 4.16 × 10^−5^). In addition to computing the genomic control inflation factor (*λ*_GC_)^8^, which is known to scale with sample size, we also generated a sample-size adjusted inflation factor (*λ*_1000_) for the discovery *p*-values^9^.

### Replication testing and trans-ethnic meta-analysis

We performed replication analyses in a sample of male Kaiser Permanente health plan members^65^. These data derive from three studies: the Kaiser Permanente Research Program on Genes, Environment, and Health (RPGEH), the ProHealth Study, and the California Men’s Health Study (CMHS). Samples were genotyped on custom, ethnic specific arrays based on self-reported ethnicity and segregated into African American (AFR), East Asian (EAS), European (EUR), and Latino (LAT) analysis groups^66^. Imputation of the replication data to the 1000 Genomes Project reference panel was previously performed using SHAPEIT v2.5 and IMPUTE2 v2.3.1^65,67,68^. Singleton variants were removed from the reference panel due to poor imputation quality, and each array (AFR, EAS, EUR, LAT) was phased separately due to only partial overlap of the SNPs on the different arrays. As noted earlier, while 92.9% of the imputed genetic variants with *r*^2^_INFO_ > 0.8 in the training dataset were also imputed with *r*^2^_INFO_ > 0.8 in the discovery and replication data, those variants absent in the replication genotype data were omitted from transcriptome imputation.

For association analysis, as before, first-degree relatives were excluded while prioritizing the retention of cases. Non-Hispanic White (EUR) subjects (6653 cases, 30,121 controls) were used for replication of the discovery findings (mean [SD] age: 66.3 [11.8], BMI: 27.2 [4.6]). Only the significant and suggestive genes from the discovery analysis were tested for association with prostate cancer case-control status by logistic regression, controlling for age (age at diagnosis for cases, age at assessment for controls), body mass index, and 20 ethnic-specific (i.e., estimated within the ethnic analysis group of interest) principal components of ancestry and population structure. Genes with a replication *p*-value less than 0.05 and a direction-of-effect consistent with the discovery findings were considered nominally replicated, while genes with a replication *p*-value less than 0.0013 were considered to be replicated at a Bonferroni-significance level.

For the genes that replicated nominally, we imputed their expression levels in the AFR, EAS, and LAT subjects (Supplementary Table 2) and evaluated their association with prostate cancer case control status, again using logistic regression adjusted for age, body mass index, and 20 ethnic-specific principal components. These results were aggregated in a fixed-effects meta-analysis using MetaSoft v2.0.0^69^ to produce the trans-ethnic meta-effects and associations for each gene.

### Analysis of *TMPRSS2* expression and TCGA prostate *TMPRSS2*:*ERG*

Germline genotype and molecular phenotype data for prostate cancer subjects from The Cancer Genome Atlas was used to measure the relationship between *TMPRSS2*:*ERG* expression in prostate tumors and imputed *TMPRSS2* expression in the corresponding normal prostate tissue. Tumoral *ERG* expression data from RNA-Seq was downloaded from the UCSC Xena Browser^70^ and *TMPRSS2*:*ERG* (T2E) fusion status was downloaded from a database of TCGA gene fusion events^24^. Genotype data from The Cancer Genome Atlas were downloaded from the NCI Genomic Data Commons^71^ and submitted to the Michigan Imputation Server^72^ (Minimac3 v2.0.1, Eagle v2.3.5) for imputation using the Haplotype Reference Consortium reference panel (HRC r1.1 2016)^64^. Variants with an imputation *r*^2^_INFO_ < 0.5 were excluded from further analysis. In addition to the models for the other 18 genes of interest (Table 1), the *TMPRSS2* prediction model inferred from our training data was applied to the imputed TCGA genotypes. If a modeled eQTL variant was not available, a proxy variant in high LD (*r*^2^ > 0.8 in 1000 Genomes Phase III EUR) was used. Subjects whose RNA samples showed evidence of degradation were excluded^25^. The association between imputed gene expression and TCGA subtype (*ERG* fusion, *ETV1* fusion, *ETV4* fusion, *FOXA1* mutant, *IDH1* mutant, *SPOP* mutant) was evaluated by logistic regression (Supplementary Table 8) using labels derived from the TCGA gene fusion database^24^ and UCSC Xena Browser^70^. Furthermore, a logistic regression model between predicted *TMPRSS2*:*ERG* fusion status and tumoral ERG expression was fit to draw the decision boundary between fusion positive and negative samples. Samples beyond the decision boundary (T2E-positive with *ERG* RPKM < 10.65, or T2E-negative with *ERG* RPKM > 10.65) were excluded to control for fusion status misclassification and reflect the correlation between *ERG* overexpression and T2E fusion status^73^. The correlation between imputed normal and observed tumoral expression was measured via Pearson’s *r*, with the normality of model residuals evaluated by the Shapiro-Wilks test, or Spearman’s rho for limited sample sizes, with 95% confidence interval derived via bootstrap resampling with 10,000 iterations.

### Annotation of eQTL transcription factor occupancy

For each of the genes that were associated and replicated nominally, transcription factor binding site (TFBS) occupancy of their respective eQTL variants was annotated using RegulomeDB v1.1^35^. The dbSNP variant rsid for modeled variants, as well as variants in high LD (*r*^2^ > 0.8 in 1000 Genomes Phase III EUR)^74^, was submitted to the RegulomeDB web portal and results were automatically downloaded and parsed using Selenium webdriver automation. Results were segregated into four descending categories according to their level of evidence and relevance to prostate cancer cell lines VCaP and LNCaP: (1) ChIP-Seq Protein Binding evidence in prostate cancer cell lines, (2) Motif inferred using DNAse-Seq footprinting in prostate cancer cell lines, (3) ChIP-Seq Protein Binding evidence in non-prostate cancer cell lines, and (4) Motif inferred from DNAse-Seq footprinting non-prostate cancer cell lines or predicted using a position weight matrix (PWM). The enrichment of associated genes with evidence in category (1) was evaluated by a Fisher’s exact test in comparison to 100 genes selected at random from our prostate tissue eQTL database, with 10,000 bootstrap resampling iterations to evaluate the median and empirical distribution of the odds ratio. For categories (2) to (4), results were aggregated and tabulated across the genes queried to identify the most recurrent transcription factor binding sites and motifs. While motifs in categories (2) and (4) included the names of many protein families and complexes, category (3) was comprised of HGNC gene names for transcription factors, and served as a suitable input for a pathway analysis. Using PANTHER^39^, we conducted a pathway analysis of Reactome pathway hierarchies^40^, with parameters “organism” = “Homo sapiens”, “Analysis” = “Statistical overrepresentation test” (default settings), “Annotation Data Set” = “Reactome pathways”, and “Test Type” = “Fisher’s Exact with FDR multiple test correction”.

### Evaluation of epigenomic enrichment at eQTL variants

To evaluate the enrichment of eQTL TFBS variants at prostate tissue epigenomic elements, H3K27ac active-enhancer marks were downloaded from 19 primary prostate tumors from the Gene Expression Omnibus (GEO, Accession: GSE96652)^29^. The colocalization of query variant positions with H3K27ac ChIP-Seq BED file intervals was tallied using an SQLite database, and compared to a null distribution of tallies for 10,000 randomly selected variants from the Haplotype Reference Consortium r1.1 site list by a Mann–Whitney–Wilcoxon test.

### Concordance of allele-specific binding with eQTL effects

The correlation between the allele-specific directions of effect on binding affinity and expression levels was examined for variants directly modeled to affect target gene expression, or in high linkage disequilibrium (LD) with a modeled eQTL, for the genes that were associated and nominally replicated. In particular, 25 base pair 3′ and 5′ flanking sequences were extracted from the UCSC table browser^75^ using Selenium webdriver automation for variants present in ChIP-Seq peaks for AR in the VCaP prostate cancer cell line. Next, two FASTA sequences containing the major and minor variant alleles were automatically submitted to the sTRAP Transcription Factor Affinity Prediction webserver^34^, with parameters “matrix file” = “transfac_2010.1 vertebrates”, “background model” = “human_promoters”, and “Multiple test correction” = “Benjamini-Hochberg.” The result, a list of 904 transcription factor binding matrices ranked by the differential effect of the two alleles on binding affinity (as measured by the difference in log_10_(*p*-value) of observing an affinity of a given magnitude or greater under a certain background sequence model), was downloaded and processed. The direction of effect of a particular variant allele A1 on AR binding affinity was estimated using the rank-weighted (“BindingRank”) average over 6 AR binding matrices *m* of the difference in log_10_(*p*-value) in comparison with the opposite allele A2:

1 $$\mathop {\sum }\limits_{m = 1}^{6\;{\mathrm{AR}}\;{\mathrm{matrices}}} \frac{1}{{\sqrt {{\mathrm{Binding}}\,{\mathrm{Rank}}(m)} }}\left( {{\mathrm{log}}_{10}\left( {p_{m,{\mathrm{A}}1}} \right) - {\mathrm{log}}_{10}\left( {p_{m,{\mathrm{A}}2}} \right)} \right)$$

Finally, for each of the variants examined, the allele predicted to increase AR binding affinity was cross-referenced with the estimated effect of that allele, or its proxy allele, on gene expression levels. The concordance of the directions of effect on binding and expression was evaluated via binomial test with probability = 0.5 for the direction of effect.

### Hi-C interaction landscape at eQTL loci for replicated genes

Putative promoter-enhancer interactions between the modeled eQTLs and their respective target genes was analyzed using Hi-C chromatin conformation capture data for the prostate cancer cell line LNCaP from the 3D Genome Browser^44^. A dataset of normalized LNCaP Hi-C read data (“iced-rep-1”) was queried to perform a virtual 4C for each of the genes of interest and generate a Hi-C read density histogram illustrating the physical interactions between a particular region (with the minimum available resolution of 40 kb bins) and its neighboring genomic positions. For each of the genes of interest, the gene name was used as the query and anchoring point, with the exception of one gene (*TIMM23*) where the transcription start site was required to return non-null results. In order to investigate the physical interactions most pertinent to our gene expression models, the genomic positions (hg19/GRCh37) of the modeled eQTL variants (Supplementary Table 13) for each query gene were compared to the virtual 4C boundary of Hi-C read density in the extended region around the anchoring position.

### Ethics statement

The authors declare their compliance with the relevant ethics committees (UC San Francisco, UK Biobank, Kaiser Permanente) and regulations.

## Supplementary information

Supplementary Information

## Data Availability

The reference data used to train gene expression models are available via the NCBI database of Genotypes and Phenotypes (dbGaP: www.ncbi.nlm.nih.gov/gap; Study Accession: phs000985.v1.p1). The UK Biobank data are available to approved researchers registered with the UK Biobank. Genotype data for participants of the Kaiser Permanente RPGEH Genetic Epidemiology Research on Aging (GERA) project are available for the 78% of GERA participants that consented to submit their data to dbGaP (Study Accession: phs000674.v2.p2). The complete GERA data, including cancer phenotypes, are available upon application to the KP Research Bank Portal. Data from The Cancer Genome Atlas are available on dbGaP (Study Accession: phs000178.v1.p1). Data generated during this study are available at www.github.com/Wittelab/PrCa_TWAS.

## Code availability

Analysis code is available at www.github.com/Wittelab/PrCa_TWAS.
