# Author Correction: Association of imputed prostate cancer transcriptome with disease risk reveals novel mechanisms

**DOI:** 10.1038/s41467-019-11810-9

**Published:** 2019-08-28

**Authors:** Nima C. Emami, Linda Kachuri, Travis J. Meyers, Rajdeep Das, Joshua D. Hoffman, Thomas J. Hoffmann, Donglei Hu, Jun Shan, Felix Y. Feng, Elad Ziv, Stephen K. Van Den Eeden, John S. Witte

**Affiliations:** 1 Program in Biological and Medical Informatics, University of California San Francisco, San Francisco, CA 94158 USA; 2 Department of Epidemiology and Biostatistics, University of California San Francisco, San Francisco, CA 94158 USA; 3 Department of Urology, University of California San Francisco, San Francisco, CA 94158 USA; 4 Department of Radiation Oncology, Helen Diller Family Comprehensive Cancer Center, University of California San Francisco, San Francisco, CA 94115 USA; 5 Institute for Human Genetics, University of California San Francisco, San Francisco, CA 94143 USA; 6 Department of Medicine, University of California San Francisco, San Francisco, CA 94158 USA; 7 Helen Diller Family Comprehensive Cancer Center, University of California San Francisco, San Francisco, CA 94158 USA; 8 Division of Research, Kaiser Permanente, Northern California, Oakland, CA 94612 USA

**Keywords:** Cancer genomics, Gene expression, Prostate cancer, Prostate

Correction to: *Nature Communications* 10.1038/s41467-019-10808-7, published online 15 July 2019.

The original version of this Article omitted the following from the Acknowledgements ‘This research has been conducted using the UK Biobank Resource under Application Number 14105’. This has been amended in the HTML and PDF versions of the Article.

