# Supplementary material for: Association of imputed prostate cancer transcriptome with disease risk reveals novel mechanisms
Source: Nat Commun. 2019 Jul 15;10:3107. doi: 10.1038/s41467-019-10808-7 (PMC6629701; doi:10.1038/s41467-019-10808-7)
Supplement: Supplementary file 1 — Supplementary Information [file 41467_2019_10808_MOESM1_ESM.pdf]

Supplementary Information: Association of Imputed Prostate Cancer Transcriptome with Disease Risk Reveals Novel Mechanisms

Supplementary Table 1. Description of Populations and Datasets

Dataset	Source	Subjects*		Genomic Data		Notes
		Cases	Controls	Number of Measurements	Platform	
Discovery	UK Biobank	7,963	189,218	92,693,895 imputed genotypes	Microarray *Affymetrix Axiom*	Imputed using Haplotype Reference Consortium panel
Replication	Kaiser Permanente (GERA, ProHealth, CMHS)	6,653	30,121	48,406,222 imputed genotypes	Microarray *Affymetrix Axiom*	Imputed using 1000 Genomes Phase III panel

* Subjects restricted to unrelated, male individuals of European ancestry

Supplementary Table 2. Description of Trans-Ethnic Populations and Datasets

Dataset	Source	Ethnic Group	Subjects		Genomic Data	Notes
			Cases	Controls		
Trans-Ethnic Analysis	Kaiser Permanente (GERA, ProHealth, CMHS)	African American	662	1,700	48,406,222 imputed genotypes	Genotyped on Affymetrix Axiom Platform, Imputed using 1000 Genomes Phase III Panel
		Latino	523	3,060		
		East Asian	300	2,872		

Supplementary Table 3. Comparison of GTEx and Training Data Model Composition

<table>
<tr><th>Gene</th><th>Fraction of Variants in GTEx Models with LD $r^2 > 0.3$ with a Training Model Variant</th><th>Fraction of Variants in Training Models with LD $r^2 > 0.3$ with a GTEx Model Variant</th></tr>
<tr><td rowspan="3">ABR</td><td>0</td><td>0</td></tr>
<tr><td colspan="2">Training Model Variants:
rs35446675, rs62056505</td></tr>
<tr><td colspan="2">GTEx Model Variants:
rs9902221, rs35015626, rs3744734, rs11657732, rs3867591, rs4968118, rs561261, rs2088276, rs7210401, rs2257222, rs11653269, rs2586240, rs12942046, rs2885350</td></tr>
<tr><td rowspan="3">AGAP7</td><td>0.08108108</td><td>0.75</td></tr>
<tr><td colspan="2">Training Model Variants:
rs10708919, rs10824973, rs10824974, rs10826223, rs10993994, rs10994577, rs11003511, rs11284399, rs113007204, rs11327043, rs11548236, rs11592181, rs117156369, rs12146177, rs12770171, rs12777595, rs142470094, rs143254339, rs17178655, rs200598858, rs28671507, rs34726772, rs371975360, rs373320530, rs3849145, rs4935065, rs570896036, rs59363341, rs61847076, rs61847077, rs7070965, rs71502415, rs75933669, rs76874542, rs78937603, rs7905112, rs79995577</td></tr>
<tr><td colspan="2">GTEx Model Variants:
rs3793791, rs6537552, rs7071471, rs10825652, rs4630240, rs10740051, rs10761618, rs3001855</td></tr>
<tr><td rowspan="3">APOM</td><td>0.375</td><td>0.625</td></tr>
<tr><td colspan="2">Training Model Variants:
rs147926185, rs2736157, rs3115674, rs3130048, rs3134962, rs34573382, rs367364, rs371249751, rs3869111, rs4999718, rs527506034, rs572241903, rs67682613, rs67841474, rs72548020, rs76343304</td></tr>
<tr><td colspan="2">GTEx Model Variants:
rs2534679, rs2855816, rs2855804, rs2855812, rs2246618, rs2857598, rs3131383, rs3101018, rs3132445, rs3130484, rs3131379, rs3117574, rs3131378, rs3117575, rs3117577, rs3115672, rs497309, rs1270942, rs433061, rs1150753, rs1150752, rs502771, rs502055, rs9271720</td></tr>
<tr><td rowspan="3">GALNT3</td><td>1</td><td>0.2307692</td></tr>
<tr><td colspan="2">Training Model Variants:
rs112302955, rs11674422, rs11686403, rs12692777, rs13406280, rs13422985, rs13427924, rs13429321, rs13430211, rs144706611, rs1968293, rs2303393, rs2303394, rs35674334, rs3762552, rs41268661, rs5836051, rs62174818, rs62174820, rs67722214, rs71028502</td></tr>
<tr><td colspan="2">GTEx Model Variants:
rs16849949, rs11693434, rs1077617, rs1077618, rs16850090, rs1439994, rs7606577, rs6432808, rs11898238, rs699482, rs1895701, rs4667835, rs6721582, rs13017898, rs10930185, rs9677856, rs16851176, rs4667850, rs7564041, rs6432896, rs13032976, rs2031002, rs6716020, rs3748895, rs12474618, rs12622292</td></tr>
<tr><td rowspan="3">KCNQ1</td><td>0</td><td>0</td></tr>
<tr><td colspan="2">Training Model Variants:
rs11022821, rs11025755, rs11042962, rs12271773, rs12292286, rs142914590, rs199907984, rs2519184, rs35224423, rs3842727, rs3842753, rs4929968, rs59469120, rs61870826, rs689, rs739545, rs7480143, rs79788804</td></tr>
<tr><td colspan="2">GTEx Model Variants:
rs11603658, rs11603670, rs10837859, rs10768964, rs17245213, rs1489945, rs12222287, rs12224008, rs10743009, rs592373, rs6578974, rs7113485, rs2585, rs3802971, rs7941585, rs6578256, rs6578257, rs756919, rs9645649, rs2106467, rs11023485, rs7929804, rs9666537,</td></tr>
</table>

<table>
<tr><th>Gene</th><th>Fraction of Variants in
GTEx Models with LD $r^2 > 0.3$
with a Training Model Variant</th><th>Fraction of Variants in
Training Models with LD $r^2 > 0.3$
with a GTEx Model Variant</th></tr>
<tr><td></td><td colspan="2">rs231359, rs231358, rs231917, rs4255520, rs231872, rs1057128, rs163150, rs7480855, rs2237892, rs2283228, rs4930012, rs12786533, rs367035, rs413781, rs7124487, rs4758468, rs3741355, rs4565879, rs11025452, rs7106188, rs7951998, rs2412194, rs7124812, rs7105826, rs12270220, rs10767227, rs7926739, rs7926974, rs7127562, rs4910716, rs11604961, rs885413, rs908165</td></tr>
<tr><td rowspan="3">KLK15</td><td>0</td><td>0</td></tr>
<tr><td colspan="2">Training Model Variants:
rs2659124, rs266863, rs266878, rs78177998</td></tr>
<tr><td colspan="2">GTEx Model Variants:
rs919276</td></tr>
<tr><td rowspan="3">NCOA4</td><td>0.3947368</td><td>1</td></tr>
<tr><td colspan="2">Training Model Variants:
rs10993994, rs10761581, rs10797293, rs10821609, rs10857544, rs10994470, rs11005000, rs11005001, rs113007204, rs11593018, rs117007447, rs11815907, rs12770171, rs140400658, rs149459027, rs199737079, rs201553402, rs2574950, rs2574979, rs2611492, rs2813326, rs2843566, rs3032323, rs35931666, rs36081243, rs3829175, rs4245023, rs4267018, rs4390270, rs55848837, rs61847076, rs61847077, rs61858186, rs6537554, rs7073005, rs7350420, rs78937603, rs7905981, rs7911198</td></tr>
<tr><td colspan="2">GTEx Model Variants:
rs10993994, rs1271629, rs2611513, rs4581397, rs4512771, rs6481165, rs6481166, rs11594356</td></tr>
<tr><td rowspan="3">RPS2</td><td>0.4285714</td><td>0.5</td></tr>
<tr><td colspan="2">Training Model Variants:
rs397435, rs113892113, rs1141684, rs146974237, rs147400256, rs17135712, rs17135716, rs17135718, rs17135720, rs17808854, rs1860236, rs189020189, rs2286466, rs2294623, rs2745112, rs2815302, rs338786, rs35389185, rs3751884, rs397460, rs55949541, rs57669496, rs59576250, rs60599587, rs61136482, rs61652593, rs6366, rs66715980, rs71385705, rs7190034, rs7194876, rs72764895, rs757601, rs8050755, rs8052713, rs9921461</td></tr>
<tr><td colspan="2">GTEx Model Variants:
rs397435, rs8048165, rs761052, rs11861453, rs4347630, rs2941955, rs8050166, rs1040497, rs12599644, rs758335, rs35955001, rs35795901, rs36118556, rs35938501, rs17605, rs8460, rs12929367, rs34973300, rs2107321, rs3094778, rs3094772</td></tr>
<tr><td rowspan="3">TMPRSS2</td><td>0.3684211</td><td>0.1111111</td></tr>
<tr><td colspan="2">Training Model Variants:
rs11463654, rs115578024, rs117016829, rs146564124, rs1557370, rs28360562, rs2839413, rs34205539, rs34983238, rs4327307, rs55992492, rs56095453, rs60687893, rs73231906, rs79783160, rs79872290, rs9978557, rs9979885, rs9984523</td></tr>
<tr><td colspan="2">GTEx Model Variants:
rs9305698, rs2837640, rs2837647, rs4818162, rs17000237, rs8128946, rs2837819, rs7279115, rs2150429, rs9977370, rs2837985, rs12482462, rs2837997, rs13048056, rs6517673, rs8134378, rs8134657, rs2838068, rs3850709, rs2309105, rs8130571, rs3895614, rs2309107, rs426173, rs6586329, rs408956, rs7282354, rs4920133, rs365163, rs220156, rs7280694, rs220160, rs220171, rs225311, rs9984851, rs2839502</td></tr>
<tr><td rowspan="2">VPS53</td><td>0.07142857</td><td>0.4090909</td></tr>
<tr><td colspan="2">Training Model Variants:
rs10708313, rs111583421, rs111764301, rs112189013, rs114972153, rs11652688, rs1237094, rs12449775, rs12952217, rs140168243, rs145868182, rs16953156, rs17620240, rs183156235,</td></tr>
</table>

Gene	Fraction of Variants in GTEx Models with LD $r^2 > 0.3$ with a Training Model Variant	Fraction of Variants in Training Models with LD $r^2 > 0.3$ with a GTEx Model Variant
	rs2456274, rs2543778, rs2740358, rs28396482, rs330999, rs331000, rs34185510, rs34699431, rs370503694, rs410157, rs4280315, rs437948, rs4531806, rs502018, rs555854868, rs55842940, rs6598823, rs7405543, rs7406992, rs76404864, rs77032846, rs77114150, rs77432469, rs79547623, rs8069166, rs9907824, rs9910739, rs9916862	
	GTEx Model Variants:<br>rs11650789, rs11867532, rs9748016, rs12453108, rs11652657, rs12951098, rs7219151, rs741677, rs9907102, rs7214033, rs12450330, rs8069369, rs2279890, rs7218263, rs592294, rs910920, rs2004532, rs17693812, rs2289632, rs12942046, rs3892291, rs12949506	

* Variants in **bold** present in both GTEx and Training Models

Supplementary Table 4. Summary Statistics for Significant and Suggestive, Non-Replicating Genes

Gene	Discovery (UK Biobank)		Replication (KP)		Model R^2	Locus
	Beta (SE)	*P*-value	Beta (SE)	*P*-value		
SLC22A3	-0.38 (0.06)	$1.30*10^{-10}$	-0.06 (0.06)	0.37	0.219	6q25.3
CHMP2B	5.07 (0.83)	$9.63*10^{-10}$	0.70 (0.55)	0.20	0.142	3p11.2
SLC7A14	-2.62 (0.43)	$1.50*10^{-9}$	-0.68 (0.52)	0.19	0.027	3q26.2
CSNK2B	-33.5 (5.81)	$8.03*10^{-9}$	-9.11 (6.14)	0.14	0.035	6p21.33
FOXP4	-3.04 (0.54)	$1.75*10^{-8}$	-0.41 (0.67)	0.54	0.128	6p21.1
VGLL3	8.27 (1.54)	$7.25*10^{-8}$	-0.1 (0.72)	0.89	0.026	3p12.1
FLOT1	-4.48 (0.86)	$2.14*10^{-7}$	-0.06 (0.92)	0.95	0.128	6p21.33
SLC22A2	-0.19 (0.04)	$4.03*10^{-7}$	0.001 (0.04)	0.97	0.282	6q25.3
PSORS1C3	0.09 (0.02)	$4.21*10^{-7}$	0.01 (0.02)	0.54	0.596	6p21.33
CTBP2	0.94 (0.19)	$6.88*10^{-7}$	0.12 (0.22)	0.58	0.307	10q26.13
STMN3	-17.3 (3.59)	$1.38*10^{-6}$	-4.48 (2.65)	0.09	0.312	20q13.33
LOC100505495	0.69 (0.14)	$1.60*10^{-6}$	0.21 (0.16)	0.19	0.190	19q13.2
ABR	25.0 (5.39)	$3.65*10^{-6}$	3.78 (7.15)	0.60	0.047	17p13.3
KCNQ1	5.51 (1.2)	$4.06*10^{-6}$	-0.70 (0.49)	0.16	0.149	11p15.5
DCAF12	-4.36 (1.01)	$1.62*10^{-5}$	-0.43 (0.83)	0.60	0.110	9p13.3
HIST1H2BJ	1.22 (0.29)	$2.55*10^{-5}$	-0.08 (0.37)	0.82	0.059	6p22.1
LY6G5C	3.12 (0.75)	$3.23*10^{-5}$	-1.08 (0.72)	0.13	0.067	6p21.33
HAT1	3.68 (0.89)	$3.41*10^{-5}$	0.43 (0.86)	0.62	0.112	2q31.1
PPA2	1.74 (0.42)	$3.57*10^{-5}$	0.05 (0.32)	0.87	0.256	4q24

Supplementary Table 5. Summary Statistics Conditional upon Prostate Cancer Risk GWAS Variants

Gene	Discovery Beta (SE)	Discovery *P*-value	Conditional Beta (SE)	Conditional *P*-value	$P_{Replication}$ < 0.05 ?	Locus	Adjusted Variant(s)	Variant Position (hg19)
MSMB	-1.63 (0.12)	$2.97*10^{-41}$	0.00 (0.00)	1	Yes	10q11.22	rs10993994	chr10:51549496
NCOA4	0.75 (0.06)	$1.34*10^{-38}$	-0.90 (0.42)	0.028	Yes	10q11.22	rs10993994	chr10:51549496
HNF1B	2.03 (0.16)	$5.89*10^{-36}$	1.22 (0.27)	$7.75*10^{-6}$	Yes	17q12	rs7501939	chr17:36101156
							rs11649743	chr17:36074979
AGAP7	1.21 (0.12)	$2.05*10^{-24}$	0.07 (0.18)	0.687	Yes	10q11.22	rs10993994	chr10:51549496
POU5F1B	3.64 (0.44)	$8.40*10^{-17}$	0.14 (0.52)	0.79	Yes	8q24.21	rs6983267	chr8:128413305
							rs10086908	chr8:128011937
							rs10090154	chr8:128532137
							rs12543663	chr8:127924659
							rs12682344	chr8:128106784
							rs1487232	chr8:128005247
							rs16902094	chr8:128320346
							rs445114	chr8:128323181
							rs7463708	chr8:128104055
C19orf48	2.95 (0.39)	$2.46*10^{-14}$	1.25 (0.45)	0.00513	Yes	19q13.33	rs2735839	chr19:51364623
							rs266870	chr19:51351934
							rs17632542	chr19:51361757
KLK15	1.65 (0.23)	$1.26*10^{-12}$	0.85 (0.33)	0.011	Yes	19q13.33	rs2735839	chr19:51364623
							rs266870	chr19:51351934
							rs17632542	chr19:51361757
PCAT1	-1.28 (0.18)	$5.01*10^{-12}$	-0.31 (0.40)	0.440	Yes	8q24.21	rs6983267	chr8:128413305
							rs10086908	chr8:128011937
							rs10090154	chr8:128532137
							rs12543663	chr8:127924659
							rs12682344	chr8:128106784
							rs1487232	chr8:128005247
							rs16902094	chr8:128320346
							rs445114	chr8:128323181
							rs7463708	chr8:128104055
TMPRSS2	0.50 (0.08)	$2.42*10^{-9}$	0.45 (0.09)	$5.17*10^{-7}$	Yes	21q22.3	rs1041449	chr21:42901421
FAM57A	-0.50 (0.08)	$4.23*10^{-9}$	-0.29 (0.20)	0.135	Yes	17p13.3	rs684232	chr17:618965
PPP1R14A	1.80 (0.31)	$9.99*10^{-9}$	-0.44 (1.04)	0.670	Yes	19q13.2	rs8102476	chr19:38735613
							rs11672691	chr19:41985587
							rs61088131	chr19:42700947
ZFP36L2	-4.06 (0.74)	$4.26*10^{-8}$	-2.93 (0.93)	0.00165	Yes	2p21	rs1465618	chr2:43553949
BHLHA15	1.80 (0.33)	$5.18*10^{-8}$	-0.72 (0.71)	0.310	Yes	7q21.3	rs6465657	chr7:97816327
GEMIN4	-2.16 (0.41)	$1.39*10^{-7}$	1.78 (1.52)	0.243	Yes	17p13.3	rs684232	chr17:618965
STK25	4.97 (1.02)	$9.85*10^{-7}$	4.98 (1.02)	$9.37*10^{-7}$	Yes	2q37.3	rs2292884	chr2:238443226
KLK1	0.36 (0.08)	$7.71*10^{-6}$	0.13 (0.09)	0.145	Yes	19q13.33	rs2735839	chr19:51364623
							rs266870	chr19:51351934

Gene	Discovery Beta (SE)	Discovery *P*-value	Conditional Beta (SE)	Conditional *P*-value	$P_{Replication}$ < 0.05 ?	Locus	Adjusted Variant(s)	Variant Position (hg19)
							rs17632542	chr19:51361757
HOXA4	-5.71 (1.31)	$1.43*10^{-5}$	-2.67 (1.41)	0.059	Yes	7p15.2	rs10486567	chr7:27976563
VPS53	-2.3 (0.53)	$1.68*10^{-5}$	0.38 (0.86)	0.661	Yes	17p13.3	rs684232	chr17:618965
TIMM23	3.31 (0.79)	$2.77*10^{-5}$	1.24 (0.81)	0.122	Yes	10q11.22	rs10993994	chr10:51549496
SLC22A3	-0.38 (0.06)	$1.30*10^{-10}$	-0.21 (0.08)	0.010	No	6q25.3	rs651164*	chr6:160581374
							rs9364554	chr6:160833664
CHMP2B	5.07 (0.83)	$9.63*10^{-10}$	3.27 (1.26)	0.00954	No	3p11.2	rs17181170	chr3:87173324
SLC7A14	-2.62 (0.43)	$1.50*10^{-9}$	-1.29 (0.67)	0.053	No	3q26.2	rs71277158	chr3:169999216
							rs142436749	chr3:169093100
							rs10936632	chr3:170130102
CSNK2B	-33.5 (5.81)	$8.03*10^{-9}$	-29.6 (6.33)	$5.97*10^{-5}$	No	6p21.33	rs130067	chr6:31118511
							rs12665339	chr6:30601232
FOXP4	-3.04 (0.54)	$1.75*10^{-8}$	-1.21 (1.07)	0.259	No	6p21.1	rs1983891	chr6:41536427
VGLL3	8.27 (1.54)	$7.25*10^{-8}$	7.54 (2.38)	0.00151	No	3p12.1	rs2660753	chr3:87110674
FLOT1	-4.48 (0.86)	$2.14*10^{-7}$	-4.13 (0.87)	$1.09*10^{-5}$	No	6p21.33	rs130067	chr6:31118511
							rs12665339	chr6:30601232
SLC22A2	-0.19 (0.04)	$4.03*10^{-7}$	-0.08 (0.04)	0.057	No	6q25.3	rs651164*	chr6:160581374
							rs9364554	chr6:160833664
PSORS1C3	0.09 (0.02)	$4.21*10^{-7}$	0.08 (0.02)	0.00104	No	6p21.33	rs130067	chr6:31118511
							rs12665339	chr6:30601232
CTBP2	0.94 (0.19)	$6.88*10^{-7}$	0.92 (0.42)	0.029	No	10q26.13	rs4962416	chr10:126696872
STMN3	-17.3 (3.59)	$1.38*10^{-6}$	-15.6 (3.65)	$1.84*10^{-5}$	No	20q13.33	rs6062509	chr20:62362563
LOC100505495	0.69 (0.14)	$1.60*10^{-6}$	0.60 (0.20)	0.00296	No	19q13.2	rs8102476	chr19:38735613
							rs11672691	chr19:41985587
							rs61088131	chr19:42700947
ABR	25.0 (5.39)	$3.65*10^{-6}$	14.1 (5.95)	0.018	No	17p13.3	rs684232	chr17:618965
KCNQ1	5.51 (1.2)	$4.06*10^{-6}$	5.52 (1.2)	$3.96*10^{-6}$	No	11p15.5	rs7127900*	chr11:2233574
							rs1881502	chr11:1507512
DCAF12	-4.36 (1.01)	$1.62*10^{-5}$	-	-	No	9p13.3	rs10122495*	chr9:34049779
HIST1H2BJ	1.22 (0.29)	$2.55*10^{-5}$	1.17 (0.29)	$7.09*10^{-5}$	No	6p22.1	rs115457135	chr6:30073776
LY6G5C	3.12 (0.75)	$3.23*10^{-5}$	2.84 (0.76)	$1.39*10^{-4}$	No	6p21.33	rs130067	chr6:31118511
							rs12665339	chr6:30601232
HAT1	3.68 (0.89)	$3.41*10^{-5}$	3.59 (0.89)	$5.56*10^{-5}$	No	2q31.1	rs12621278	chr2:173311553
PPA2	1.74 (0.42)	$3.57*10^{-5}$	0.60 (0.45)	0.185	No	4q24	rs7679673	chr4:106061534
							rs11728350	ch4:106078097

* Lead/Proxy SNP (r^2 > 0.8 in 1000 Genomes Phase III EUR) not imputed for SLC22A2, SLC22A3, KCNQ1, DCAF12

Supplementary Table 6. Summary statistics from mutually adjusted models including all significant or suggestively associated genes in the same cytogenetic locus

Locus	Gene	Conditional Beta (SE)	Conditional *P*-value	Discovery *P*-value
6p21.33	CSNK2B	-40.15 (11.01)	$2.65*10^{-4}$	$8.03*10^{-9}$
	FLOT1	-2.57 (0.98)	0.00852	$2.14*10^{-7}$
	PSORS1C3	-0.035 (0.04)	0.333	$4.21*10^{-7}$
	LY6G5C	3.02 (0.79)	$1.24*10^{-4}$	$3.23*10^{-5}$
6q25.3	SLC22A2	0.15 (0.09)	0.091	$4.03*10^{-7}$
	SLC22A3	-0.59 (0.14)	$1.92*10^{-5}$	$1.30*10^{-10}$
8q24.21	POU5F1B	3.54 (0.44)	$5.89*10^{-16}$	$8.40*10^{-17}$
	PCAT1	-1.23 (0.18)	$3.18*10^{-11}$	$5.01*10^{-12}$
10q11.22	MSMB	-3.43 (0.87)	$8.48*10^{-5}$	$2.97*10^{-41}$
	NCOA4	-1.01 (0.43)	0.019	$1.34*10^{-38}$
	AGAP7	0.30 (0.19)	0.117	$2.05*10^{-24}$
	TIMM23	1.52 (0.87)	0.078	$2.77*10^{-5}$
17p13.3	FAM57A	-0.43 (0.21)	0.042	$4.23*10^{-9}$
	GEMIN4	0.27 (1.02)	0.794	$1.39*10^{-7}$
	ABR	12.88 (6.08)	0.034	$3.65*10^{-6}$
	VPS53	-0.19 (0.75)	0.795	$1.68*10^{-5}$
19q13.2	PPP1R14A	1.78 (0.31)	$1.24*10^{-8}$	$9.99*10^{-9}$
	LOC100505495	0.69 (0.14)	$1.99*10^{-6}$	$1.60*10^{-6}$
19q13.33	KLK1	0.06 (0.09)	0.482	$7.71*10^{-6}$
	KLK15	0.99 (0.27)	$2.32*10^{-4}$	$1.26*10^{-12}$
	C19orf48	2.05 (0.46)	$6.54*10^{-6}$	$2.46*10^{-14}$

Supplementary Table 7. Summary Statistics for Trans-Ethnic Replication Meta-Analysis

Gene	Transethnic Meta *P*-Value	Transethnic Meta Beta (SE)	AFR Beta (SE)	AFR *P*-value	LAT Beta (SE)	LAT *P*-value	EAS Beta (SE)	EAS *P*-value	Direction of Effect (AFR / LAT / EAS)	Discovery + Replication Meta Beta (SE)
MSMB	$1.63*10^{-4}$	-1.18 (0.31)	-0.37 (0.5)	0.45	-2.04 (0.52)	$7.74*10^{-5}$	-1.22 (0.66)	0.06	– / – / –	-1.57 (0.09)
NCOA4	$2.06*10^{-4}$	0.55 (0.15)	0.14 (0.24)	0.56	0.93 (0.24)	$8.74*10^{-5}$	0.56 (0.3)	0.06	+ / + / +	0.71 (0.04)
HNF1B	0.36	0.43 (0.46)	-0.25 (0.93)	0.79	0.48 (0.64)	0.46	1.03 (0.97)	0.29	– / + / +	1.91 (0.12)
AGAP7	0.11	0.39 (0.25)	-0.34 (0.38)	0.37	0.97 (0.4)	0.02	0.82 (0.54)	0.13	– / + / +	0.87 (0.08)
POU5F1B	0.22	1.52 (1.25)	2.49 (1.95)	0.20	1.58 (1.97)	0.42	-0.70 (2.88)	0.81	+ / + / –	3.55 (0.34)
C19orf48	0.35	0.95 (1.02)	3.18 (1.67)	0.06	-2.10 (1.48)	0.16	4.81 (2.54)	0.06	+ / – / +	2.51 (0.28)
KLK15	0.23	0.72 (0.6)	1.35 (0.95)	0.15	-0.72 (0.98)	0.46	2.03 (1.28)	0.11	+ / – / +	1.46 (0.18)
PCAT1	0.00568	-1.52 (0.55)	-2.95 (0.91)	0.0012	-0.08 (0.8)	0.92	-2.44 (1.34)	0.07	– / – / –	-1.33 (0.14)
TMPRSS2	0.65	-0.11 (0.24)	-0.02 (0.35)	0.94	0.003 (0.34)	0.99	-1.90 (1.06)	0.07	– / + / –	0.37 (0.06)
FAM57A	0.77	-0.07 (0.24)	-0.04 (0.48)	0.93	-0.26 (0.35)	0.47	0.20 (0.45)	0.65	– / – / +	-0.40 (0.06)
PPP1R14A	0.00461	2.41 (0.85)	3.16 (1.45)	0.03	2.23 (1.32)	0.09	1.64 (1.73)	0.34	+ / + / +	1.66 (0.24)
ZFP36L2	0.73	0.71 (2.01)	0.30 (3.94)	0.94	-3.87 (2.99)	0.20	8.22 (3.74)	0.03	+ / – / +	-3.78 (0.56)
BHLHA15	0.36	0.67 (0.74)	1.18 (1.3)	0.36	-0.08 (0.99)	0.94	2.56 (2.04)	0.21	+ / – / –	1.20 (0.21)
GEMIN4	0.68	-0.43 (1.07)	-0.31 (1.77)	0.86	-1.65 (1.68)	0.33	1.47 (2.21)	0.51	– / – / +	-1.86 (0.31)
STK25	0.08	4.36 (2.52)	1.00 (4.55)	0.83	5.23 (4.09)	0.20	6.61 (4.5)	0.14	– / – / –	4.39 (0.72)
KLK1	0.89	-0.02 (0.16)	0.21 (0.31)	0.50	-0.34 (0.24)	0.14	0.24 (0.28)	0.39	+ / – / +	0.33 (0.05)
HOXA4	0.27	-3.07 (2.81)	-8.13 (5.69)	0.15	-3.89 (3.53)	0.27	11.0 (7.96)	0.17	– / – / +	-3.18 (0.76)
VPS53	0.74	-0.32 (0.96)	0.71 (1.4)	0.62	-2.38 (1.81)	0.19	0.06 (1.92)	0.97	+ / – / +	-1.83 (0.37)
TIMM23	0.98	0.05 (2.29)	-5.69 (3.79)	0.13	5.45 (3.52)	0.12	-0.88 (5.04)	0.86	– / + / –	3.37 (0.6)

Supplementary Table 8. Association of 19 Imputed Genes with TCGA PRAD Subtypes

Imputed Gene	TCGA PRAD Subtypes (N_{total} = 286)					
	**ETV1 (N = 7)**	**ETV4 (N = 5)**	**SPOP (N = 31)**	**ERG (N = 109)**	**IDH1 (N = 2)**	**FOXA1 (N = 14)**
AGAP7	Beta (SE): 0.81 (2.2) *P* = 0.717	Beta (SE): 4.0 (2.6) *P* = 0.116	Beta (SE): -2.1 (1.3) *P* = 0.10	Beta (SE): 0.28 (0.74) *P* = 0.71	Beta (SE): -1.0 (4.5) *P* = 0.82	Beta (SE): 2.1 (1.6) *P* = 0.19
BHLHA15	Beta (SE): -0.6 (6.6) *P* = 0.93	Beta (SE): 0.65 (8.3) *P* = 0.94	Beta (SE): 5.4 (4.1) *P* = 0.18	Beta (SE): -0.02 (2.2) *P* = 0.99	Beta (SE): 9.5 (17.4) *P* = 0.59	Beta (SE): -1.4 (4.6) *P* = 0.76
TMPRSS2	Beta (SE): 0.66 (3.1) *P* = 0.83	Beta (SE): -3.1 (2.6) *P* = 0.24	Beta (SE): 0.13 (1.5) *P* = 0.93	Beta (SE): 0.75 (0.97) *P* = 0.44	Beta (SE): -0.67 (5.1) *P* = 0.90	Beta (SE): -1.2 (1.9) *P* = 0.53
ZFP36L2	Beta (SE): 3.4 (21.2) *P* = 0.87	Beta (SE): 61.6 (28.5) *P* = 0.03	Beta (SE): -8.4 (10.4) *P* = 0.42	Beta (SE): -3.5 (6.8) *P* = 0.60	Beta (SE): 41.0 (42.4) *P* = 0.33	Beta (SE): 3.7 (15.2) *P* = 0.81
C19orf48	Beta (SE): -2.5 (7.5) *P* = 0.74	Beta (SE): 0.55 (8.8) *P* = 0.95	Beta (SE): 6.5 (3.8) *P* = 0.09	Beta (SE): -0.3 (2.4) *P* = 0.89	Beta (SE): -25.3 (16.0) *P* = 0.11	Beta (SE): -2.0 (5.3) *P* = 0.71
FAM57A	Beta (SE): 8.8 (3.7) *P* = 0.02	Beta (SE): 2.7 (3.6) *P* = 0.44	Beta (SE): -0.10 (1.5) *P* = 0.95	Beta (SE): 0.14 (0.95) *P* = 0.89	Beta (SE): 0.77 (5.5) *P* = 0.89	Beta (SE): 2.6 (2.2) *P* = 0.23
GEMIN4	Beta (SE): 49.9 (20.1) *P* = 0.01	Beta (SE): 4.2 (15.5) *P* = 0.78	Beta (SE): 1.7 (6.5) *P* = 0.79	Beta (SE): 1.7 (4.2) *P* = 0.69	Beta (SE): -3.5 (24.1) *P* = 0.89	Beta (SE): 4.7 (9.4) *P* = 0.62
HNF1B	Beta (SE): 1.4 (5.6) *P* = 0.80	Beta (SE): -5.0 (6.9) *P* = 0.47	Beta (SE): 3.1 (2.8) *P* = 0.27	Beta (SE): -2.9 (1.8) *P* = 0.11	Beta (SE): -0.61 (10.4) *P* = 0.95	Beta (SE): 9.4 (4.3) *P* = 0.03
HOXA4	Beta (SE): -21.4 (20.2) *P* = 0.29	Beta (SE): -18.1 (24.8) *P* = 0.47	Beta (SE): -8.8 (12.3) *P* = 0.48	Beta (SE): -0.17 (8.7) *P* = 0.98	Beta (SE): -4.1 (47.6) *P* = 0.93	Beta (SE): 15.8 (23.1) *P* = 0.50

Imputed Gene	TCGA PRAD Subtypes (N_{total} = 286)					
	**ETV1 (N = 7)**	**ETV4 (N = 5)**	**SPOP (N = 31)**	**ERG (N = 109)**	**IDH1 (N = 2)**	**FOXA1 (N = 14)**
KLK1	Beta (SE): -4.1 (3.0) *P* = 0.17	Beta (SE): 0.71 (2.7) *P* = 0.80	Beta (SE): 0.09 (1.2) *P* = 0.94	Beta (SE): 0.81 (0.77) *P* = 0.29	Beta (SE): -21.9 (13.4) *P* = 0.10	Beta (SE): -0.21 (1.73) *P* = 0.91
KLK15	Beta (SE): -3.3 (5.9) *P* = 0.58	Beta (SE): 15.1 (10.3) *P* = 0.14	Beta (SE): 4.3 (3.2) *P* = 0.19	Beta (SE): 0.58 (2.0) *P* = 0.77	Beta (SE): 11.4 (14.6) *P* = 0.43	Beta (SE): -1.46 (4.3) *P* = 0.73
MSMB	Beta (SE): -5.9 (4.3) *P* = 0.17	Beta (SE): -5.3 (5.0) *P* = 0.29	Beta (SE): 1.1 (2.0) *P* = 0.58	Beta (SE): 1.0 (1.3) *P* = 0.43	Beta (SE): -6.6 (8.1) *P* = 0.42	Beta (SE): -0.75 (2.9) *P* = 0.80
NCOA4	Beta (SE): 1.4 (1.3) *P* = 0.30	Beta (SE): 0.8 (1.7) *P* = 0.61	Beta (SE): 0.11 (0.77) *P* = 0.89	Beta (SE): -0.88 (0.52) *P* = 0.09	Beta (SE): 3.7 (1.8) *P* = 0.04	Beta (SE): -0.20 (1.1) *P* = 0.86
PCAT1	Beta (SE): 6.0 (4.6) *P* = 0.19	Beta (SE): 5.9 (5.4) *P* = 0.27	Beta (SE): -3.6 (2.8) *P* = 0.19	Beta (SE): 1.6 (1.6) *P* = 0.34	Beta (SE): 1.5 (9.0) *P* = 0.87	Beta (SE): 2.5 (3.4) *P* = 0.47
POU5F1B	Beta (SE): -14.3 (18.3) *P* = 0.43	Beta (SE): -24.8 (25.4) *P* = 0.33	Beta (SE): -1.0 (7.6) *P* = 0.89	Beta (SE): -5.2 (5.0) *P* = 0.30	Beta (SE): 29.4 (23.8) *P* = 0.22	Beta (SE): 2.6 (10.6) *P* = 0.81
PPP1R14A	Beta (SE): 5.6 (2.8) *P* = 0.04	Beta (SE): 0.95 (2.6) *P* = 0.72	Beta (SE): -1.8 (1.1) *P* = 0.11	Beta (SE): 1.6 (0.73) *P* = 0.02	Beta (SE): 0.46 (4.1) *P* = 0.91	Beta (SE): -1.5 (1.6) *P* = 0.36
STK25	Beta (SE): 28.7 (34.8) *P* = 0.41	Beta (SE): 17.1 (38.6) *P* = 0.658	Beta (SE): 19.3 (16.2) *P* = 0.235	Beta (SE): 4.2 (9.8) *P* = 0.667478	Beta (SE): 127.1 (116.9) *P* = 0.277	Beta (SE): -4.1 (21.3) *P* = 0.847
TIMM23	Beta (SE): 17.2 (22.1) *P* = 0.44	Beta (SE): -7.4 (28.7) *P* = 0.80	Beta (SE): -5.5 (12.0) *P* = 0.65	Beta (SE): -5.3 (7.6) *P* = 0.49	Beta (SE): -20.3 (48.1) *P* = 0.67	Beta (SE): -10.2 (17.6) *P* = 0.57
VPS53	Beta (SE): 6.2 (10.8) *P* = 0.57	Beta (SE): 16.3 (12.1) *P* = 0.18	Beta (SE): 2.2 (5.5) *P* = 0.69	Beta (SE): 1.7 (3.6) *P* = 0.63	Beta (SE): -20.6 (22.1) *P* = 0.35	Beta (SE): 5.2 (7.8) *P* = 0.51

Supplementary Table 9. 100 Randomly Selected Genes Tested For eQTLs in or tagging VCaP ChIP-Seq Transcription Factor Binding Sites

Tested Genes: AGMAT, ANXA3, APBA2, APBB3, APOB, APOBEC3A, B4GALT7, BPIFB2, C13orf15, C6orf164, C6orf72, CASD1, CDA, CDC40, CRIPAK, CXCL17, CYB561, DBNDD1, DDX5, DENND5A, DPM2, DTNB, EEA1, ENTPD3-AS1, FAM43B, FAM70B, FAM78B, FAM82A1, FCER1G, FLJ42627, FOXA3, FREM1, GLIPR2, HPS4, KCNE2, KCNJ11, KREMEN2, LHX6, LOC100507218, LOC391322, LOC440925, LRIG1, LRRC20, MAGI1, MED25, MED28, MFSD6L, MKS1, MORC2-AS1, MPO, MTX2, NBN, NBPF7, NDRG1, NENF, NTNG1, NUDCD1, NUDT13, NUDT7, OTOGL, P2RY2, PAQR8, PDCD11, PEX6, PGBD1, PIWIL2, PLA2G7, PLEKHM2, PPIL2, PRKACA, PTPLB, PTPRM, PURA, RANBP2, RPL23AP53, RPN1, RSRC1, RTP4, SEC14L1, SEMA4G, SH3TC2, SLC2A6, SLC6A1, SPATS2L, SZT2, TBRG4, TGOLN2, THPO, TIMP2, TMEM200C, TRIM61, TRIM9, TXNDC16, UBL7, UBP1, UPK3BL, WDR77, ZNF100, ZNF845, ZNF846

Gene	VCaP ChIP-Seq TFBS	Variant(s) (hg19 position)
APOB	AR	rs10187591 (chr2:21675836)
B4GALT7	AR	rs189174826 (chr5:177426027)
	SPDEF	rs2545799 (chr5:176852185)
C6orf164	AR	rs34019278 (chr6:87986767), rs34107692 (chr6:88268343), rs4707351 (chr6:87929303)
	ERG	rs12660483 (chr6:88250619), rs12663400 (chr6:88250588), rs16879532 (chr6:88250389), rs62417675 (chr6:88250739)
C6orf72	AR	rs10872650 (chr6:150082094), rs75507681 (chr6:150082412)
CASD1	AR	rs13234526 (chr7:94136930)
DDX5	AR	rs11396994 (chr17:62704919)
DENND5A	AR	rs35091868 (chr11:9120639)
DPM2	AR	rs2244331 (chr9:130216119)
DTNB	AR	rs7591899 (chr2:25389158)

Tested Genes: AGMAT, ANXA3, APBA2, APBB3, APOB, APOBEC3A, B4GALT7, BPIFB2, C13orf15, C6orf164, C6orf72, CASD1, CDA, CDC40, CRIPAK, CXCL17, CYB561, DBNDD1, DDX5, DENND5A, DPM2, DTNB, EEA1, ENTPD3-AS1, FAM43B, FAM70B, FAM78B, FAM82A1, FCER1G, FLJ42627, FOXA3, FREM1, GLIPR2, HPS4, KCNE2, KCNJ11, KREMEN2, LHX6, LOC100507218, LOC391322, LOC440925, LRIG1, LRRC20, MAGI1, MED25, MED28, MFSD6L, MKS1, MORC2-AS1, MPO, MTX2, NBN, NBPF7, NDRG1, NENF, NTNG1, NUDCD1, NUDT13, NUDT7, OTOGL, P2RY2, PAQR8, PDCD11, PEX6, PGBD1, PIWIL2, PLA2G7, PLEKHM2, PPIL2, PRKACA, PTPLB, PTPRM, PURA, RANBP2, RPL23AP53, RPN1, RSRC1, RTP4, SEC14L1, SEMA4G, SH3TC2, SLC2A6, SLC6A1, SPATS2L, SZT2, TBRG4, TGOLN2, THPO, TIMP2, TMEM200C, TRIM61, TRIM9, TXNDC16, UBL7, UBP1, UPK3BL, WDR77, ZNF100, ZNF845, ZNF846

Gene	VCaP ChIP-Seq TFBS	Variant(s) (hg19 position)
FAM82A1	AR	rs114704307 (chr2:37886656), rs13027679 (chr2:38051088), rs4670210 (chr2:37886900), rs4670211 (chr2:37887017), rs4670748 (chr2:37886777), rs56809883 (chr2:37886494), rs6759224 (chr2:37887089)
GLIPR2	SPDEF	rs2002096* (chr9:36317062), rs2002097* (chr9:36317122), rs2002098* (chr9:36317142)
KCNE2	AR	rs35056247* (chr21:36100726)
LHX6	AR	rs10818657 (chr9:125010884), rs10818659 (chr9:125011196), rs10985577 (chr9:125010922)
LHX6	ERG	rs10818657 (chr9:125010884), rs10818659 (chr9:125011196), rs10985577 (chr9:125010922)
LOC391322	AR	rs62233992* (chr22:24798636)
	SPDEF	rs5760299 (chr22:24666834)
LOC440925	AR	rs10198822 (chr2:171536324)
MAGI1	AR	rs10510945 (chr3:65817202), rs17073922 (chr3:65817182), rs2291243 (chr3:65093125)
NDRG1	AR	rs10108529 (chr8:134339616), rs111444688 (chr8:134339324), rs2976550 (chr8:134368713), rs58908089 (chr8:134338957)
NENF	AR	rs61828532 (chr1:212178262), rs6678627* (chr1:212363071)
	ERG	rs6678627* (chr1:212363071)
NUDCD1	SPDEF	rs74942793 (chr8:110574281)
NUDT13	AR	rs12250907 (chr10:75023247), rs148172640 (chr10:75336306), rs1880701 (chr10:75313231), rs74318005 (chr10:75336209), rs76487112 (chr10:75336453), rs77602688 (chr10:75336475)

**Tested Genes:** AGMAT, ANXA3, APBA2, APBB3, APOB, APOBEC3A, B4GALT7, BPIFB2, C13orf15, C6orf164, C6orf72, CASD1, CDA, CDC40, CRIPAK, CXCL17, CYB561, DBNDD1, DDX5, DENND5A, DPM2, DTNB, EEA1, ENTPD3-AS1, FAM43B, FAM70B, FAM78B, FAM82A1, FCER1G, FLJ42627, FOXA3, FREM1, GLIPR2, HPS4, KCNE2, KCNJ11, KREMEN2, LHX6, LOC100507218, LOC391322, LOC440925, LRIG1, LRRC20, MAGI1, MED25, MED28, MFSD6L, MKS1, MORC2-AS1, MPO, MTX2, NBN, NBPF7, NDRG1, NENF, NTNG1, NUDCD1, NUDT13, NUDT7, OTOGL, P2RY2, PAQR8, PDCD11, PEX6, PGBD1, PIWIL2, PLA2G7, PLEKHM2, PPIL2, PRKACA, PTPLB, PTPRM, PURA, RANBP2, RPL23AP53, RPN1, RSRC1, RTP4, SEC14L1, SEMA4G, SH3TC2, SLC2A6, SLC6A1, SPATS2L, SZT2, TBRG4, TGOLN2, THPO, TIMP2, TMEM200C, TRIM61, TRIM9, TXNDC16, UBL7, UBP1, UPK3BL, WDR77, ZNF100, ZNF845, ZNF846		
**Gene**	**VCaP ChIP-Seq TFBS**	**Variant(s) (hg19 position)**
OTOGL	AR	rs10862045 (chr12:80475448), rs17007452 (chr12:81243404)
PGBD1	AR	rs149963 (chr6:28017131), rs9368562 (chr6:28174401)
	SPDEF	rs9368544 (chr6:27986528), rs9393870 (chr6:27986882)
PLA2G7	AR	rs73736658 (chr6:46789576)
PTPRM	AR	rs12456630 (chr18:8337539), rs595649* (chr18:8341609), rs607396* (chr18:7755225), rs627141* (chr18:8342476), rs627146 (chr18:8342477), rs657374* (chr18:8380472), rs7505095 (chr18:7635884)
SEC14L1	AR	rs117380308 (chr17:75093140), rs117812440* (chr17:75093216)
SH3TC2	SPDEF	rs77063086* (chr5:148941570)
TRIM61	ERG	rs78760223* (chr4:166115453)
TXNDC16	AR	rs17125551 (chr14:53012546)
UBP1	AR	rs34444514 (chr3:33678933)
ZNF845	AR	rs34092966 (chr19:53867321), rs59716235* (chr19:54125278)

* Directly modeled eQTL variants in VCaP ChIP-Seq TFBS. Remaining variants in LD ($r^2 \geq 0.8$ in 1000 Genomes Phase III EUR) with a modeled eQTL variant.

Supplementary Table 10. Comparison of Directions of Effect on Androgen Receptor (AR) Binding Affinity (using sTRAP Transcription Factor Affinity Prediction) and Target Gene Expression Levels, either directly or by Modeled LD Proxy eQTL, for Variants in VCaP AR ChIP-Seq Peaks

**Androgen Receptor (AR) TFBS Variant**	**Variant Effect on AR Binding Affinity**			**Variant (or LD Proxy) Effect on Gene Expression**	
	**TRANSFAC Vertebrate 2010.1 Matrix**	**Effect of Allele A1 on sTRAP AR Binding Affinity: $\log_{10}(P_{Binding\_A1}) - \log_{10}(P_{Binding\_A2})$ [Rank]**	**Rank-Weighted Average of Allele A1 Effect on Binding Affinity**	**Modeled eQTL Variant A1 ↔ mA1 A2 ↔ mA2**	**Effect of mA1 Allele on Target Gene Expression**
rs58186870 Allele A1: A Allele A2: G	V$AR_01	0.750 [6]	0.552 *Increase* in AR Binding	rs142470094 (Proxy, 1000G EUR LD r^2 = 0.801)	β_{eQTL} = 0.016 (AGAP7) *Increase* in Expression
	V$AR_04	0.693 [8]			
	V$AR_03	0.006 [372]		Modeled Allele mA1: A	
	V$AR_02	0.002 [473]		Modeled Allele mA2: ATG	
	V$AR_Q6	-0.001 [506]		Target Gene(s): AGAP7	
	V$AR_Q2	$-8.48*10^{-5}$ [683]			
rs58677292 Allele A1: T Allele A2: C	V$AR_01	1.41 [1]	3.04 *Increase* in AR Binding	rs142470094 (Proxy, 1000G EUR LD r^2 = 0.801)	β_{eQTL} = 0.016 (AGAP7) *Increase* in Expression
	V$AR_04	1.29 [2]			
	V$AR_02	1.07 [4]		Modeled Allele mA1: A	
	V$AR_03	0.630 [15]		Modeled Allele mA2: ATG	
	V$AR_Q2	0.159 [89]		Target Gene(s): AGAP7	
	V$AR_Q6	$-4.00*10^{-5}$ [714]			
rs56106241 Allele A1: T Allele A2: G	V$AR_02	0.928 [9]	0.598 *Increase* in AR Binding	rs142470094 (Proxy, 1000G EUR LD r^2 = 0.809)	β_{eQTL} = 0.016 (AGAP7) *Increase* in Expression
	V$AR_Q6	0.717 [16]			
	V$AR_03	0.376 [54]		Modeled Allele mA1: A	
	V$AR_01	0.251 [90]		Modeled Allele mA2: ATG	
	V$AR_Q2	0.240 [93]		Target Gene(s): AGAP7	
	V$AR_04	0.096 [185]			
rs6975156 Allele A1: T Allele A2: C	V$AR_Q6	0.072 [182]	0.012 *Increase* in AR Binding	rs7790229 (Proxy, 1000G EUR LD r^2 = 0.988)	β_{eQTL} = 3.66E-04 (BHLHA15) *Increase* in Expression
	V$AR_03	0.051 [232]			
	V$AR_Q2	0.030 [308]		Modeled Allele mA1: G	
	V$AR_02	0.021 [362]		Modeled Allele mA2: A	
	V$AR_01	0.015 [412]		Target Gene(s): BHLHA15	
	V$AR_04	0.005 [569]			
rs7789380 Allele A1: G Allele A2: A	V$AR_01	0.108 [103]	0.014 *Increase* in AR Binding	rs7790229 (Proxy, 1000G EUR LD r^2 = 0.968)	β_{eQTL} = 3.65E-04 (BHLHA15) *Increase* in Expression
	V$AR_04	0.054 [162]			

Androgen Receptor (AR) TFBS Variant	Variant Effect on AR Binding Affinity			Variant (or LD Proxy) Effect on Gene Expression	
	**TRANSFAC Vertebrate 2010.1 Matrix**	**Effect of Allele A1 on sTRAP AR Binding Affinity: $\log_{10}(P_{Binding\_A1}) - \log_{10}(P_{Binding\_A2})$ [Rank]**	**Rank-Weighted Average of Allele A1 Effect on Binding Affinity**	**Modeled eQTL Variant A1 ↔ mA1 A2 ↔ mA2**	**Effect of mA1 Allele on Target Gene Expression**
	V$AR_03	-0.012 [304]		Modeled Allele mA1: G	
	V$AR_Q6	-0.004 [393]		Modeled Allele mA2: A	
	V$AR_02	0.003 [417]		Target Gene(s): BHLHA15	
	V$AR_Q2	$-5.20*10^{-4}$ [546]			
rs10953245 Allele A1: A Allele A2: C	V$AR_Q6	0.092 [116]	0.012 *Increase* in AR Binding	rs35527492 (Proxy, 1000G EUR LD r^2 = 0.996)	β_{eQTL} = -0.015 (BHLHA15) *Decrease* in Expression
	V$AR_03	0.027 [267]			
	V$AR_02	0.017 [329]		Modeled Allele mA1: T	
	V$AR_Q2	0.006 [466]		Modeled Allele mA2: C	
	V$AR_04	0.002 [575]		Target Gene(s): BHLHA15	
	V$AR_01	0.002 [593]			
rs11665748 Allele A1: A Allele A2: G	V$AR_Q2	0.20 [83]	0.030 *Increase* in AR Binding	rs11665748 (Self, No Proxy)	β_{eQTL} = 0.004 (C19orf48) *Increase* in Expression
	V$AR_03	0.082 [165]			
	V$AR_01	0.061 [188]		Modeled Allele mA1: A	
	V$AR_02	-0.050 [209]		Modeled Allele mA2: G	
	V$AR_04	0.045 [220]		Target Gene(s): C19orf48	
	V$AR_Q6	-0.039 [236]			
rs78177998 Allele A1: T Allele A2: C	V$AR_Q2	0.142 [108]	0.035 *Increase* in AR Binding	rs78177998 (Self, No Proxy)	β_{eQTL} = 0.017 (C19orf48) β_{eQTL} = 0.021 (KLK15) Mean *Increase* in Expression
	V$AR_Q6	0.135 [112]			
	V$AR_04	0.082 [161]		Modeled Allele mA1: T	
	V$AR_03	0.022 [346]		Modeled Allele mA2: C	
	V$AR_01	0.015 [391]		Target Gene(s): C19orf48, KLK15	
	V$AR_02	0.008 [463]			
rs2659051 Allele A1: G Allele A2: C	V$AR_Q6	0.019 [302]	0.003 *Increase* in AR Binding	rs2659051 (Self, No Proxy)	β_{eQTL} = 0.003 (C19orf48) *Increase* in Expression
	V$AR_04	0.013 [350]			
	V$AR_03	0.011 [365]		Modeled Allele mA1: G	
	V$AR_01	0.005 [439]		Modeled Allele mA2: C	
	V$AR_02	0.004 [456]		Target Gene(s): C19orf48	
	V$AR_Q2	$7.64*10^{-4}$ [579]			
rs11665698 Allele A1: C Allele A2: A	V$AR_01	0.219 [171]	0.026 *Increase* in AR Binding	rs11665748 (Proxy, 1000G EUR LD r^2 = 0.959)	β_{eQTL} = 0.004 (C19orf48) *Increase* in Expression
	V$AR_04	0.167 [210]			

Androgen Receptor (AR) TFBS Variant	Variant Effect on AR Binding Affinity			Variant (or LD Proxy) Effect on Gene Expression	
	**TRANSFAC Vertebrate 2010.1 Matrix**	**Effect of Allele A1 on sTRAP AR Binding Affinity: $\log_{10}(P_{Binding\_A1}) - \log_{10}(P_{Binding\_A2})$ [Rank]**	**Rank-Weighted Average of Allele A1 Effect on Binding Affinity**	**Modeled eQTL Variant A1 ↔ mA1 A2 ↔ mA2**	**Effect of mA1 Allele on Target Gene Expression**
	V$AR_Q6	-0.083 [293]		Modeled Allele mA1: A	
	V$AR_Q2	0.051 [335]		Modeled Allele mA2: G	
	V$AR_03	0.002 [566]		Target Gene(s): C19orf48	
	V$AR_02	-7.08×10^{-6} [835]			
rs461251 Allele A1: A Allele A2: G	V$AR_02	0.207 [82]	0.010 *Increase* in AR Binding	rs461251 (Self, No Proxy)	β_{eQTL} = 0.035 (FAM57A) β_{eQTL} = 0.016 (GEMIN4) β_{eQTL} = 0.035 (VPS53) Mean *Increase* in Expression
	V$AR_01	-0.137 [125]			
	V$AR_Q2	-0.101 [163]		Modeled Allele mA1: A	
	V$AR_04	0.060 [221]		Modeled Allele mA2: G	
	V$AR_03	0.049 [241]		Target Gene(s): FAM57A, GEMIN4, VPS53	
	V$AR_Q6	0.005 [486]			
rs684232 Allele A1: T Allele A2: C	V$AR_Q2	0.081 [171]	0.004 *Increase* in AR Binding	rs684232 (Self, No Proxy)	β_{eQTL} = 0.017 (FAM57A) β_{eQTL} = 0.002 (GEMIN4) β_{eQTL} = -0.003 (VPS53) Mean *Increase* in Expression
	V$AR_02	-0.017 [315]			
	V$AR_04	-0.014 [347]		Modeled Allele mA1: T	
	V$AR_03	-0.012 [357]		Modeled Allele mA2: C	
	V$AR_01	-0.002 [515]		Target Gene(s): FAM57A, GEMIN4, VPS53	
	V$AR_Q6	-7.11×10^{-4} [617]			
rs11084033 Allele A1: A Allele A2: C	V$AR_Q6	0.181 [72]	0.014 *Increase* in AR Binding	rs11084033 (Self, No Proxy)	β_{eQTL} = 0.059 (KLK1) *Increase* in Expression
	V$AR_02	-0.052 [177]			
	V$AR_04	-0.035 [224]		Modeled Allele mA1: A	
	V$AR_01	-0.022 [282]		Modeled Allele mA2: C	
	V$AR_Q2	4.21 [655]		Target Gene(s): KLK1	
	V$AR_03	-9.35×10^{-5} [755]			
rs12571566 Allele A1: T Allele A2: C	V$AR_03	0.458 [23]	0.056 *Increase* in AR Binding	rs10797293 (Proxy, 1000G EUR LD r^2 = 0.863)	β_{eQTL} = -0.005 (NCOA4) *Decrease* in Expression
	V$AR_Q6	-0.295 [47]			
	V$AR_02	0.052 [236]		Modeled Allele mA1: G	
	V$AR_Q2	0.038 [262]		Modeled Allele mA2: C	
	V$AR_04	-0.038 [264]		Target Gene(s): NCOA4	
	V$AR_01	-4.11×10^{-3} [440]			
rs61848292 Allele A1: A Allele A2: G	V$AR_03	0.160 [81]	0.026 *Increase* in AR Binding	rs10797293 (Proxy, 1000G EUR LD r^2 = 0.876)	β_{eQTL} = 0.005 (NCOA4) *Increase* in Expression
	V$AR_02	0.093 [129]			

**Androgen Receptor (AR) TFBS Variant**	**Variant Effect on AR Binding Affinity**			**Variant (or LD Proxy) Effect on Gene Expression**	
	**TRANSFAC Vertebrate 2010.1 Matrix**	**Effect of Allele A1 on sTRAP AR Binding Affinity: $\log_{10}(P_{Binding\_A1}) - \log_{10}(P_{Binding\_A2})$ [Rank]**	**Rank-Weighted Average of Allele A1 Effect on Binding Affinity**	**Modeled eQTL Variant A1 ↔ mA1 A2 ↔ mA2**	**Effect of mA1 Allele on Target Gene Expression**
	V$AR_Q2	0.005 [434]		Modeled Allele mA1: C	
	V$AR_Q6	$5.67*10^{-4}$ [632]		Modeled Allele mA2: G	
	V$AR_04	$3.17*10^{-6}$ [856]		Target Gene(s): NCOA4	
	V$AR_01	0 [904]			
rs12569965 Allele A1: G Allele A2: T	V$AR_Q6	0.124 [168]	0.010 *Increase* in AR Binding	rs10797293 (Proxy, 1000G EUR LD r^2 = 0.863)	β_{eQTL} = -0.005 (NCOA4) *Decrease* in Expression
	V$AR_Q2	0.014 [407]			
	V$AR_02	-0.010 [434]		Modeled Allele mA1: G	
	V$AR_01	$6.42*10^{-4}$ [642]		Modeled Allele mA2: C	
	V$AR_03	$-1.80*10^{-4}$ [699]		Target Gene(s): NCOA4	
	V$AR_04	$2.90*10^{-5}$ [790]			
rs73034946 Allele A1: T Allele A2: C	V$AR_03	0.189 [94]	0.024 *Increase* in AR Binding	rs241955 (Proxy, 1000G EUR LD r^2 = 0.921)	β_{eQTL} = 0.018 (PPP1R14A) *Increase* in Expression
	V$AR_Q6	0.043 [273]			
	V$AR_Q2	0.017 [375]		Modeled Allele mA1: A	
	V$AR_02	0.016 [379]		Modeled Allele mA2: G	
	V$AR_01	$1.19*10^{-5}$ [823]		Target Gene(s): PPP1R14A	
	V$AR_04	$3.67*10^{-7}$ [880]			
rs56390510 Allele A1: C Allele A2: G	V$AR_02	0.082 [133]	0.003 *Increase* in AR Binding	rs56390510 (Self, No Proxy)	β_{eQTL} = -0.001 (STK25) *Decrease* in Expression
	V$AR_03	-0.065 [157]			
	V$AR_01	0.018 [254]		Modeled Allele mA1: C	
	V$AR_04	0.009 [323]		Modeled Allele mA2: G	
	V$AR_Q2	-0.006 [364]		Target Gene(s): STK25	
	V$AR_Q6	$8.93*10^{-4}$ [514]			
rs56095453 Allele A1: A Allele A2: T	V$AR_03	-0.029 [313]	$9.17*10^{-4}$ *Increase* in AR Binding	rs56095453 (Self, No Proxy)	β_{eQTL} = 9.08E-04 (TMPRSS2) *Increase* in Expression
	V$AR_02	0.027 [322]			
	V$AR_01	0.007 [414]		Modeled Allele mA1: A	
	V$AR_Q2	0.005 [436]		Modeled Allele mA2: T	
	V$AR_Q6	0.005 [437]		Target Gene(s): TMPRSS2	
	V$AR_04	0.004 [462]			
rs8134378 Allele A1: G Allele A2: A	V$AR_01	1.50 [1]	2.80 *Increase* in AR Binding	rs9979885 (Proxy, 1000G EUR LD r^2 = 1.0)	β_{eQTL} = 0.019 (TMPRSS2) *Increase* in Expression
	V$AR_04	1.41 [3]			

Androgen Receptor (AR) TFBS Variant	Variant Effect on AR Binding Affinity			Variant (or LD Proxy) Effect on Gene Expression	
	**TRANSFAC Vertebrate 2010.1 Matrix**	**Effect of Allele A1 on sTRAP AR Binding Affinity: $\log_{10}(P_{Binding\_A1}) - \log_{10}(P_{Binding\_A2})$ [Rank]**	**Rank-Weighted Average of Allele A1 Effect on Binding Affinity**	**Modeled eQTL Variant A1 ↔ mA1 A2 ↔ mA2**	**Effect of mA1 Allele on Target Gene Expression**
	V$AR_Q2	0.838 [12]		Modeled Allele mA1: C	
	V$AR_02	0.727 [16]		Modeled Allele mA2: T	
	V$AR_03	0.380 [40]		Target Gene(s): TMPRSS2	
	V$AR_Q6	0.028 [310]			
rs8134657 Allele A1: G Allele A2: A	V$AR_02	0.076 [181]	0.008 *Increase* in AR Binding	rs9979885 (Proxy, 1000G EUR LD r^2 = 1.0)	β_{eQTL} = 0.019 (TMPRSS2) *Increase* in Expression
	V$AR_01	0.018 [334]			
	V$AR_Q6	0.017 [342]		Modeled Allele mA1: C	
	V$AR_04	0.003 [485]		Modeled Allele mA2: T	
	V$AR_Q2	0.002 [521]		Target Gene(s): TMPRSS2	
	V$AR_03	$-3.93*10^{-5}$ [786]			

Supplementary Table 11. Nominally Replicated Genes with eQTLs in or tagging LNCaP DNAse-Seq Transcription Factor Footprints

Gene	LNCaP Footprinting TFBS [Variant(s) (hg19 position)]
AGAP7	FOXM1 [rs113815588 (chr10:51506033)], TAL1::TCF3 [rs11599004 (chr10:51488377)], TBX15 [rs11599004 (chr10:51488377)], Tal-1beta:ITF-2 [rs11599004 (chr10:51488377)], p53decamer [rs144495132 (chr10:51565358)]
BHLHA15	AFP1 [rs2132276 (chr7:97826339)], BLIMP1 [rs6465658 (chr7:97816637)], CACD [rs6465658 (chr7:97816637)], E2A [rs35983874 (chr7:97916460)], E47 [rs6968193 (chr7:97915901)], FOXM1 [rs11772293 (chr7:97925930)], GLI1 [rs7778015 (chr7:97920269)], HMGIY [rs141996715 (chr7:97676379)], Hand1:E47 [rs6945554 (chr7:97972945)], INSM1 [rs34616135 (chr7:97840144)], IPF1 [rs34124383 (chr7:98027787)], IRF-7 [rs3779193 (chr7:98028465)], MAF [rs34616135 (chr7:97840144)], MEF-2 [rs12666406 (chr7:97790990)], MZF1 [rs3801251 (chr7:98027617)], NFE2L2 [rs34616135 (chr7:97840144)], Nrf-2 [rs34616135 (chr7:97840144)], P50:RELA-P65 [rs10953255 (chr7:97923197)], Pax-4 [rs10953255 (chr7:97923197)], RREB-1 [rs7778015 (chr7:97920269)], RREB1 [rs7778015 (chr7:97920269)], SREBP [rs7778015 (chr7:97920269)], Spz1 [rs6465663 (chr7:97918882)], TBX5 [rs34835415 (chr7:97962647)], TP53 [rs6965016 (chr7:97807881)], Zfx [rs34695842 (chr7:97931061), rs73147342 (chr7:97928721)]
C19orf48	CTCF [rs2569451* (chr19:51520199)], Freac-7 [rs11665698 (chr19:51354410)], Pax-4 [rs2075693 (chr19:51518497)], SOX [rs2659051* (chr19:51345567)], SOX2 [rs2659051* (chr19:51345567)], Sp1 [rs2569451* (chr19:51520199)]
FAM57A	INSM1 [rs2295479 (chr17:636821)], LRF [rs2456274* (chr17:618407)], MZF1 [rs2291779* (chr17:653986)], MZF1_5-13 [rs2291779* (chr17:653986)], RREB1 [rs2295479 (chr17:636821)], Sox2 [rs3087833 (chr17:657729)], VDR [rs865702 (chr17:568152)]
GEMIN4	VDR [rs865702 (chr17:568152)]
HNF1B	Ikaros [rs9901746* (chr17:36103148)], MEF2A [rs8064454* (chr17:36101585)], PPARdirectrepeat1 [rs11263762* (chr17:36101925)], Zfx [rs757209* (chr17:36102832)]
HOXA4	Ikaros [rs2237340 (chr7:27779764)], Klf4 [rs72598505 (chr7:27755171)], Sp1 [rs28372805 (chr7:27702801)]
KLK1	CACD [rs266128* (chr19:51296491)], E2F [rs55716643* (chr19:51297824), rs55735528* (chr19:51297825)], E2F-1 [rs55716643* (chr19:51297824), rs55735528* (chr19:51297825)], E47 [rs7258708* (chr19:51299644)], GCNF [rs9991* (chr19:51301229)], LXR [rs3833237* (chr19:51302473)], MTF-1 [rs11084033* (chr19:51353954)], NF-kappaB [rs55716643* (chr19:51297824)], NFKB1 [rs55716643* (chr19:51297824)], PPARG::RXRA [rs7258708* (chr19:51299644)], PPARgamma:RXRalpha [rs7258708* (chr19:51299644)], ZBRK1 [rs1043315* (chr19:51302153)]

Gene	LNCaP Footprinting TFBS [Variant(s) (hg19 position)]
NCOA4	Cart-1 [rs4935090 (chr10:51491124)], HFH4(FOXJ1) [rs2611507 (chr10:51518672)], HNF3 [rs2611507 (chr10:51518672)], INSM1 [rs12783102 (chr10:51489666)], PPARG [rs2611471 (chr10:51503558)], Pitx3 [rs12783102 (chr10:51489666)], TFII-I [rs2611491 (chr10:51489392)]
PPP1R14A	MAZR [rs12611084 (chr19:38746271)], MEF-2 [rs376018088 (chr19:38284426)]
STK25	BLIMP1 [rs61604879 (chr2:242172408)], CIZ [rs35094131* (chr2:242126210)], CP2 [rs1348611 (chr2:242087890)], Cdc5 [rs7568 (chr2:242291742)], GR [rs7568 (chr2:242291742)], IRF2 [rs12618809 (chr2:242249121)], NRSF [rs62186360 (chr2:242166211)], PLAG1 [rs111937035 (chr2:242212176)], PPARG [rs144426547 (chr2:242198934)], PU.1 [rs61604879 (chr2:242172408)], Pbx [rs56390510* (chr2:242274488)], REST [rs62186360 (chr2:242166211)], SREBP [rs1064767 (chr2:242167336)], Zic2 [rs34577714 (chr2:242046993)]
TMPRSS2	Gfi1b [rs13433394 (chr21:42831909)], HNF1 [rs56095453 (chr21:42893807)], HNF1A [rs56095453 (chr21:42893807)], Lhx3 [rs118060196 (chr21:43305854)], NF-AT [rs34205539* (chr21:42871545)], OTX [rs118060196 (chr21:43305854)], Pax-2 [rs56095453 (chr21:42893807)]
VPS53	AP-4 [rs12952217* (chr17:1018425)], ARP-1(COUP-TF2) [rs55883617 (chr17:836107)], E2F [rs2440054 (chr17:1031751)], FOXO1 [rs2254247 (chr17:629272)], LRF [rs2456274* (chr17:618407)], MEF-2 [rs74365978 (chr17:527849)], Pitx2 [rs189251824 (chr17:406736)], RSRFC4 [rs74365978 (chr17:527849)], VDR [rs865702 (chr17:568152)]

* Directly modeled eQTL variants in VCaP ChIP-Seq TFBS. Remaining variants in LD (r^2 ≥ 0.8 in 1000 Genomes Phase III EUR) with a modeled eQTL variant.

Supplementary Table 12. Pathway Analysis of Transcription Factors with Non-Prostate Cancer Cell Line ChIP-Seq Peaks at or tagged by eQTL Variants for Nominally Replicated Genes

**PANTHER Overrepresentation Test of Reactome Pathways** (Fisher's Exact Test)			
**Tested Transcription Factors:** ARID3A, ATF1, ATF2, ATF3, BACH1, BATF, BCL11A, BCL3, BCLAF1, BHLHE40, BRCA1, CBX3, CCNT2, CDX2, CEBPB, CEBPD, CHD1, CHD2, CREB1, CREBBP, CTBP2, CTCF, CTCFL, E2F1, E2F4, E2F6, EBF1, EGR1, ELF1, ELK1, ELK4, EP300, ESR1, ETS1, EZH2, FOS, FOSL1, FOSL2, FOXA1, FOXA2, FOXM1, FOXP2, GABPA, GABPB1, GATA1, GATA2, GATA3, GATA6, GTF2B, GTF2F1, HDAC1, HDAC2, HDAC6, HMGN3, HNF4A, HNF4G, HSF1, IKZF1, IRF1, IRF3, IRF4, JUN, JUNB, JUND, KDM5A, KDM5B, MAFF, MAFK, MAX, MAZ, MBD4, MEF2A, MEF2C, MEIS1, MTA3, MXI1, MYBL2, MYC, NANOG, NFATC1, NFE2, NFIC, NFKB1, NFYA, NFYB, NR2F2, NR3C1, NRF1, PAX5, PBX3, PHF8, PML, POLR2A, POU2F2, PRDM1, RAD21, RBBP5, RCOR1, REST, RFX3, RFX5, RUNX3, RXRA, SAP30, SETDB1, SIN3A, SIRT6, SIX5, SMARCA4, SMARCB1, SMARCC1, SMARCC2, SMC3, SP1, SP2, SP4, SPI1, SREBF1, SRF, STAT1, STAT2, STAT3, STAT5A, SUZ12, TAF1, TAF7, TAL1, TBL1XR1, TBP, TCF12, TCF3, TCF7L2, TEAD4, TFAP2A, TFAP2C, THAP1, TRIM28, UBTF, USF1, USF2, WRNIP1, YY1, ZBTB33, ZBTB7A, ZEB1, ZKSCAN1, ZNF143, ZNF217, ZNF263, ZNF274			
**Reactome Pathway**	**Fold Enrich-ment**	**Raw *P*-value**	**FDR**
TFAP2 (AP-2) family regulates transcription of cell cycle factors	>100	$3.54*10^{-7}$	$1.53*10^{-5}$
↳ Transcriptional regulation by the AP-2 (TFAP2) family of transcription factors	36.6	$1.93*10^{-11}$	$4.79*10^{-9}$
↳ Generic Transcription Pathway	6.11	$3.64*10^{-19}$	$7.24*10^{-16}$
↳ Gene Expression	3.9	$6.30*10^{-17}$	$6.27*10^{-14}$
HDACs deacetylate histones	17.7	$2.97*10^{-7}$	$1.34*10^{-5}$
↳ Chromatin modifying enzymes	12.6	$1.38*10^{-16}$	$9.12*10^{-14}$
↳ Chromatin organization	12.6	$1.38*10^{-16}$	$6.84*10^{-14}$
Unclassified	0.47	$7.16*10^{-14}$	$2.85*10^{-11}$
Factors involved in megakaryocyte development and platelet production	12.9	$2.64*10^{-12}$	$8.77*10^{-10}$
↳ Hemostasis	3.43	$4.21*10^{-5}$	0.00101
Regulation of TP53 Activity through Acetylation	23.3	$4.45*10^{-5}$	0.00105

**PANTHER Overrepresentation Test of Reactome Pathways** (Fisher's Exact Test)			
**Tested Transcription Factors:** ARID3A, ATF1, ATF2, ATF3, BACH1, BATF, BCL11A, BCL3, BCLAF1, BHLHE40, BRCA1, CBX3, CCNT2, CDX2, CEBPB, CEBPD, CHD1, CHD2, CREB1, CREBBP, CTBP2, CTCF, CTCFL, E2F1, E2F4, E2F6, EBF1, EGR1, ELF1, ELK1, ELK4, EP300, ESR1, ETS1, EZH2, FOS, FOSL1, FOSL2, FOXA1, FOXA2, FOXM1, FOXP2, GABPA, GABPB1, GATA1, GATA2, GATA3, GATA6, GTF2B, GTF2F1, HDAC1, HDAC2, HDAC6, HMGN3, HNF4A, HNF4G, HSF1, IKZF1, IRF1, IRF3, IRF4, JUN, JUNB, JUND, KDM5A, KDM5B, MAFF, MAFK, MAX, MAZ, MBD4, MEF2A, MEF2C, MEIS1, MTA3, MXI1, MYBL2, MYC, NANOG, NFATC1, NFE2, NFIC, NFKB1, NFYA, NFYB, NR2F2, NR3C1, NRF1, PAX5, PBX3, PHF8, PML, POLR2A, POU2F2, PRDM1, RAD21, RBBP5, RCOR1, REST, RFX3, RFX5, RUNX3, RXRA, SAP30, SETDB1, SIN3A, SIRT6, SIX5, SMARCA4, SMARCB1, SMARCC1, SMARCC2, SMC3, SP1, SP2, SP4, SPI1, SREBF1, SRF, STAT1, STAT2, STAT3, STAT5A, SUZ12, TAF1, TAF7, TAL1, TBL1XR1, TBP, TCF12, TCF3, TCF7L2, TEAD4, TFAP2A, TFAP2C, THAP1, TRIM28, UBTF, USF1, USF2, WRNIP1, YY1, ZBTB33, ZBTB7A, ZEB1, ZKSCAN1, ZNF143, ZNF217, ZNF263, ZNF274			
**Reactome Pathway**	**Fold Enrichment**	**Raw *P*-value**	**FDR**
↳ Regulation of TP53 Activity	8.82	$3.67*10^{-7}$	$1.55*10^{-5}$
↳ Transcriptional Regulation by TP53	7.64	$4.73*10^{-12}$	$1.34*10^{-9}$
RORA activates gene expression	24.8	$3.58*10^{-6}$	$1.02*10^{-4}$
↳ Circadian Clock	21.6	$1.35*10^{-10}$	$2.43*10^{-8}$
Activation of anterior HOX genes in hindbrain development during early embryogenesis	17.3	$1.33*10^{-10}$	$2.93*10^{-8}$
↳ Activation of HOX genes during differentiation	17.3	$1.33*10^{-10}$	$2.64*10^{-8}$
Activation of the AP-1 family of transcription factors	40.2	$1.08*10^{-4}$	0.00228
↳ MAPK targets/ Nuclear events mediated by MAP kinases	35.7	$3.06*10^{-10}$	$5.08*10^{-8}$
↳ MAP kinase activation in TLR cascade	20.1	$2.06*10^{-9}$	$2.74*10^{-7}$
CDO in myogenesis	19.2	$8.86*10^{-5}$	0.00196
↳ Myogenesis	19.2	$8.86*10^{-5}$	0.00198
↳ Developmental Biology	4.37	$3.96*10^{-10}$	$6.06*10^{-8}$
ERCC6 (CSB) and EHMT2 (G9a) positively regulate rRNA expression	13.8	$2.85*10^{-4}$	0.00541

**PANTHER Overrepresentation Test of Reactome Pathways** (Fisher's Exact Test)			
**Tested Transcription Factors:** ARID3A, ATF1, ATF2, ATF3, BACH1, BATF, BCL11A, BCL3, BCLAF1, BHLHE40, BRCA1, CBX3, CCNT2, CDX2, CEBPB, CEBPD, CHD1, CHD2, CREB1, CREBBP, CTBP2, CTCF, CTCFL, E2F1, E2F4, E2F6, EBF1, EGR1, ELF1, ELK1, ELK4, EP300, ESR1, ETS1, EZH2, FOS, FOSL1, FOSL2, FOXA1, FOXA2, FOXM1, FOXP2, GABPA, GABPB1, GATA1, GATA2, GATA3, GATA6, GTF2B, GTF2F1, HDAC1, HDAC2, HDAC6, HMGN3, HNF4A, HNF4G, HSF1, IKZF1, IRF1, IRF3, IRF4, JUN, JUNB, JUND, KDM5A, KDM5B, MAFF, MAFK, MAX, MAZ, MBD4, MEF2A, MEF2C, MEIS1, MTA3, MXI1, MYBL2, MYC, NANOG, NFATC1, NFE2, NFIC, NFKB1, NFYA, NFYB, NR2F2, NR3C1, NRF1, PAX5, PBX3, PHF8, PML, POLR2A, POU2F2, PRDM1, RAD21, RBBP5, RCOR1, REST, RFX3, RFX5, RUNX3, RXRA, SAP30, SETDB1, SIN3A, SIRT6, SIX5, SMARCA4, SMARCB1, SMARCC1, SMARCC2, SMC3, SP1, SP2, SP4, SPI1, SREBF1, SRF, STAT1, STAT2, STAT3, STAT5A, SUZ12, TAF1, TAF7, TAL1, TBL1XR1, TBP, TCF12, TCF3, TCF7L2, TEAD4, TFAP2A, TFAP2C, THAP1, TRIM28, UBTF, USF1, USF2, WRNIP1, YY1, ZBTB33, ZBTB7A, ZEB1, ZKSCAN1, ZNF143, ZNF217, ZNF263, ZNF274			
**Reactome Pathway**	**Fold Enrich-ment**	**Raw *P*-value**	**FDR**
↳ Positive epigenetic regulation of rRNA expression	11.8	$1.83*10^{-5}$	$4.80*10^{-4}$
↳ Epigenetic regulation of gene expression	13.4	$1.64*10^{-9}$	$2.33*10^{-7}$

Supplementary Table 13. Modeled eQTL Variants for Nominally Replicated Genes

Gene	Modeled eQTL Variant(s): dbSNP rsid's (top row), and Effect size * [chr].[hg19 position].[reference allele].[effect allele] (bottom row)
MSMB	rs10993994
	0.137382087372 * chr10.51549496.T.C
NCOA4	rs10761581, rs10797293, rs10821609, rs10857544, rs10993994, rs10994470, rs11005000, rs11005001, rs113007204, rs11593018, rs117007447, rs11815907, rs12770171, rs140400658, rs149459027, rs199737079, rs201553402, rs2574950, rs2574979, rs2611492, rs2813326, rs2843566, rs3032323, rs35931666, rs36081243, rs3829175, rs4245023, rs4267018, rs4390270, rs55848837, rs61847076, rs61847077, rs61858186, rs6537554, rs7073005, rs7350420, rs78937603, rs7905981, rs7911198
	-0.00894975773274 * chr10.51568378.T.G + 0.0053262686578 * chr10.51835528.G.C + -0.0100170608091 * chr10.51550917.C.T + -7.60408177822e-05 * chr10.51086922.T.C + -0.2495579856 * chr10.51549496.T.C + -0.019690752174 * chr10.51558660.G.A + 0.00637687964868 * chr10.52054965.T.C + 0.000910455747353 * chr10.52054970.G.A + -0.000231873538128 * chr10.51593069.GACA.G + 0.000761879352574 * chr10.52055245.G.A + 0.109067814805 * chr10.52058576.T.C + 0.00595177814957 * chr10.52054796.T.C + 0.0357638592524 * chr10.51549314.C.T + 0.00785398673495 * chr10.51831312.A.G + -0.00880980208611 * chr10.51086267.G.A + 0.000414694880872 * chr10.52059234.CCCCG.C + -0.00931080615415 * chr10.51486999.G.A + 0.010577733828 * chr10.52040307.T.A + 0.464074907611 * chr10.52073163.A.C + -0.00786138331763 * chr10.51489370.C.G + 0.0125046334695 * chr10.52038589.G.A + -0.00791445008919 * chr10.51489367.C.T + -0.0021681297676 * chr10.51520177.GT.G + -0.00188236610413 * chr10.51520488.T.TAA + 0.000608972401374 * chr10.52055431.GA.G + 0.00110085119848 * chr10.51127454.G.A + 0.00437469046241 * chr10.51812441.A.C + 0.00304823805185 * chr10.52056410.G.A + -0.00114939310707 * chr10.51101320.C.T + -0.00656208544015 * chr10.51502552.G.T + -0.00172837018682 * chr10.51591402.C.G + -0.00182008995604 * chr10.51591433.C.T + 0.000785786671181 * chr10.52059207.A.G + 3.70640325888e-05 * chr10.51104456.A.G + 0.00200382301535 * chr10.52054031.C.T + 0.0126773682794 * chr10.51594462.T.C + -0.00150812936556 * chr10.51580467.C.G + -0.0122302730826 * chr10.51128662.G.A + -0.0391468481301 * chr10.51537475.G.A
HNF1B	rs11263761, rs11263762, rs11263763, rs11651052, rs12453443, rs12945081, rs2522964, rs34444303, rs3760511, rs67143603, rs7405696, rs757209, rs8064454, rs9901746
	0.0176642411056 * chr17.36097775.G.A + 0.0106249688443 * chr17.36101926.G.A + 0.00593439653366 * chr17.36103565.G.A + 0.0164237656249 * chr17.36102381.A.G + 0.00383561821556 * chr17.36104121.C.G + -0.00433697040406 * chr17.36139423.G.A + -0.200489148648 * chr17.35809215.T.A + -0.0135539048535 * chr17.36146301.G.A + 0.00274038763262 * chr17.36106313.T.G + 0.0137098504649 * chr17.36108231.T.C + 0.00640905880796 * chr17.36102035.G.C + 0.00807980627394 * chr17.36102833.G.A + 0.0212022148065 * chr17.36101586.A.C + 0.0108593178563 * chr17.36103149.G.A

Gene	Modeled eQTL Variant(s):<br>dbSNP rsid's (top row), and<br>Effect size * [chr].[hg19 position].[reference allele].[effect allele] (bottom row)
AGAP7	rs10708919, rs10824973, rs10824974, rs10826223, rs10993994, rs10994577, rs11003511, rs11284399, rs113007204, rs11327043, rs11548236, rs11592181, rs117156369, rs12146177, rs12770171, rs12777595, rs142470094, rs143254339, rs17178655, rs200598858, rs28671507, rs34726772, rs371975360, rs373320530, rs3849145, rs4935065, rs570896036, rs59363341, rs61847076, rs61847077, rs7070965, rs71502415, rs75933669, rs76874542, rs78937603, rs7905112, rs79995577
	-0.0164381253676 * chr10.50984396.TA.T + -0.000133144679 * chr10.51807210.T.C + -0.00195796863568 * chr10.51807214.G.A + -0.0258779900236 * chr10.51538825.G.A + -0.0949913982669 * chr10.51549496.T.C + -0.0532322726589 * chr10.51561241.G.A + -0.00310934830515 * chr10.51808564.C.T + -0.0528098666038 * chr10.50992529.CT.C + -0.00262505782264 * chr10.51593069.GACA.G + -0.0191389426875 * chr10.51807447.TC.T + 1.80731815775 * chr10.51589581.C.T + -0.061318256217 * chr10.51582686.G.A + -0.143398918738 * chr10.50970768.C.T + -0.00414741934056 * chr10.51807814.G.T + 0.000842146508394 * chr10.51549314.C.T + 0.208275983265 * chr10.51500625.G.C + -0.0162242018032 * chr10.51834814.A.ATG + -0.00586362861622 * chr10.51807458.T.G + -0.0106407482386 * chr10.51561799.G.A + -0.0824639873494 * chr10.51510383.C.CA + 0.0134645833032 * chr10.51820916.C.A + 0.641422180216 * chr10.50979780.AT.TT + -0.019542588235 * chr10.51505009.GA.G + 0.0209086549522 * chr10.51602606.C.A + 0.0257423733021 * chr10.51049548.G.A + 0.00962156672871 * chr10.51814612.T.C + 6.35504883565 * chr10.51834675.C.CAAAAAAAATAAT + -3.37563385649e-05 * chr10.51810982.C.A + -0.00391681333647 * chr10.51591402.C.G + -0.00411826106369 * chr10.51591433.C.T + -0.0370849915106 * chr10.51560207.G.A + 0.213355801062 * chr10.51546115.G.A + 0.0186975295641 * chr10.51563351.G.T + 1.37827784019 * chr10.51006811.C.T + -0.00389823208345 * chr10.51580467.C.G + 0.00361897717409 * chr10.51807402.T.G + -0.0628093030571 * chr10.51508576.T.C
POU5F1B	rs6470510
	-0.0431840297396 * chr8.128429660.T.C
C19orf48	rs10421146, rs10421906, rs10445554, rs11084032, rs11665748, rs11668685, rs200343323, rs2042905, rs2091181, rs2411331, rs2569451, rs2569748, rs2659051, rs266121, rs2691225, rs2691226, rs2691227, rs2691229, rs2739443, rs28665094, rs34988149, rs368439913, rs41275784, rs4239492, rs4801855, rs4802742, rs4802751, rs62114265, rs67013619, rs7250580, rs73584983, rs73592857, rs75759612, rs78177998, rs79178205, rs79473488
	0.00306265890853 * chr19.51290110.C.T + 5.63081634606e-06 * chr19.51290499.C.G + 0.00306057280778 * chr19.51349716.T.C + 0.00214925200808 * chr19.51349602.A.G + 0.00439840993708 * chr19.51354397.G.A + 0.0157733001015 * chr19.51304566.A.C + 0.00670821927457 * chr19.51351233.TC.T + 0.00631532408493 * chr19.51288886.G.C + -0.00782146656458 * chr19.51592564.G.T + 0.00102856175279 * chr19.51350880.T.A + -0.0038233278571 * chr19.51520200.C.T + -0.0217730628708 * chr19.51341455.G.A + 0.00277007588133 * chr19.51345568.C.G + -0.0230028300193 * chr19.51299959.C.G + 0.00380311279741 * chr19.51545463.T.C + 0.00590257323745 * chr19.51545481.A.G + 0.00394336591438 * chr19.51545746.G.A

Gene	Modeled eQTL Variant(s): dbSNP rsid's (top row), and Effect size * [chr].[hg19 position].[reference allele].[effect allele] (bottom row)
	+ 0.00592373866007 * chr19.51546642.T.C + -0.0172018849026 * chr19.51341326.G.A + 0.00418587908405 * chr19.51472388.C.T + -0.00679562092422 * chr19.51341672.T.A + 1.27243205496e-05 * chr19.51545899.C.CAAA + 0.0229570780608 * chr19.51191057.A.C + 0.00340393981385 * chr19.51348711.C.T + 0.00329791092634 * chr19.51348572.T.G + 0.0131001014862 * chr19.51303304.A.C + 0.00406584327004 * chr19.51348229.G.C + -0.0496882556072 * chr19.51260408.T.C + 0.00726900652243 * chr19.51288968.GT.G + -0.00458819656997 * chr19.51519803.A.G + 0.00238487528189 * chr19.51290173.A.G + 0.0211927824316 * chr19.51377607.A.G + 0.0206363768769 * chr19.51374807.G.T + -0.0170806221405 * chr19.51345264.T.C + 0.0245842204102 * chr19.51374738.T.A + 0.0237211984311 * chr19.51374748.C.G
KLK15	rs2659124, rs266863, rs266878, rs78177998
	0.0238490159221 * chr19.51354597.A.T + 0.0683401469369 * chr19.51355650.T.C + 0.0108892759773 * chr19.51359114.G.C + -0.0210133552355 * chr19.51345264.T.C
PCAT1	rs10481163, rs10481164, rs11448589, rs1551513, rs1551515, rs16901904, rs17762878, rs17762938, rs2035637, rs4473999, rs4573233, rs55986178, rs6651240, rs7015383, rs73351641, rs74485418, rs7823297, rs7823764, rs7824011, rs7824393, rs78316206, rs7842175, rs7844107, rs9656964
	0.00434644993826 * chr8.128027957.C.T + 0.00416499165824 * chr8.128027962.G.A + 0.00469715477781 * chr8.128026938.T.TA + 0.00419227410122 * chr8.128025599.T.C + 0.00643664585733 * chr8.128025404.T.A + 0.00452411784113 * chr8.128027502.T.C + 0.000265069708467 * chr8.128025053.A.G + 0.00578785355167 * chr8.128025857.T.C + 0.00947153815595 * chr8.128023059.C.A + 0.00600236049439 * chr8.128025762.C.T + 0.00665868785762 * chr8.128025263.G.A + -0.0202266345905 * chr8.127880198.T.G + 0.012563543274 * chr8.128026046.A.T + -0.0123143977939 * chr8.127877650.G.T + 0.00680111210722 * chr8.128025212.G.A + 0.038983875254 * chr8.128010900.C.T + 0.00566990692551 * chr8.128025916.C.T + 0.0051718210671 * chr8.128026262.A.G + 0.00530684882918 * chr8.128026203.G.C + 0.00486403229287 * chr8.128026684.A.G + 0.0489450044711 * chr8.128019309.C.T + 0.00502307449685 * chr8.128026317.T.C + 0.00764242098458 * chr8.128023386.G.A + 0.00591192532044 * chr8.128025777.C.G
FAM57A	rs12601740, rs150674644, rs2291779, rs2456274, rs2474694, rs2644715, rs331013, rs34623718, rs35334456, rs434307, rs444385, rs461251, rs4968105, rs4968131, rs4968165, rs555854868, rs59580892, rs60060532, rs62053809, rs67456235, rs684232, rs71358511, rs72810245, rs7406992, rs79350209, rs866304
	-0.0124022348699 * chr17.500930.G.A + 0.169097544159 * chr17.513245.G.T + 0.00193676015626 * chr17.653987.T.C + -0.0546123536101 * chr17.618408.C.A + -0.018276552175 * chr17.618039.G.A + 0.0576312442288 * chr17.632686.G.A + 0.000290952996585 * chr17.530790.A.G + -0.00910534216656 * chr17.642578.G.A + -0.00309884130924 * chr17.641579.G.A + 0.00018815749347 * chr17.554080.C.T + 0.00906572870338 * chr17.474543.G.A + -0.0354891941649 * chr17.619162.A.G + 0.00373084447143 * chr17.652520.G.A + -0.00129063209071 * chr17.851747.T.C + 0.0122287234505 * chr17.470314.A.C + -0.0107561716841 * chr17.559940.ATG.A + 0.00169111453502 * chr17.654039.T.G + -0.00012697937473 * chr17.581194.C.T + -

Gene	Modeled eQTL Variant(s):<br>dbSNP rsid's (top row), and<br>Effect size * [chr].[hg19 position].[reference allele].[effect allele] (bottom row)
	0.000253342457818 * chr17.595538.T.C + 0.00118017329531 * chr17.651744.G.A + -0.0172601309388 * chr17.618965.T.C + -0.00249845702506 * chr17.645583.C.T + 0.0185225664779 * chr17.637644.G.T + 0.00553634905784 * chr17.839964.T.C + -0.00477583103008 * chr17.1010578.A.G + 0.0227533231577 * chr17.587729.T.C
PPP1R14A	rs11667256, rs16975963, rs241955, rs4802297, rs76443179, rs78558945, rs8102454, rs8102476
	-0.0916856234536 * chr19.38735804.A.T + -0.000524997762054 * chr19.38325536.C.G + -0.0184406198822 * chr19.38278677.A.G + -0.107508387273 * chr19.38738130.G.C + 0.0017409209128 * chr19.38669622.G.A + 0.0119593923459 * chr19.38695262.G.A + -0.0438319491267 * chr19.38735480.G.A + -0.00607730868168 * chr19.38735613.C.T
TMPRSS2	rs11463654, rs115578024, rs117016829, rs146564124, rs1557370, rs28360562, rs2839413, rs34205539, rs34983238, rs4327307, rs55992492, rs56095453, rs60687893, rs73231906, rs79783160, rs79872290, rs9978557, rs9979885, rs9984523
	-0.0102969463693 * chr21.42615842.C.CT + -0.0800036525789 * chr21.42465543.T.C + 0.0492252837785 * chr21.43307925.G.A + -0.041305801796 * chr21.42837861.C.T + -0.0358688118432 * chr21.42830690.A.G + -0.116019464346 * chr21.42878126.A.C + 0.00629164112659 * chr21.43316078.G.A + -0.0543239611019 * chr21.42871545.A.AT + -0.0200658280075 * chr21.42868997.A.C + -0.0114960724042 * chr21.42901033.G.A + -0.0229050986786 * chr21.42830902.T.A + 0.000907537203622 * chr21.42893808.T.A + -0.0238165702039 * chr21.42830901.A.G + 0.0622456885647 * chr21.42892348.C.T + 0.0102475802835 * chr21.43318259.C.T + -0.220301885661 * chr21.42950876.C.A + -0.106451363426 * chr21.42882462.C.T + -0.0194301704637 * chr21.42886232.C.T + -0.0212779026067 * chr21.42884031.C.T
GEMIN4	rs2467247, rs2474694, rs2740345, rs2740358, rs2750006, rs330999, rs331000, rs410157, rs461251, rs58042979, rs684232, rs866304
	0.00348546234718 * chr17.671146.C.T + -0.00184042938832 * chr17.618039.G.A + 0.0071462388132 * chr17.667377.G.A + 0.00678810236252 * chr17.617059.T.C + 0.00126415335444 * chr17.659371.G.A + 0.00303055432413 * chr17.609658.A.T + 0.00265569057402 * chr17.609506.T.C + 0.000270842613009 * chr17.598240.T.C + -0.0160820796779 * chr17.619162.A.G + 0.00584130291999 * chr17.675201.C.T + -0.00205545553941 * chr17.618965.T.C + 0.0113949377251 * chr17.587729.T.C
BHLHA15	rs10953238, rs111849767, rs11760930, rs34649939, rs34672324, rs34838763, rs35527492, rs6465657, rs67420939, rs75021532, rs77191343, rs7790229
	-0.0257104557007 * chr7.97676259.C.T + -0.0140635826194 * chr7.97911618.G.A + -1.66211862928e-05 * chr7.97915789.G.A + -0.00511897399717 * chr7.97849359.CA.C + -0.0184295579838 * chr7.97989237.A.AT + -0.00010039582709 * chr7.97898810.C.CT + -0.0146750578599 * chr7.97849464.C.T + -0.00156960865299 * chr7.97816327.C.T + -2.45703120296e-05 * chr7.97850599.A.C + -0.037316085092 * chr7.97949650.G.A + -0.101611487644 * chr7.97978909.G.A + -

Gene	Modeled eQTL Variant(s):<br>dbSNP rsid's (top row), and<br>Effect size * [chr].[hg19 position].[reference allele].[effect allele] (bottom row)
	0.000365598477451 * chr7.97919338.G.A
ZFP36L2	rs1038822, rs115425585, rs7600872
	0.0200002093389 * chr2.43738173.T.C + 0.0181183454022 * chr2.43741596.T.A + 0.00430544688611 * chr2.43789231.T.C
STK25	rs10187363, rs10933546, rs10933548, rs11677414, rs11884197, rs11896191, rs12612578, rs12619677, rs141659095, rs143748637, rs144944572, rs151280108, rs201892214, rs35094131, rs56061376, rs56390510, rs58226367, rs59542139, rs60515663, rs62186363, rs62186405, rs62186406, rs62186408, rs62186410, rs62186419, rs62186420, rs67625947, rs72484048, rs73002114, rs75011974, rs9679681
	0.00299945864137 * chr2.242053823.C.T + -0.00058306111082 * chr2.242225998.T.A + -0.000458427179922 * chr2.242234837.T.C + -0.000256800381638 * chr2.242237797.G.A + -0.000364387906858 * chr2.242242786.C.T + -0.000569227762938 * chr2.242226751.T.C + -0.000405469687864 * chr2.242236660.G.A + -0.000540988503264 * chr2.242213195.A.G + -0.00171472917614 * chr2.242317685.AT.A + -0.00129155229699 * chr2.242280809.TACTA.T + -0.0013297205376 * chr2.242921866.CTG.C + -0.00192277168033 * chr2.242312353.C.CT + 0.000302753730719 * chr2.242043565.CAAA.C + 0.00167883173069 * chr2.242126210.GA.G + -0.000357450806604 * chr2.242243435.T.C + -0.00124400006102 * chr2.242274489.G.C + -0.000518734114298 * chr2.242227547.C.T + -0.000594380818493 * chr2.242213649.T.C + -0.000593239333926 * chr2.242214258.G.A + -0.00931176362478 * chr2.242176429.G.A + -0.000497855085793 * chr2.242232153.C.G + -0.000477674360028 * chr2.242233456.A.G + -0.000440055621908 * chr2.242235651.A.G + -0.000422424517483 * chr2.242235858.T.C + -0.000344761624544 * chr2.242246865.C.T + -0.000331453962084 * chr2.242246875.T.C + -0.000539530075697 * chr2.242227400.C.A + -0.000519437988534 * chr2.242227359.G.C + -0.00128980338326 * chr2.242280924.G.A + -0.00206683471309 * chr2.241940582.C.T + 0.00297475496895 * chr2.242147264.C.A
HOXA4	rs12670250, rs13245339, rs147853527, rs17427421, rs55947942, rs572478750, rs6957243
	0.0069169410998 * chr7.27577103.A.G + 0.00344771096665 * chr7.27562097.T.C + -0.00256440157884 * chr7.27040802.G.A + -0.00658285648347 * chr7.27118974.C.T + -0.0572972369606 * chr7.27611838.A.T + -0.0273058261967 * chr7.26938274.ATTTTTT.A + -0.00769292432381 * chr7.27440061.C.T
VPS53	rs10708313, rs111583421, rs111764301, rs112189013, rs114972153, rs11652688, rs1237094, rs12449775, rs12952217, rs140168243, rs145868182, rs16953156, rs17620240, rs183156235, rs2456274, rs2543778, rs2740358, rs28396482, rs330999, rs331000, rs34185510, rs34699431, rs370503694, rs410157, rs4280315, rs437948, rs4531806, rs502018, rs555854868, rs55842940, rs6598823, rs7405543, rs7406992, rs76404864, rs77032846, rs77114150, rs77432469, rs79547623, rs8069166, rs9907824, rs9910739, rs9916862

Gene	Modeled eQTL Variant(s):<br>dbSNP rsid's (top row), and<br>Effect size * [chr].[hg19 position].[reference allele].[effect allele] (bottom row)
	0.00298129077698 * chr17.611084.CA.C + 0.00195062562676 * chr17.798817.T.C + -0.00613046163368 * chr17.167086.T.C + -0.00596664716686 * chr17.1015861.A.AAGGAAGGAAGAG + -0.00338818791926 * chr17.869131.G.A + 0.000410485449557 * chr17.278302.A.G + -0.00618916427239 * chr17.619842.C.T + -0.0173870631382 * chr17.286649.C.T + 0.0198549114246 * chr17.1018426.C.G + 0.0138650982732 * chr17.1040403.C.T + -0.000184660719197 * chr17.837689.G.GA + 0.000585399094848 * chr17.424880.G.A + -0.00248607380249 * chr17.833158.T.G + -0.00453669234117 * chr17.1013159.C.T + -0.00414880836343 * chr17.618408.C.A + 0.00788759715298 * chr17.565745.C.T + 0.00273519223973 * chr17.617059.T.C + 0.00111313653078 * chr17.513349.C.T + 0.0043351266551 * chr17.609658.A.T + 0.00587696757966 * chr17.609506.T.C + 0.00463763465831 * chr17.1016613.G.A + 0.006638399938 * chr17.581890.A.T + -0.00736333372148 * chr17.167104.T.G + 0.00621332619983 * chr17.598240.T.C + -0.00866766645637 * chr17.188593.C.T + -0.00795426797882 * chr17.623356.C.G + -0.00387866475792 * chr17.151348.G.T + 0.0142911183555 * chr17.631307.C.T + 0.00749022132583 * chr17.559940.A.G + 1.51475771512e-06 * chr17.160523.G.A + 0.00544940504489 * chr17.610094.G.C + -7.53717157369e-05 * chr17.1038663.G.A + 0.0146910407207 * chr17.839964.T.C + 0.00353161645929 * chr17.293274.A.G + 0.0138399043631 * chr17.123961.G.A + 0.0201782533238 * chr17.276966.T.A + -0.00135914436027 * chr17.713598.C.T + 0.0083116137637 * chr17.240172.G.C + 0.00323314487033 * chr17.293138.G.A + 0.477739322465 * chr17.813875.G.A + 0.0402301617326 * chr17.421755.A.G + -0.00801902962172 * chr17.1038188.A.G
KLK1	rs10401588, rs1043315, rs10445554, rs1061476, rs1061477, rs11084032, rs11084033, rs1135766, rs11573, rs11668998, rs12610777, rs1654513, rs200343323, rs2292185, rs2411331, rs2456585, rs2560931, rs2560933, rs2659103, rs2659104, rs266114, rs266122, rs266123, rs266125, rs266127, rs266128, rs3745526, rs3833237, rs4239492, rs4801853, rs4801855, rs4802741, rs4802751, rs529333024, rs547476048, rs55716643, rs55735528, rs55929248, rs560911495, rs62113224, rs7258708, rs73592857, rs925013, rs9991
	0.00777013304966 * chr19.51305287.A.G + 0.00225280727302 * chr19.51302154.G.A + 0.003348928652 * chr19.51349716.T.C + 0.0112736509913 * chr19.51360087.G.A + 0.00498653350085 * chr19.51360096.T.C + 0.00396236238451 * chr19.51349602.A.G + 0.0587706899223 * chr19.51353955.C.A + 0.0278058515406 * chr19.51359503.A.G + 0.0273384543727 * chr19.51359497.T.C + 0.00271511004203 * chr19.51299342.C.T + 0.00273032259905 * chr19.51303727.T.C + -0.0256729957034 * chr19.51402682.C.T + 0.0348077751087 * chr19.51351233.TC.T + 0.0157456158306 * chr19.51358688.C.T + 0.00474425251644 * chr19.51350880.T.A + 0.00334400701562 * chr19.51304104.C.T + 0.00229175502738 * chr19.51305784.C.T + 0.00362599924585 * chr19.51304434.A.G + 0.0159491250027 * chr19.51323881.T.G + 0.0128883191981 * chr19.51323947.A.G + 0.00684026168041 * chr19.51332670.C.T + 0.00308692963367 * chr19.51298912.T.C + 0.00329724597333 * chr19.51297944.A.G + 0.00144739594979 * chr19.51296796.A.T + 0.0018564904141 * chr19.51296698.A.G + 0.00454700579494 * chr19.51296492.T.C + 0.00217690039912 * chr19.51302202.T.A + 0.000790476509333 * chr19.51302473.T.TAAG + 0.00401854055943 * chr19.51348711.C.T + 0.0156087262113 * chr19.51301395.C.T + 0.00553089412199 * chr19.51348572.T.G + 0.00232725099209 * chr19.51301456.G.A + 0.0185343465966 * chr19.51348229.G.C + 0.0948208612213 * chr19.51457477.G.A + 0.0915166955879 * chr19.51457479.C.A + 0.00326334447186 * chr19.51297825.G.A

Gene	**Modeled eQTL Variant(s):**<br>**dbSNP rsid's (top row), and**<br>**Effect size * [chr].[hg19 position].[reference allele].[effect allele] (bottom row)**
	+ 0.0170500351121 * chr19.51297826.C.A + 0.00194302264881 * chr19.51300139.C.T + 0.0751734934729 * chr19.51360418.C.CAA + 2.11805673516 * chr19.51367130.G.A + 0.00285191914505 * chr19.51299645.G.A + 0.000378423374998 * chr19.51377607.A.G + 0.0596297774773 * chr19.51353601.A.G + 0.0161801019435 * chr19.51301230.G.A
TIMM23	rs10994675, rs113007204, rs11548236, rs117273914, rs17720205, rs3813713, rs41306524, rs56148635, rs61847076, rs61847077, rs72795895, rs72795897, rs72797707, rs78937603
	0.0143871572222 * chr10.51563993.G.A + -0.000871296228078 * chr10.51593069.GACA.G + 9.70381305779e-06 * chr10.51589581.C.G + 0.00322934621867 * chr10.51580469.A.C + -0.00294106781801 * chr10.51579413.C.T + 0.00374199298267 * chr10.51571131.C.G + 0.01290561066 * chr10.51582894.C.T + 0.0008502740194 * chr10.51587237.G.A + -0.00168021247519 * chr10.51591402.C.G + -0.000253470364938 * chr10.51591433.C.T + 0.00105413433729 * chr10.51580410.C.A + 0.00103687481119 * chr10.51581889.T.A + 0.000586982824098 * chr10.51592727.C.G + -0.00246296684203 * chr10.51580467.C.G

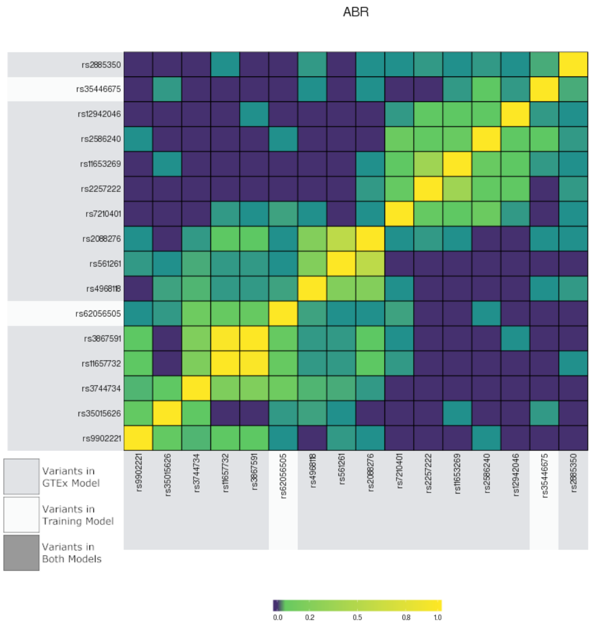
ABR
Variants in GTEx Model
Variants in Training Model
Variants in Both Models
0.0
0.2
0.5
0.8
1.0

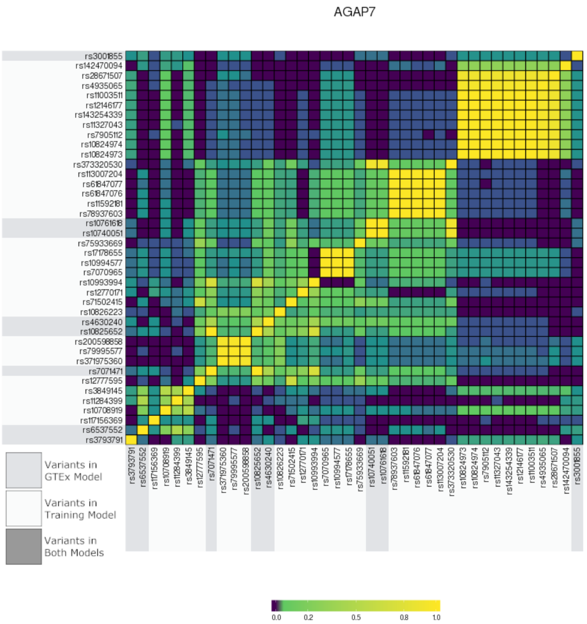
AGAP7
Variants in GTEx Model
Variants in Training Model
Variants in Both Models
0.0
0.2
0.5
0.8
1.0

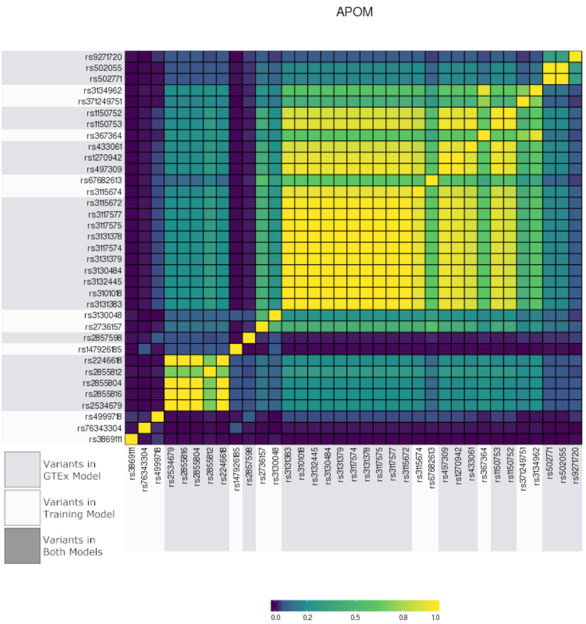
APOM
Variants in GTEx Model
Variants in Training Model
Variants in Both Models
0.0
0.2
0.5
0.8
1.0

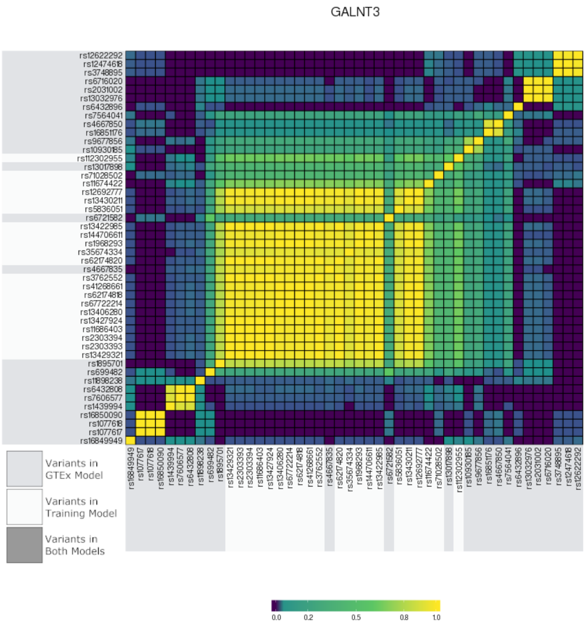
GALNT3
Variants in GTEx Model
Variants in Training Model
Variants in Both Models
0.0
0.2
0.5
0.8
1.0

KCNQ1

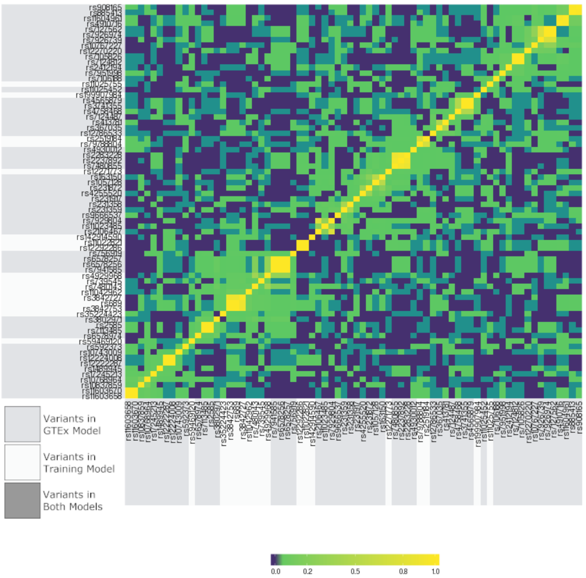

KLK15

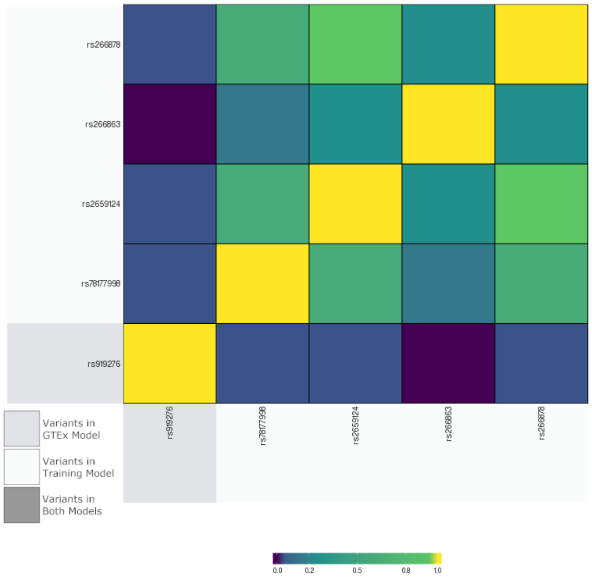

NCOA4

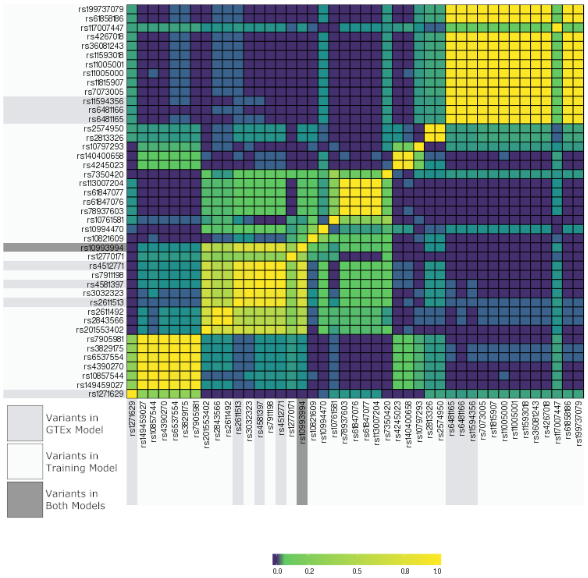

RPS2

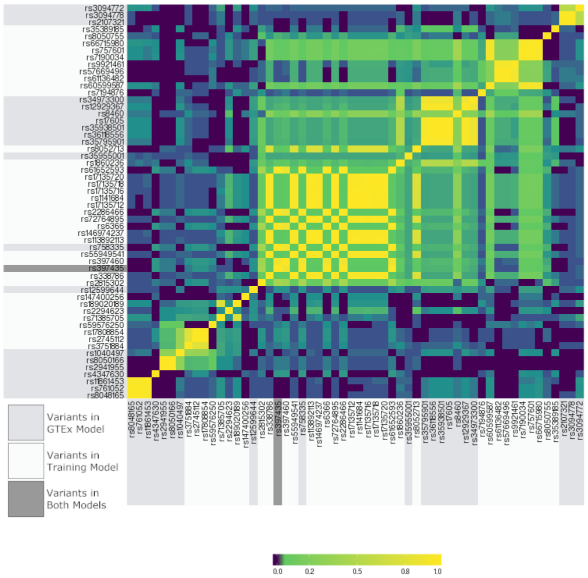

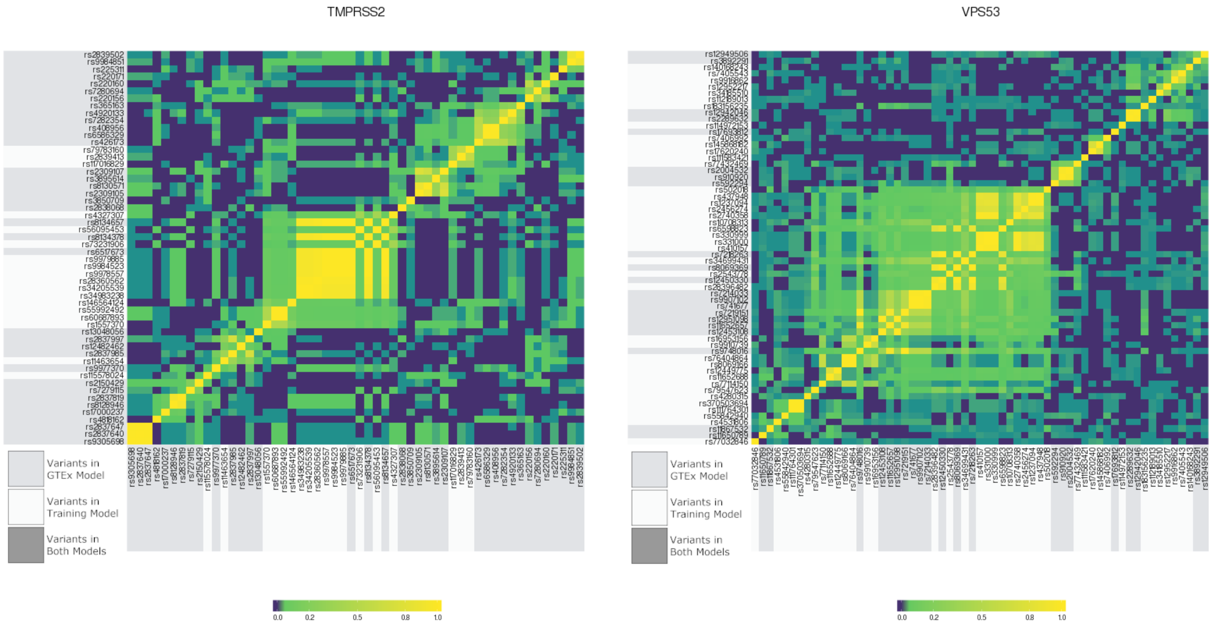

Supplementary Figure 1: Comparison of GTEx and Training Data Model Composition. Heat-maps of linkage disequilibrium (LD) r^2 between eQTL variants modeled in GTEx v6p Prostate PredictDB dataset ("GTEx Model") and those modeled using the dbGaP training data ("Training Model") for this study. The margin colors highlight whether a particular modeled variant derives from the "GTEx Model," "Training Model," or was present in "Both Models" for a particular gene, while the broader LD patterns reveal the extent of similarity or dissimilarity for models developed for the expression levels of the same gene in two different datasets.

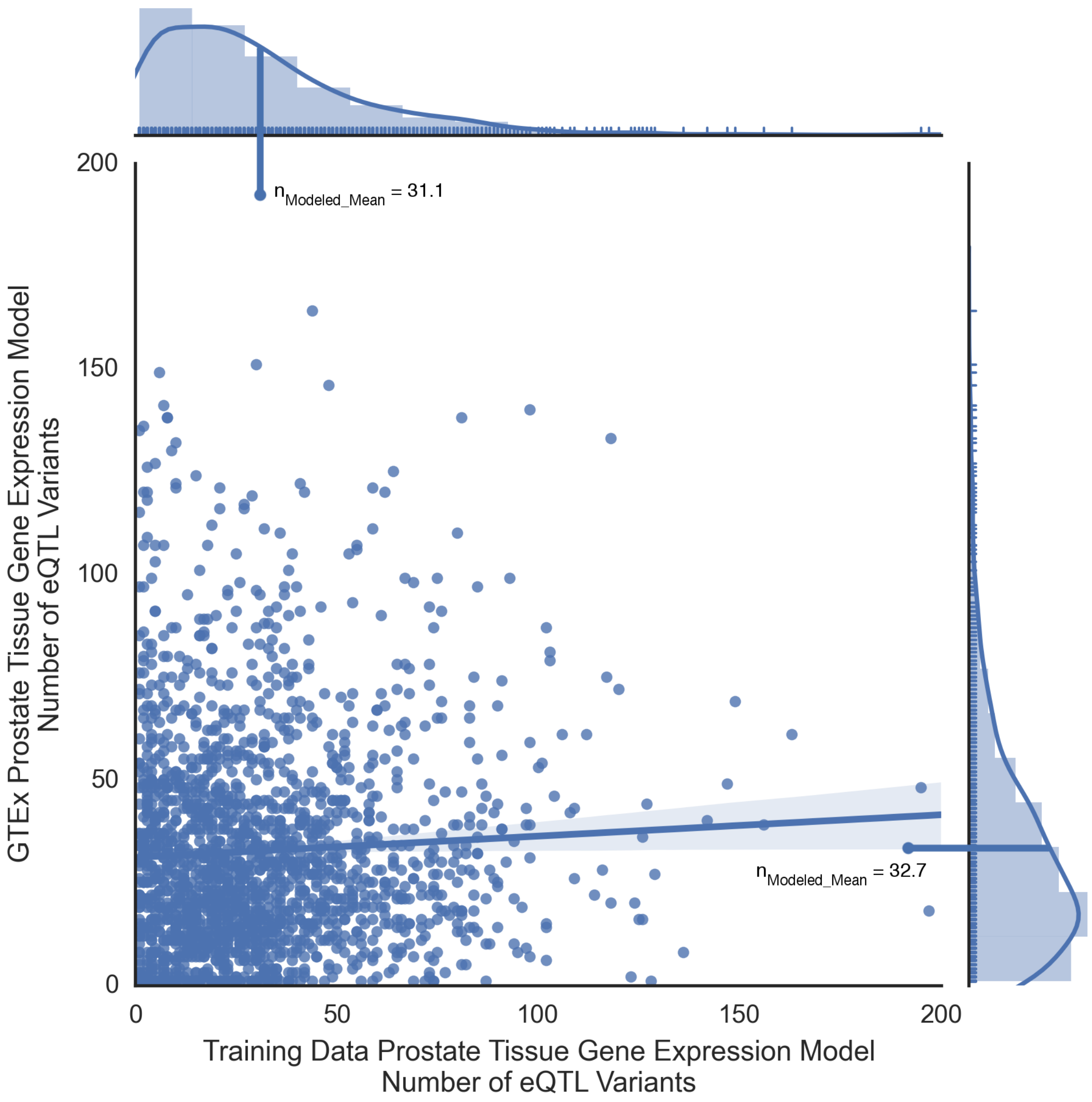

Supplementary Figure 2: Comparison of Number of Modeled eQTL Variants between TWAS Reference Panels. Scatter plot comparison of the cross-validated performance r^2 for 1,884 gene expression models derived from GTEx prostate data (N = 87 subjects) versus the training dataset for the present study (N = 471). In addition to a linear regression line and 95% confidence interval, marginal histograms and density curves are included for both the x-axis (training data model performance) and y-axis (GTEx model performance), with the minimum and mean r^2 values also labeled.

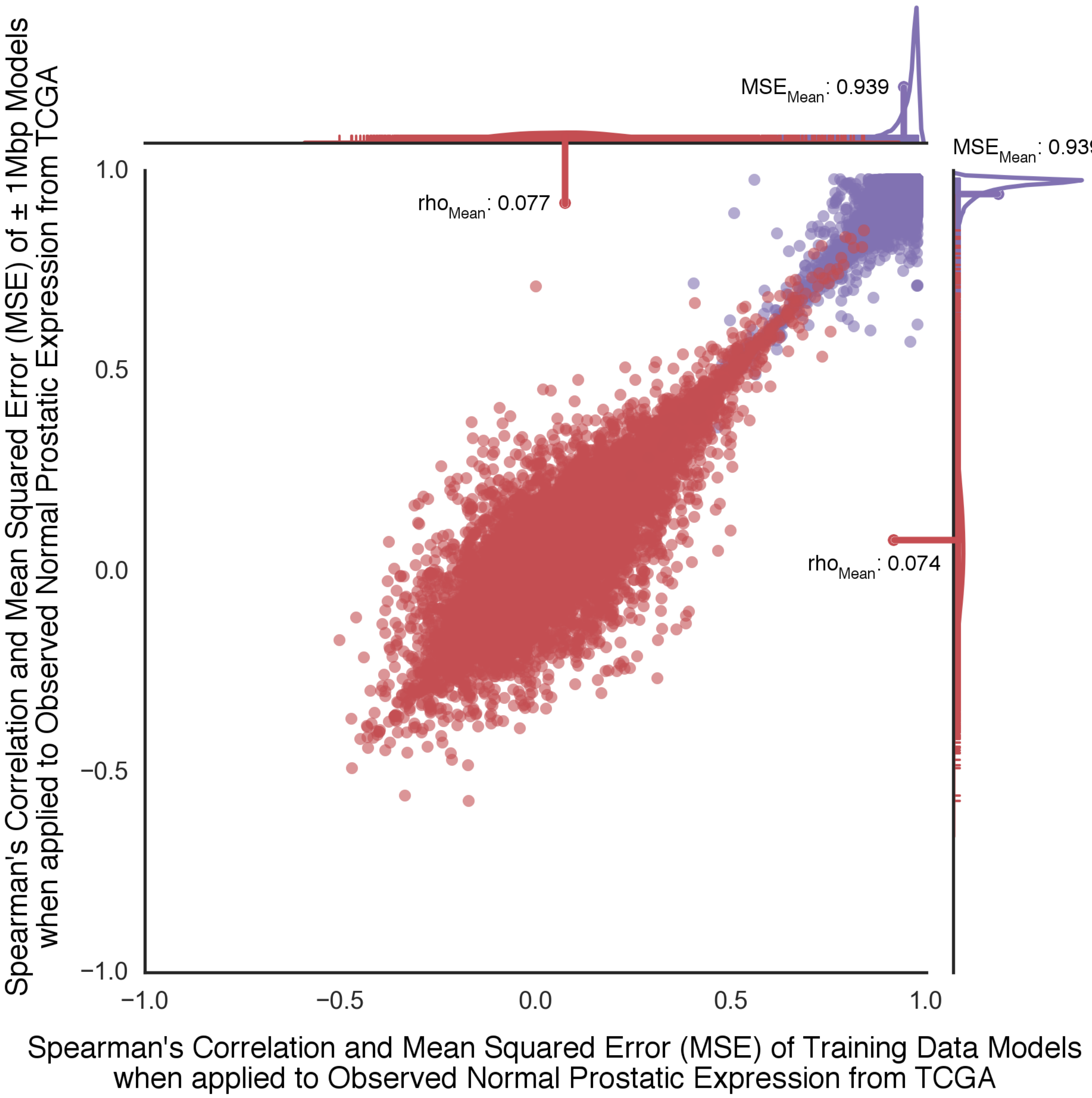

Supplementary Figure 3: Comparison of Model Performance in TCGA Normal Prostate Tissue Expression (N = 45 subjects) for Training Data Models Developed Using Genotypes in ±500kb vs. ±1Mb Windows. Scatter plot comparison of the correlation to observed RNA-seq expression and mean-squared error (MSE) of imputed TCGA normal prostate expression through Elastic Net Leave-One-Out-Cross-Validation (LOOCV) modeling of all variants within one megabase of gene boundaries (Y-axis) versus a half-megabase (X-axis) using the same training dataset of N = 471

prostate tissue samples. The correlation (Spearman's rho) between imputed and observed expression is illustrated in red, while the Mean Squared Error of the predictions is illustrated in violet, both with marginal density curves. Marginal density curves are included with mean values ("MSE_{Mean}", "rho_{Mean}") labeled.

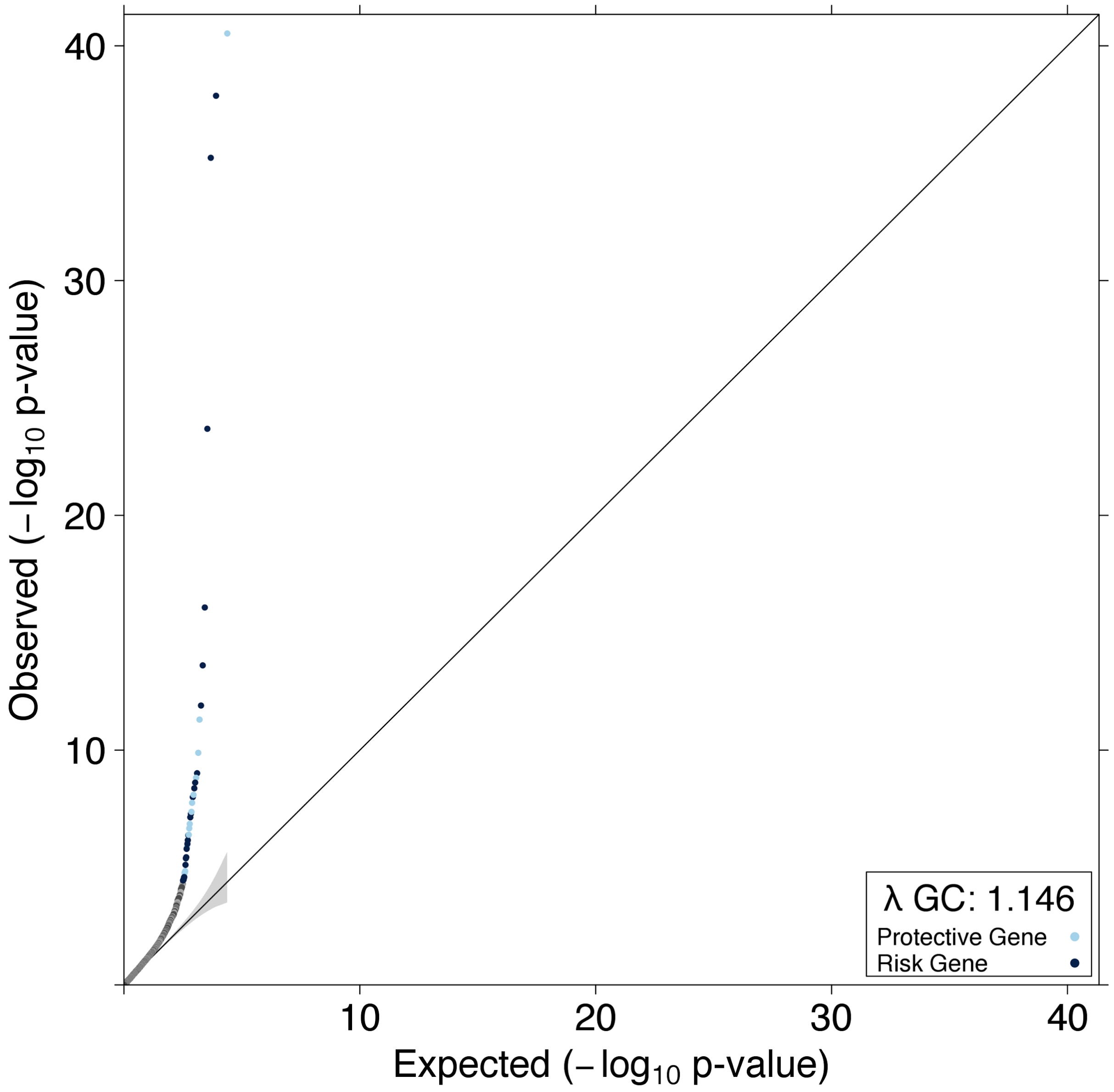

Supplementary Figure 4: Quantile-Quantile Plot of PrCa TWAS Associations from the UK Biobank Discovery Analysis. Comparison of observed versus expected (uniform) distributions of $-\log_{10}$(TWAS *P*-values). Genes with a protective vs. risk direction of effect are illustrated in light blue and navy, respectively, and the 95% confidence interval is depicted in grey. The identity line (y = x) is illustrated in black, and genomic inflation factor λ_{GC} listed in the legend.

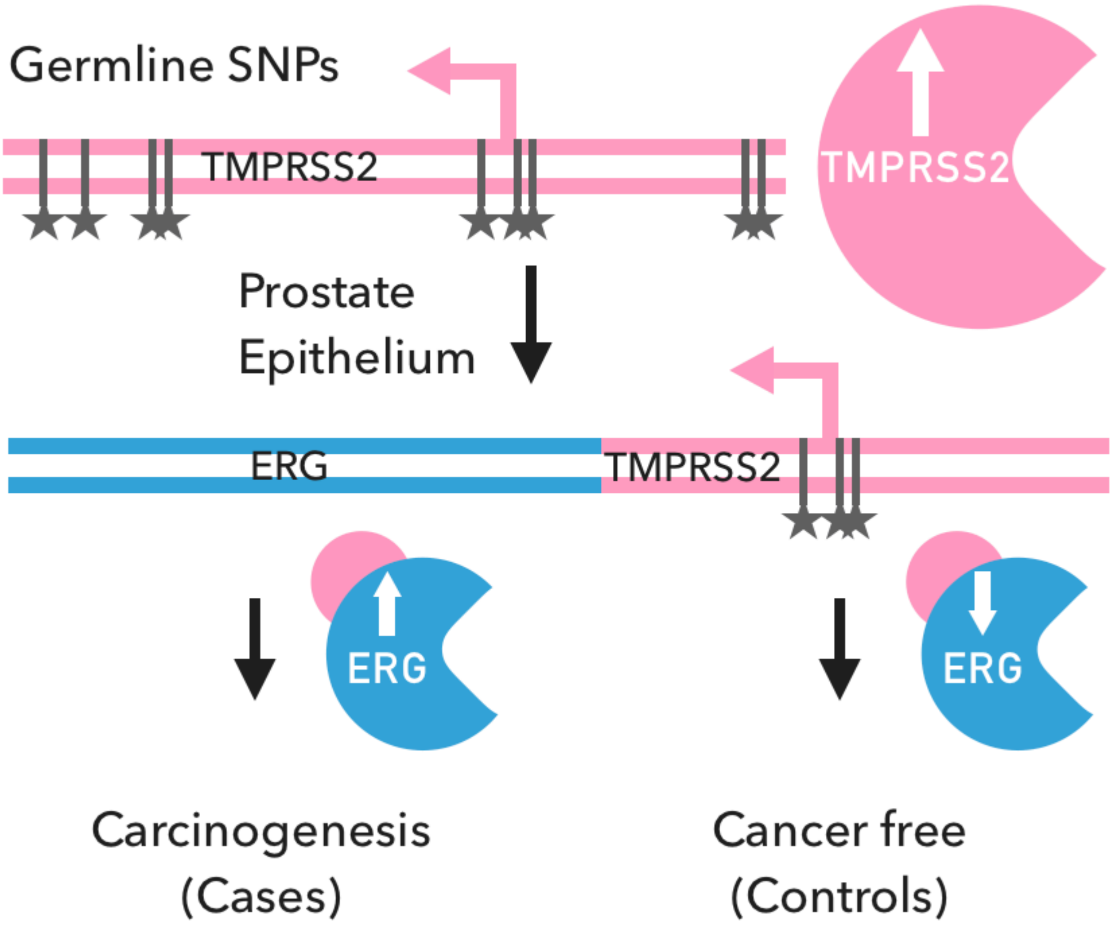

Supplementary Figure 5: Proposed *TMPRSS2*:*ERG* Germline-Somatic Mechanism of Action. Depiction of a germline-somatic mechanism whereby the germline polymorphisms (stars) associated with *TMPRSS2* expression (pink), and modeled in our study to increase the relative expression of *TMPRSS2* in normal prostate tissue, upon fusion with the *ERG* open reading frame (blue), may likewise increase the somatic burden of the *TMPRSS2*:*ERG* fusion oncogene (blue/pink), and hence increase the risk for prostate carcinogenesis.

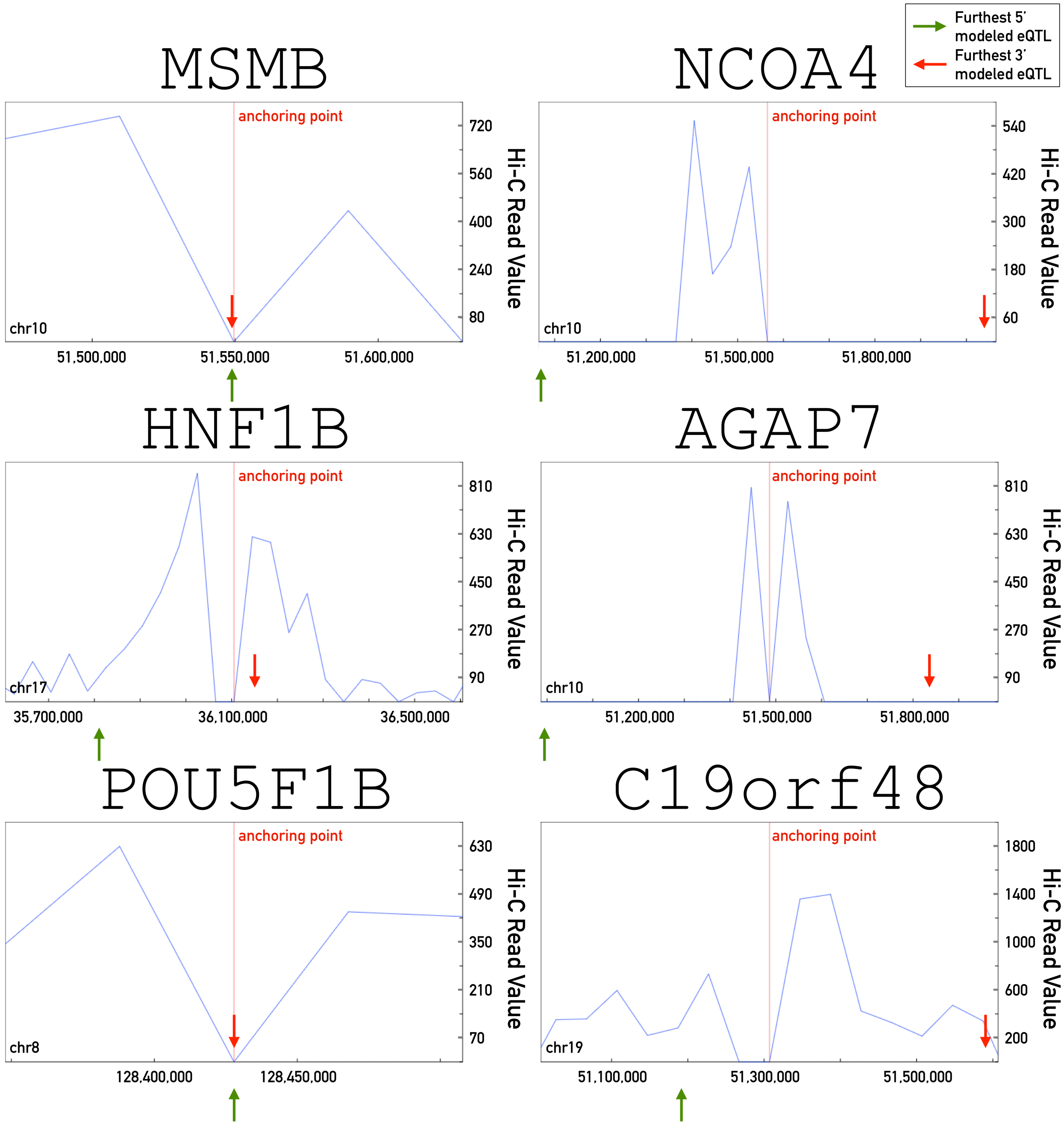
Furthest 5' modeled eQTL
Furthest 3' modeled eQTL
MSMB
anchoring point
chr10
51,500,000
51,550,000
51,600,000
720
560
400
240
80
Hi-C Read Value
NCOA4
anchoring point
chr10
51,200,000
51,500,000
51,800,000
540
420
300
180
60
Hi-C Read Value
HNF1B
anchoring point
chr17
35,700,000
36,100,000
36,500,000
810
630
450
270
90
Hi-C Read Value
AGAP7
anchoring point
chr10
51,200,000
51,500,000
51,800,000
810
630
450
270
90
Hi-C Read Value
POU5F1B
anchoring point
chr8
128,400,000
128,450,000
630
490
350
210
70
Hi-C Read Value
C19orf48
anchoring point
chr19
51,100,000
51,300,000
51,500,000
1800
1400
1000
600
200
Hi-C Read Value

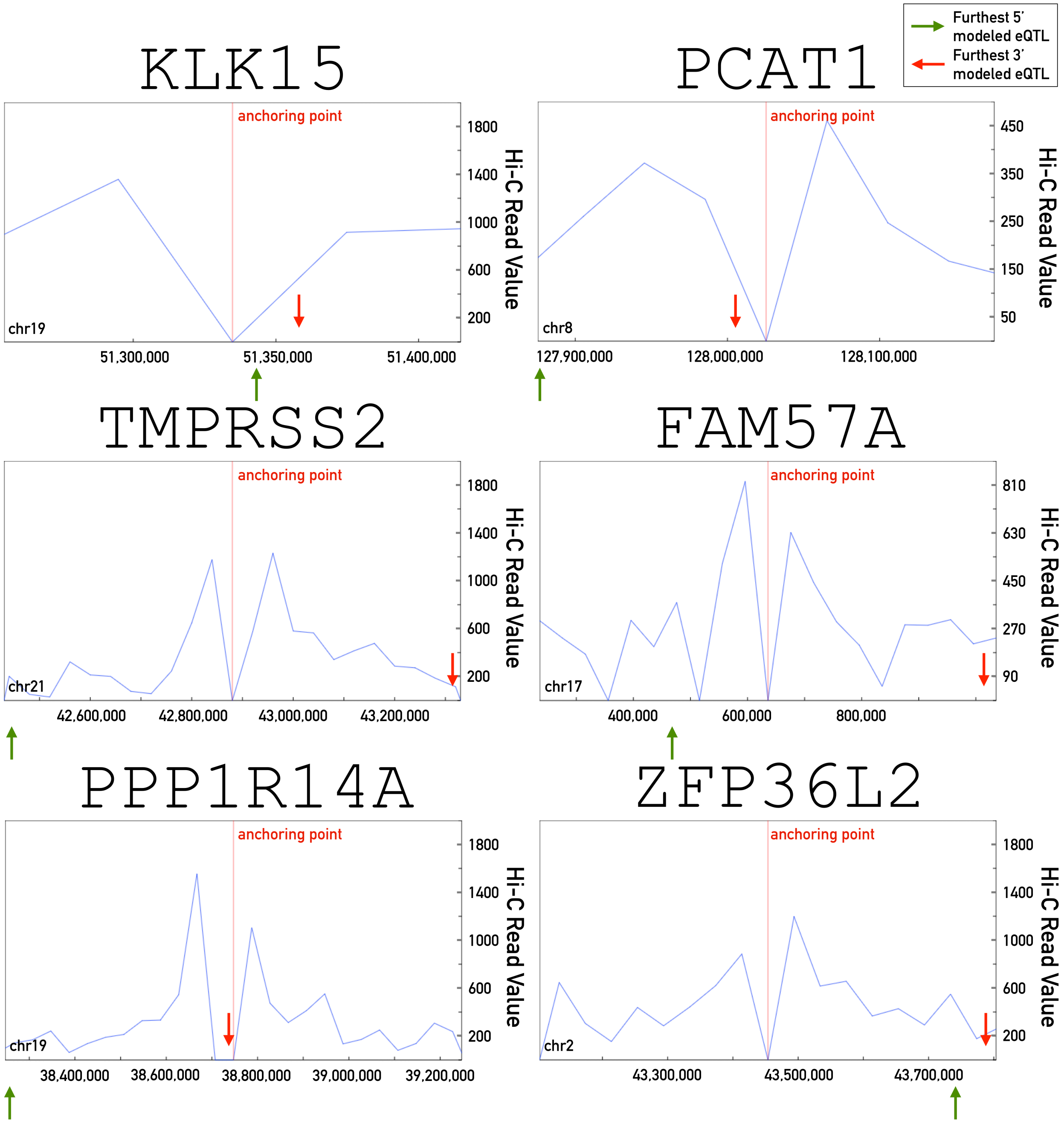
Furthest 5' modeled eQTL
Furthest 3' modeled eQTL
KLK15
anchoring point
chr19
51,300,000
51,350,000
51,400,000
1800
1400
1000
600
200
Hi-C Read Value
PCAT1
anchoring point
chr8
127,900,000
128,000,000
128,100,000
450
350
250
150
50
Hi-C Read Value
TMPRSS2
anchoring point
chr21
42,600,000
42,800,000
43,000,000
43,200,000
1800
1400
1000
600
200
Hi-C Read Value
FAM57A
anchoring point
chr17
400,000
600,000
800,000
810
630
450
270
90
Hi-C Read Value
PPP1R14A
anchoring point
chr19
38,400,000
38,600,000
38,800,000
39,000,000
39,200,000
1800
1400
1000
600
200
Hi-C Read Value
ZFP36L2
anchoring point
chr2
43,300,000
43,500,000
43,700,000
1800
1400
1000
600
200
Hi-C Read Value

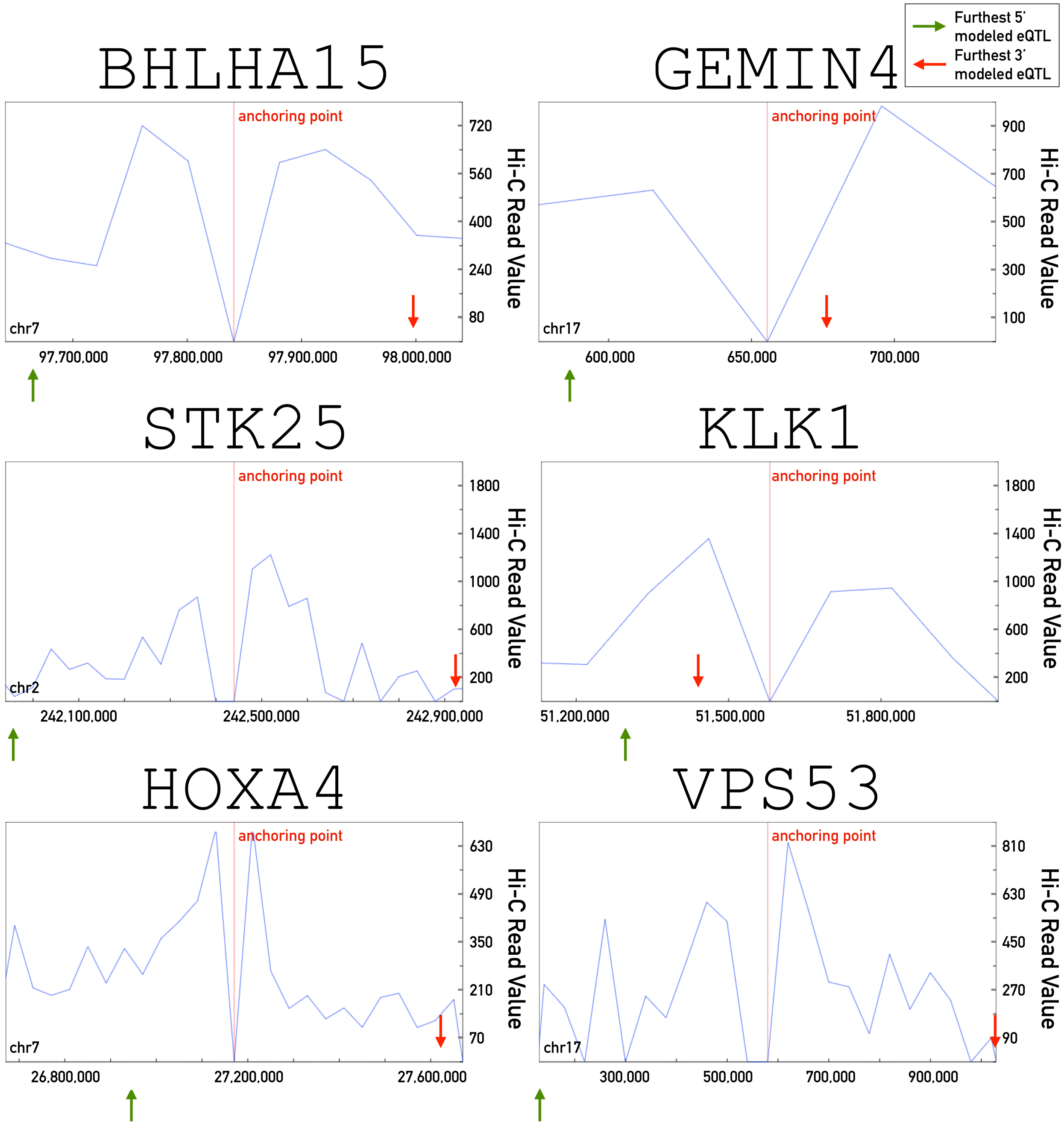
Furthest 5' modeled eQTL
Furthest 3' modeled eQTL
BHLHA15
anchoring point
chr7
97,700,000
97,800,000
97,900,000
98,000,000
720
560
400
240
80
Hi-C Read Value
GEMIN4
anchoring point
chr17
600,000
650,000
700,000
900
700
500
300
100
Hi-C Read Value
STK25
anchoring point
chr2
242,100,000
242,500,000
242,900,000
1800
1400
1000
600
200
Hi-C Read Value
KLK1
anchoring point
51,200,000
51,500,000
51,800,000
1800
1400
1000
600
200
Hi-C Read Value
HOXA4
anchoring point
chr7
26,800,000
27,200,000
27,600,000
630
490
350
210
70
Hi-C Read Value
VPS53
anchoring point
chr17
300,000
500,000
700,000
900,000
810
630
450
270
90
Hi-C Read Value

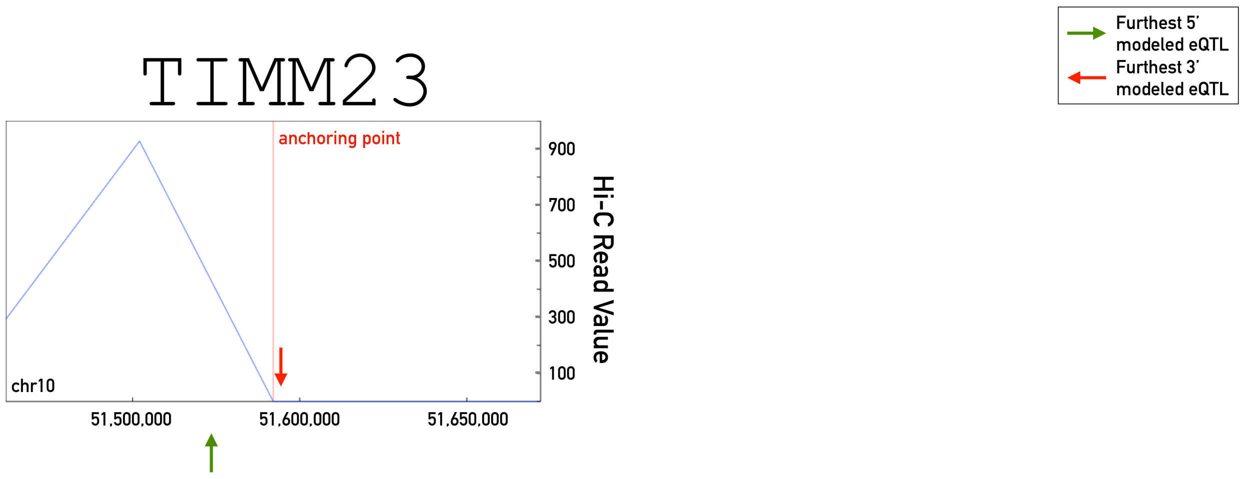

Supplementary Figure 6: Virtual 4C of LNCaP Hi-C Data for Associated Genes of Interest. Illustration of the physical interactions between the promoter region of a given gene and its neighboring genomic loci. Y-axis values (blue) reflect the number of sequencing reads mapped to a given genomic position (X-axis) that were physically ligated to the 40kb window containing the anchoring point (red) locus in chromatin conformation capture (Hi-C) experiment data from the LNCaP prostate cancer cell line. Green and pink arrows illustrate the genomic positions of the furthest upstream and downstream modeled eQTL variants, respectively, predicted to up- or down-regulate expression levels of the particular target gene of interest.

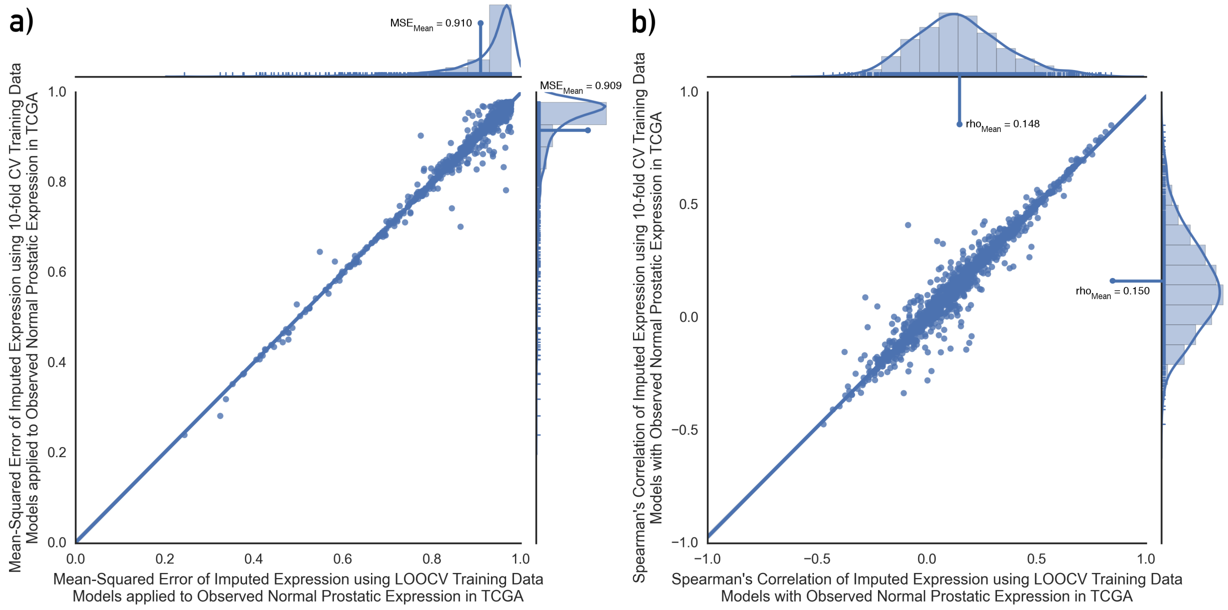

Supplementary Figure 7: Comparison of Model Performance for Leave-One-Out Cross Validation (LOOCV) and 10-fold Cross Validation (CV) Training Data Models, in Application to TCGA Normal Prostate Tissue Expression (N = 45 subjects). 6a. Scatter plot comparison of mean-squared error (MSE) of imputed TCGA normal prostate expression versus observed RNA-seq measurements using 10-fold CV (Y-axis) and LOOCV (X-axis) models developed from training dataset. In addition to a linear regression line and 95% confidence interval, marginal histograms and density curves are included with mean values (“MSE_{Mean}”) labeled. 6b. Scatter plot comparison of Spearman’s correlation between imputed TCGA normal prostate expression and observed RNA-seq measurements using 10-fold CV (Y-axis) and LOOCV (x-axis) models developed from training dataset. In addition to a linear regression line and 95% confidence interval, marginal histograms and density curves are included with mean values (“rho_{Mean}”) labeled.

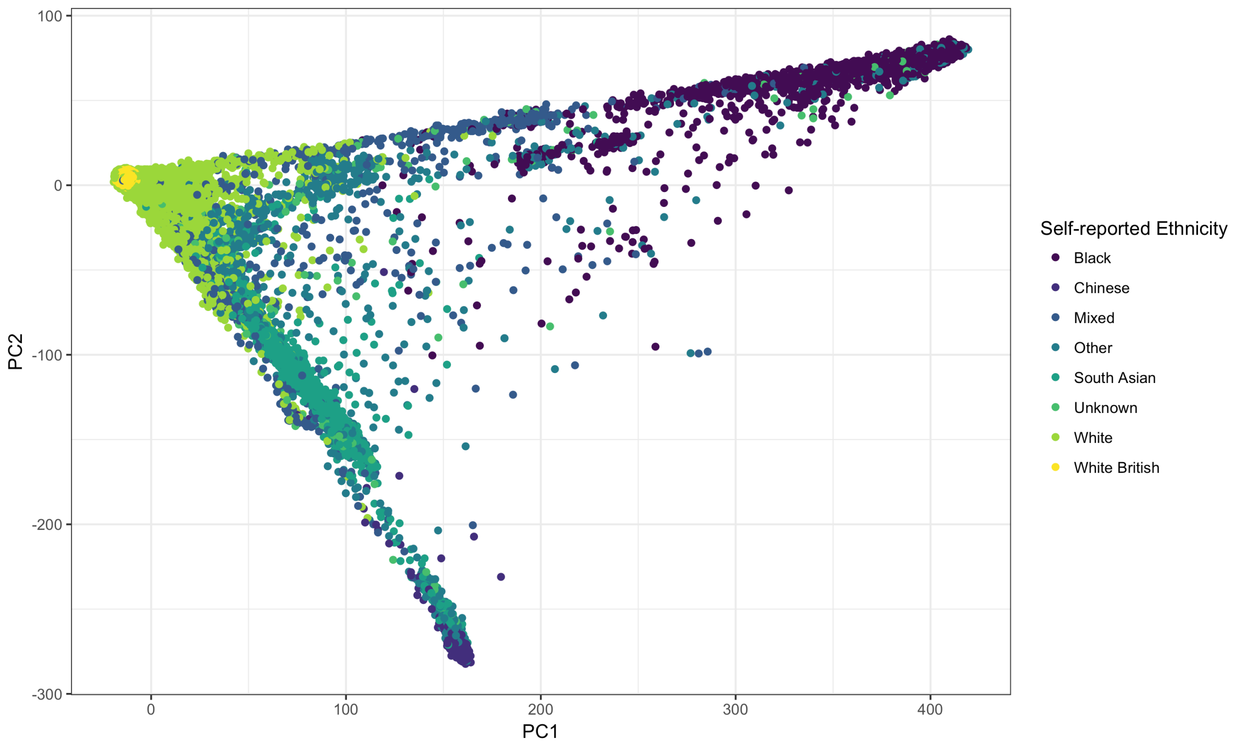

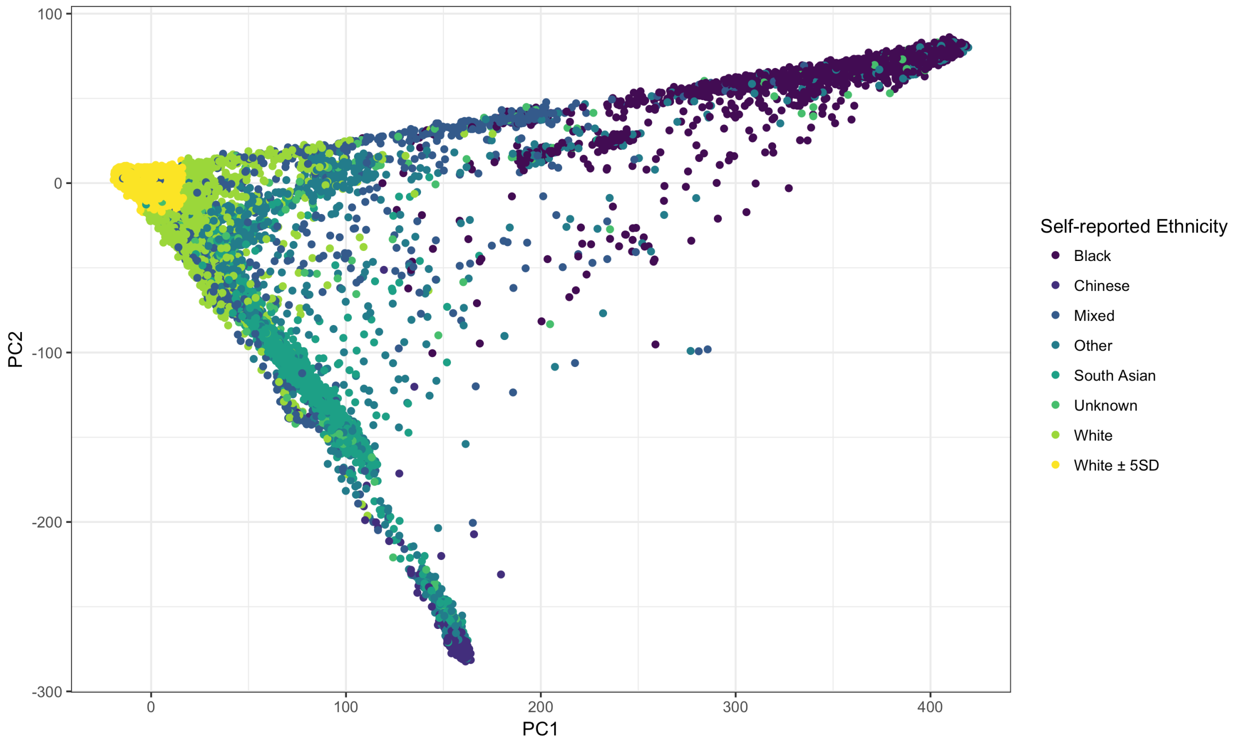

Supplementary Figure 8: Genetic Principal Components for Male UK Biobank Participants Colored by Self-Reported Ethnicity. Illustration of the first (PC1) and second principal component

(PC2) of genetic ancestry for male subjects from the UK Biobank (with both self-identified and genetically inferred gender of male). Top: Joint distribution of PC1 and PC2, with subjects color-coded by self-reported ancestry. Bottom: Illustration of subjects (yellow) within 5 standard deviations of the PC1 and PC2 means for self-identified “White” subjects that were carried into the discovery analysis.
